# NLRC4-mediated activation of CD1c^+^ DC contributes to perpetuation of synovitis in rheumatoid arthritis

**DOI:** 10.1172/jci.insight.152886

**Published:** 2022-11-22

**Authors:** Cristina Delgado-Arévalo, Marta Calvet-Mirabent, Ana Triguero-Martínez, Enrique Vázquez de Luis, Alberto Benguría-Filippini, Raquel Largo, Diego Calzada-Fraile, Olga Popova, Ildefonso Sánchez-Cerrillo, Ilya Tsukalov, Roberto Moreno-Vellisca, Hortensia de la Fuente, Gabriel Herrero-Beaumont, Almudena Ramiro, Francisco Sánchez-Madrid, Santos Castañeda, Ana Dopazo, Isidoro González Álvaro, Enrique Martin-Gayo

**Affiliations:** 1 Immunology Unit from Hospital Universitario La Princesa, Medicine Faculty, Autonomous University of Madrid (UAM), Instituto Investigación Sanitaria-Princesa IIS-IP, Madrid, Spain.; 2 Rheumatology Department from Hospital Universitario La Princesa, Instituto de Investigación Sanitaria-Princesa IIS-IP, Madrid, Spain.; 3 Genomic Unit, The National Centre for Cardiovascular Research, Madrid, Spain.; 4 Bone and Joint Research Unit, Rheumatology Service, IIS Fundación Jiménez Díaz, Madrid, Spain.; 5 CIBER Cardiovascular, Madrid, Spain.; 6 Biology Laboratory, The National Centre for Cardiovascular Research, Madrid, Spain.; 7 Cátedra UAM-Roche, EPID-Future, Department of Medicine, UAM, Madrid, Spain.; 8 CIBER Infectious Diseases, Madrid, Spain.

**Keywords:** Autoimmunity, Cell Biology, Autoimmune diseases, Dendritic cells, Innate immunity

## Abstract

The individual contribution of specific myeloid subsets such as CD1c^+^ conventional DC (cDC) to perpetuation of rheumatoid arthritis (RA) pathology remains unclear. In addition, the specific innate sensors driving pathogenic activation of CD1c^+^ cDC in patients with RA and their functional implications have not been characterized. Here, we assessed phenotypical, transcriptional, and functional characteristics of CD1c^+^ and CD141^+^ cDC and monocytes from the blood and synovial fluid of patients with RA. Increased levels of CCR2 and the IgG receptor CD64 on circulating CD1c^+^ cDC was associated with the presence of this DC subset in the synovial membrane in patients with RA. Moreover, synovial CD1c^+^ cDC are characterized by increased expression of proinflammatory cytokines and high abilities to induce pathogenic IFN-γ^+^IL-17^+^CD4^+^ T cells in vitro. Finally, we identified the crosstalk between Fcγ receptors and NLRC4 as a potential molecular mechanism mediating pathogenic activation, CD64 upregulation, and functional specialization of CD1c^+^ cDC in response to dsDNA-IgG in patients with RA.

## Graphical abstract

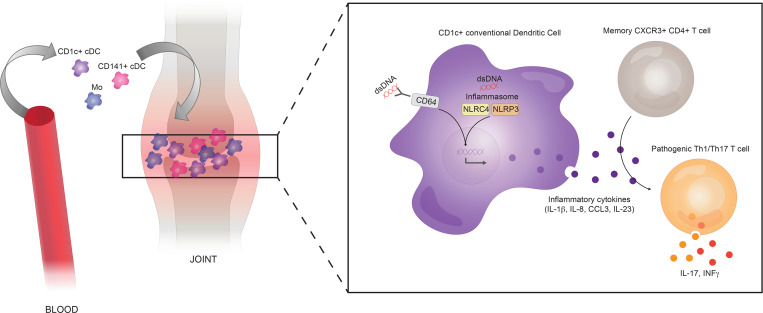

## Introduction

Rheumatoid arthritis (RA) pathogenesis is a multifactorial process that involves the crosstalk between multiple adaptive and innate immune cell subsets leading to chronic synovitis and the progressive destruction of joint cartilage and bone tissue. Altered adaptive immune responses in patients with active RA disease mediated by autoantibody-producing B cells (1–3), Th1 and Th17 CD4^+^ T cells (4, 5), and activated cytotoxic CD8^+^ T cells (6) have been well characterized. However, less is known about the contribution of specific innate cell populations to perpetuate chronic inflammation and the induction of pathogenic CD4^+^ T cells able to produce both IL-17 and IFN-γ (known as Th1/Th17 cells), a T cell subset that is enriched in synovial fluid (SF) of patients with RA (7) and has been linked to severity of multiple autoimmune disorders (8–10). In this regard, deregulation of myeloid cells such as monocytes (Mo) and DC can lead to the development of autoimmunity (11, 12). However, the heterogeneity of Mo and DC lineages has made difficult to fully understand the contribution of individual cell subsets to RA pathology. Several studies have reported alterations in Mo subset phenotype and function in the peripheral blood (PB) and SF from patients with RA (13–15), along with their participation in the erosion of juxta-articular bone (14, 16). In contrast, less is known about the contribution of different subtypes of DC to RA immunopathology (17).

DC can be divided into 2 main subgroups, conventional DC (cDC) and plasmacytoid DC (pDC), with different functional specializations (18, 19). pDC physiologically mediate type I IFN responses in the context of viral infections (20) but have also been involved in autoimmune disorders, such as systemic lupus erythematosus and psoriasis (20). In addition, pDC appear to play a tolerogenic role on RA joint inflammation (21, 22). In contrast, cDC can be subdivided into CD141^+^ and CD1c^+^ cDC with differential abilities to efficiently activate CD8^+^ and CD4^+^ T cell responses, respectively (23, 24). Frequencies of both CD141^+^ and CD1c^+^ cDC have been reported to be reduced in the PB and enriched in the SF of patients with RA. Moreover, CD141^+^ and CD1c^+^ cDC in SF of RA individuals can induce IFN-γ^+^ and TNF-α^+^ CD4^+^ T cell (22, 25) or IL-17 secretion by T cells in vitro (26) in individual studies. However, potential differences in functional capacities of both cDC subsets and in Mo from patients with RA to induce pathogenic IL-17^+^IFN-γ^+^ T cells have not been directly addressed. In addition, the molecular mechanisms specifically affecting phenotypical and functional properties of CD1c^+^ cDC in patients with RA and the functional implications of these alterations have not been characterized. Previous genome-wide association studies (GWAS) identified genetic variations related to class II-HLA, TNF-α, Fc-receptor (FcR), toll-like receptor (TLR), and nucleic acid sensing pathways that are associated with increased risk of developing RA (27–30). Several studies have also suggested that recognition of endogenous DNA and RNA as DAMPs by nucleic-acid sensors might induce innate responses that contribute to the development of autoimmunity, including RA (31). In addition, activation of alternative innate pathways such as the NLRP3 inflammasome has been proposed as a pathogenic activation mechanism of Mo in RA (32, 33). However, it is unknown whether common or different innate sensors may differentially mediate pathogenic activation of CD1c^+^ cDC and other myeloid cells in RA. In fact, the most accepted treatments nowadays are based on the blockade of inflammatory cytokines or their receptors, which are not always effective (34, 35). Therefore, it is critical to identify innate sensors that might be specifically mediating pathogenic activation in different myeloid subsets in RA, in order to design more targeted and effective therapies.

The objective of our study was to specifically investigate the contribution of CD1c^+^ cDC to chronic disease perpetuation and the mechanism of pathogenic activation of these cells in RA. Our phenotypical, transcriptional, and functional analysis identified CD64 and CCR2 as markers of activated migratory CD1c^+^ cDC enriched in the inflamed joint from patients with RA, which are selectively restored in the PB after treatment initiation and reduction of clinical severity. In addition, CD1c^+^ cDC from the SF of patients with RA are characterized by preferential expression of IL-1β, IL-8, and CCL3 and by higher functional abilities to induce pathogenic IL-17^+^IFN-γ^+^ T cell responses in vitro compared with other synovial myeloid subsets. Interestingly, inflammatory and functional RA-like properties could be induced in vitro on CD1c^+^ cDC by incubation with dsDNA-IgG complexes. Remarkably, we have identified the NLRC4 as a sensor required for FcγR-mediated detection of dsDNA-IgG complexes, thereby inducing Caspase 1–dependent inflammasome activation of CD1c^+^ cDC subset. Collectively, our translational study provides evidence of active contribution of CD1c^+^ cDC to RA disease progression and identifies therapeutic target candidates that might be useful for targeted therapies for RA.

## Results

### Frequencies of CD64^+^CD1c^+^ cDC in the blood are restored in treated patients with RA with reduced disease activity.

**Figure 1:**
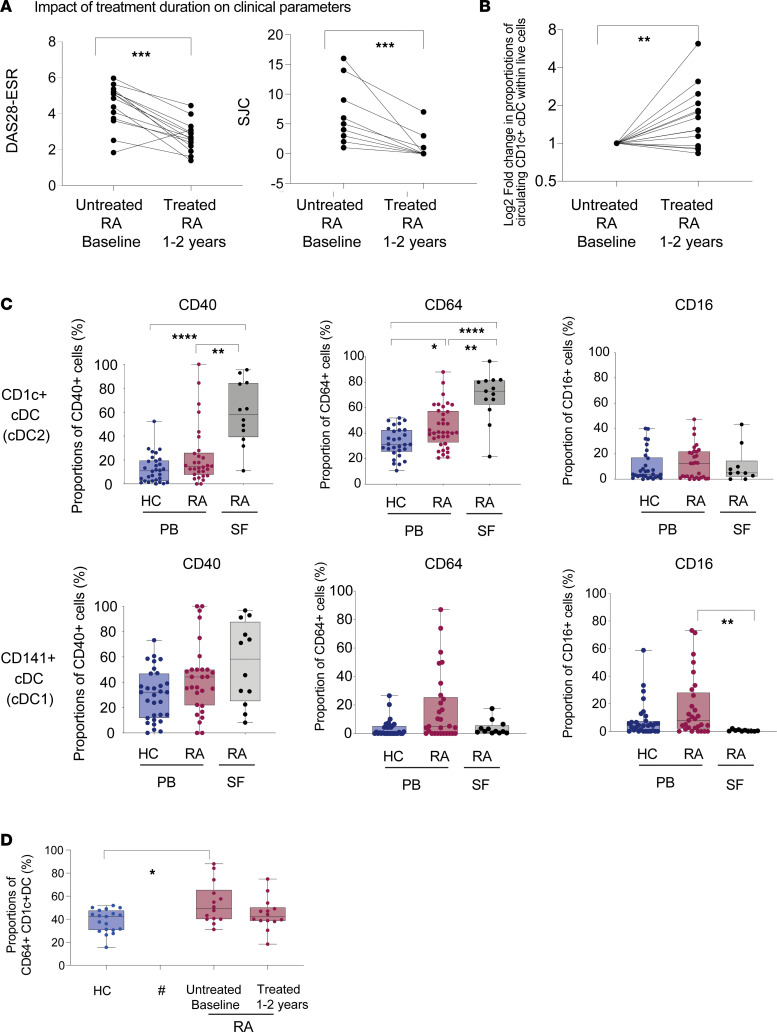
Alterations in frequencies and expression of CD64 in DC subsets present in peripheral blood and synovial fluid from patients with RA. (**A** and **B**) Analysis of DAS28-ESR and number of swollen joint count (SJC) (**A**) or proportions of CD1c^+^ cDC (**B**) in blood samples from *n* = 31 patients with RA collected at the first visit (untreated baseline) and after 1 or 2 years of treatment. Statistical significance was calculated using a 2-tailed matched-pairs Wilcoxon test. (**C**) Proportions of CD40 (left), CD64 (center), and CD16 (right) on gated CD1c^+^ cDC (upper plots) and CD141^+^ cDC (lower plots) from the blood of healthy controls (HC, *n* = 28) and untreated patients with RA (*n* = 31) and SF from treated patients with RA (*n* = 12). Statistical significance was calculated using a Kruskal-Wallis test with Dunn’s correction. (**D**) Proportions of CD64^+^ cells within circulating CD1c^+^ cDC from the blood of *n* = 19 HC and *n* = 14 patients with RA at baseline and 1–2 years after treatment initiation. Statistical significance was calculated using a 2-tailed Mann Whitney *U* test.

Proportions of CD1c^+^ and CD141^+^ cDC, and to a lower extent pDC, were markedly reduced in the PB of 31 patients with RA recruited prior to initiating treatment and compared with 30 healthy controls (HC) including 13 age- and sex-matched individuals (Supplemental Figure 1, A and B, and Supplemental Table 1; supplemental material available online with this article; https://doi.org/10.1172/jci.insight.152886DS1), in line with previous studies (25). In contrast, frequencies of classical (C), transitional (T), and nonclassical (NC) Mo were not significantly different between these 2 cohorts (Supplemental Figure 1B). Of these populations, T-Mo and both CD1c^+^ and CD141^+^ cDC subsets were more significantly enriched in SF from patients with RA obtained during flares despite they were receiving treatment (Supplemental Figure 1B and Supplemental Table 2). We next analyzed the evolution of proportions of circulating cDC and Mo subsets in *n* = 14 patients with RA in a longitudinal follow-up study after treatment for either 1 or 2 years (Supplemental Table 3). We observed that, in treated patients with RA experiencing improvement of clinical values over time, such as lower number of swollen joints and lower disease activity assessed by DAS28-ESR score (Figure 1A), proportions of circulating CD1c^+^ cDC were more significantly recovered (nominal *P* = 0.0067; FDR-corrected *P* = 0.0335) (Figure 1B). Proportions of CD1c^+^ cDC were not significantly associated with age on the RA and HC cohorts (Supplemental Figure 1C). In contrast, frequencies of circulating Mo and CD141^+^ cDC were not significantly affected in these treated individuals (Supplemental Figure 1D). Therefore, CD1c^+^ cDC might be differentially altered in patients with RA. A phenotypical analysis of circulating myeloid subsets showed a nonsignificant trend to increased expression of CD40 on cDC (Figure 1C). However, expression of CD40 was significantly upregulated in CD1c^+^ cDC from SF, and it also tended to be increased in CD141^+^ cDC from this location (Figure 1C). In addition, we also identified a trend of increased CD86 levels in circulating CD1c^+^ and CD141^+^ cDC and NC-Mo, and this increase was not observed in pDC or in SF Mo subsets from patients with RA (Supplemental Figure 2, A–C). Remarkably, PB and SF CD1c^+^ — but not CD141^+^ cDC — showed significantly higher expression of CD64 (Figure 1C). Proportions of CD64^+^ cells in CD1c^+^ cDC were not significantly associated with higher disease activity (Supplemental Figure 2D). Interestingly, CD64 expression levels tended to remain upregulated in CD1c^+^ cDC even in treated patients with RA (Figure 1D). In contrast, CD64 expression was basally the highest in C-Mo but did not significantly increase in circulating cells. On the other hand, CD64 was significantly elevated on T- and NC-Mo from SF (Supplemental Figure 2B), in agreement with previous studies (36). In addition, no alterations in expression of alternative FcRs such as CD16 on CD1c^+^ cDC, CD141^+^ cDC, or Mo were detected, while pDC displayed a mild increase in patients with RA (Figure 1C and Supplemental Figure 2, A and B). Therefore, differential maturation programs might be taking place in CD1c^+^ cDC compared with CD141^+^ cDC and Mo. Together, our data indicate that CD1c^+^ cDC from RA individuals are preferentially restored after treatment initiation and are characterized by the differential expression of the cell surface marker CD64, suggesting a significant contribution of this DC subset to the perpetuation of RA pathology.

### Specific transcriptional profiles of innate activation in CD1c^+^ cDC in patients with RA.

**Figure 2:**
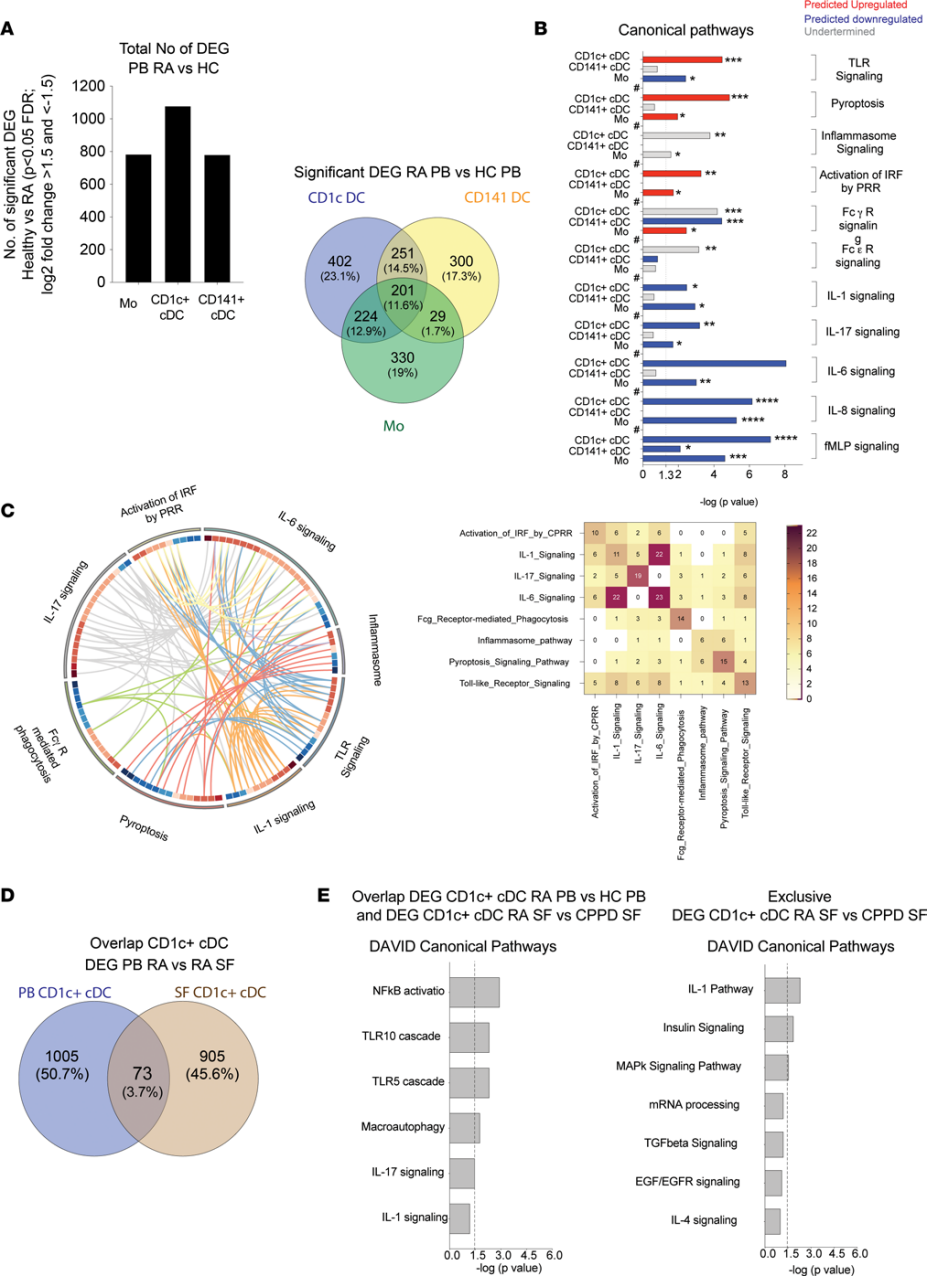
RNA-Seq analysis of differential transcriptional signatures in circulating and synovial Mo, CD1c^+^, and CD141^+^ cDC from patients with RA. (**A**) Number of individual (left) and overlapping (center, Venn diagram) significant differentially expressed genes (DEG *P* < 0.05 after FDR correction considering a log_2_ fold change [log_2_FC] > 1.5 and < –1.5) between circulating Mo, CD1c^+^, and CD141^+^ cDC from *n* = 4 untreated patients with RA compared with *n* = 4 healthy controls (HC). (**B**) Significance of selected upregulated (positive *Z* score; red), downregulated (negative *Z* score; blue), or undetermined (0 or not predicted *Z* score; gray) canonical pathways predicted by IPA (full analysis shown in Supplemental Table 5) for DEG from Mo, CD1c^+^ cDC, and CD141^+^ cDC from RA versus HC. *P < 0.05; **P < 0.021; ***P < 0.001; ****P < 0.0001. (**C**) Circos plot analyzing level of connection and shared genes between some of the pathways significantly altered in CD1c^+^ cDC from the blood of patients with RA shown in **B**. Genes within each pathway are ordered according to upregulated (red scale) and downregulated (blue scale) transcriptional levels. Quantification of interactions between pathways in the circos plot is illustrated on the heatmap shown on the right. (**D**) Venn diagram of overlapping significant DEG in CD1c^+^ cDC from the peripheral blood (PB) and synovial fluid (SF) from RA individuals compared with healthy controls (HC) or calcium pyrophosphate deposition (CPPD) crystal–associated arthropathy patients. (**E**) Significance of selected pathways for 73 overlapping DEG and 905 nonoverlapping DEG in CD1c^+^ cDC from SF mentioned in **D**, predicted by DAVID.

Next, differential transcriptional patterns of circulating CD1c^+^ and CD141^+^ cDC and Mo from the PB of *n* = 4 patients with RA and *n* = 4 HC were characterized. Principal component analysis (PCA) of detected genes suggests that each cell subset in RA was transcriptionally different from its corresponding HC (Supplemental Figure 3). A comparative gene expression analysis considering FDR-corrected significant *P* values and changes in log_2_ fold change of expression over 1.5 or less than –1.5 identified a total of 784; 1,078; and 781 significant differentially expressed genes (DEG) in Mo, CD1c^+^, and CD141^+^ cDC from RA compared with HC, respectively (Figure 2A and Supplemental Table 4). Importantly, a portion of 251 and 224 DEG from CD1c^+^ cDC overlapped with those present in CD141^+^ cDC and with Mo, respectively (Figure 2A), while 402 DEG were exclusively detected in CD1c^+^ cDC. A low level of DEG overlap (29 genes) was observed between Mo and CD141^+^ cDC (Figure 2A). Ingenuity pathway analysis (IPA) identified differences in relevant pathways associated with innate activation enriched in DEG in the 3 myeloid subsets from patients with RA (Figure 2B and Supplemental Table 5). Interestingly, genes related to TLR stimulation, pyroptosis, the inflammasome, FcγR and FcεR signaling, activation of PRR, and signaling of inflammatory cytokines such as IL-1β, IL-6, IL-8, and N-Formyl-methionine-leucyl-phenylalanine (fMLP) were more significantly predicted to be affected in CD1c^+^ cDC compared with other myeloid subsets from PB of RA individuals (Figure 2B and Supplemental Table 5). Paradoxically, some inflammatory cytokine signaling pathways appeared to be mainly downregulated in these cells from PB in patients with RA (Figure 2B), but some components of these and other innate pathways such as TLR and FcR remained upregulated (Supplemental Table 4 and Supplemental Figure 4, A–C). Importantly, these predicted pathways on CD1c^+^ cDC were highly interconnected and appeared to share a significant number of DEG (Figure 2C, left). The pathways sharing the highest number of DEG were TLR/IL-1, TLR/pyroptosis, pyroptosis/inflammasome, and IL-1/IL-6 (Figure 2C, right).

To better understand how transcriptional profiles of circulating CD1c^+^ cDC were related to the patterns present in the same cell subsets infiltrated in SF from RA individuals, we performed an additional RNA-Seq analysis of *n* = 3 RA and *n* = 3 calcium pyrophosphate deposition (CPPD) crystal–associated arthritis patients (Supplemental Table 2). Given the low number and the similar inflammatory nature of both types of SF samples used, we considered nominal *P* < 0.05 as a significance cut-off to identify DEG for each myeloid subset from RA (Supplemental Table 6). In these analyses, a higher but limited overlap of DEG was observed between CD1c^+^ cDC and Mo, compared with CD141^+^ cDC (Supplemental Figure 5). When the lists of DEG obtained for each cell subset in blood and SF were compared (Supplemental Tables 4 and 6), 73 overlapping DEG in CD1c^+^ cDC were detected (Figure 2D), which were also enriched in TLR and IL-1–related pathways in CD1c^+^ cDC from both blood and SF in patients with RA (Figure 2E). However, nonoverlapping transcriptional patterns in CD1c^+^ cDC from SF and PB more significantly predicted alterations in the IL-1 pathway (Figure 2E), indicating different components of the pathway enriched in cells from these 2 locations. Therefore, CD1c^+^ cDC from the PB and SF of patients with RA are characterized by specific transcriptional signatures associated to TLR, inflammasome, and proinflammatory cytokines.

### Identification of CCR2 as a marker for migratory CD64^hi^CD1c^+^ cDC in patients with RA.

**Figure 3:**
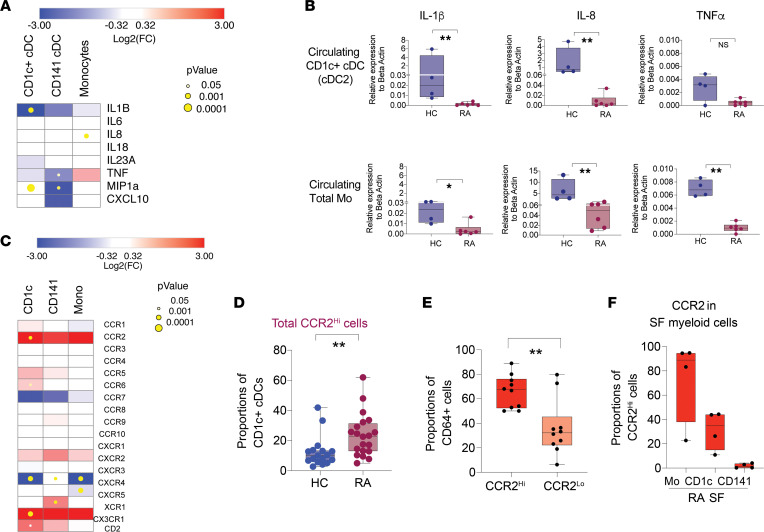
Expression of CCR2 on CD1c^+^ cDC associates with depletion of CD64^hi^-activated cells from the blood of patients with RA. (**A** and **C**) Heatmaps reflecting log_2_FC in the transcription of inflammatory cytokines downstream TLR and inflammasome (**A**) or the indicated chemokine receptors (**C**) in circulating CD1c^+^ cDC (blue bars), CD141^+^ cDC (red bars), and Mo (cyan bars) from peripheral blood (PB) of *n* = 4 patients with RA versus *n* = 4 healthy controls. Size of yellow circles represents different levels of statistical significance. (**B**) qPCR analysis of expression of some of the cytokines identified in **A** relative to β-actin levels in PB CD1c^+^ cDC (upper plots) and Mo (lower plots) from *n* = 4 healthy controls (HC) compared with *n* = 5 patients with RA. (**D**) Proportions of total CCR2^hi^ cells included in the CD1c^+^ cDC from the blood of *n* = 20 patients with RA and *n* = 17 HC. Statistical significance was calculated using a 2 tailed Mann Whitney *U* test. (**E**) Proportions of CD64^+^ cells present on gated CCR2^lo^ or CCR2^hi^ subpopulations of CD1c^+^ cDC from the blood of patients with RA. (**F**) Proportions of CCR2^+^ cell from synovial fluid (SF) Mo and CD1c^+^ and CD141^+^ cDC from patients with RA (*n* = 4, red). Data represent mean and SEM values. **P < 0.01.

We subsequently analyzed the expression of inflammatory cytokines downstream from the TLR and inflammasome pathways in myeloid subsets from RA individuals. Interestingly, RNA-Seq data and quantitative PCR (qPCR) validation indicated that expression of proinflammatory cytokines such as IL-1β, IL-8, and MIP1α (CCL3) was downregulated in circulating CD1c^+^ cDC, CD141^+^ cDC, and Mo from patients with RA compared with HC (Figure 3, A and B, and Supplemental Figure 6A). These data may indicate selective migration of activated inflammatory cDC and Mo from the blood to other anatomical locations — such as the synovial membrane — in patients with RA. Thus, we analyzed the transcriptional expression of chemokine receptors that might be differentially expressed in circulating cDC subsets and Mo from patients with RA in our RNA-Seq data set. As shown in Figure 3, C and D, we observed significantly higher expression of CCR2 in CD1c^+^ cDC. These findings were accompanied by confirmation of increased XCR1 expression CD141^+^ cDC (Figure 3C) reported in a previous study (25). In addition, we also detected significant changes on CXCR4, CX3CR1, and CCR6 transcriptional levels on circulating CD1c^+^ cDC from RA individuals, but we focused on CCR2, since it has been recently involved in migration of Mo to synovium (37, 38) (Figure 3C). Importantly, FACS analysis indicated that proportions of CD1c^+^ cDC expressing higher surface levels of CCR2 were increased in patients with RA compared with HC (Figure 3D). Interestingly, CCR2^hi^CD1c^+^ cDC also expressed significantly higher levels of CD64 (Figure 3E), indicating that these cells represent a subpopulation migrating from the blood enriched in activated cells. Supporting this possibility, higher proportions of CCR2^+^ cells were also found in Mo and CD1c^+^ cDC infiltrated in the SF from RA subjects (Figure 3F), in which high CD64 expression was previously observed (Figure 1C). Together, our data indicate that CCR2 is an additional marker defining a subpopulation of migratory CD1c^+^ cDC enriched for CD64 expression in the blood and selectively enriched in the SF from patients with RA.

### Inflammatory CD1c^+^ cDC present in SF from patients with RA efficiently activate pathogenic IFN-γ^+^IL-17^+^ T cells.

**Figure 4:**
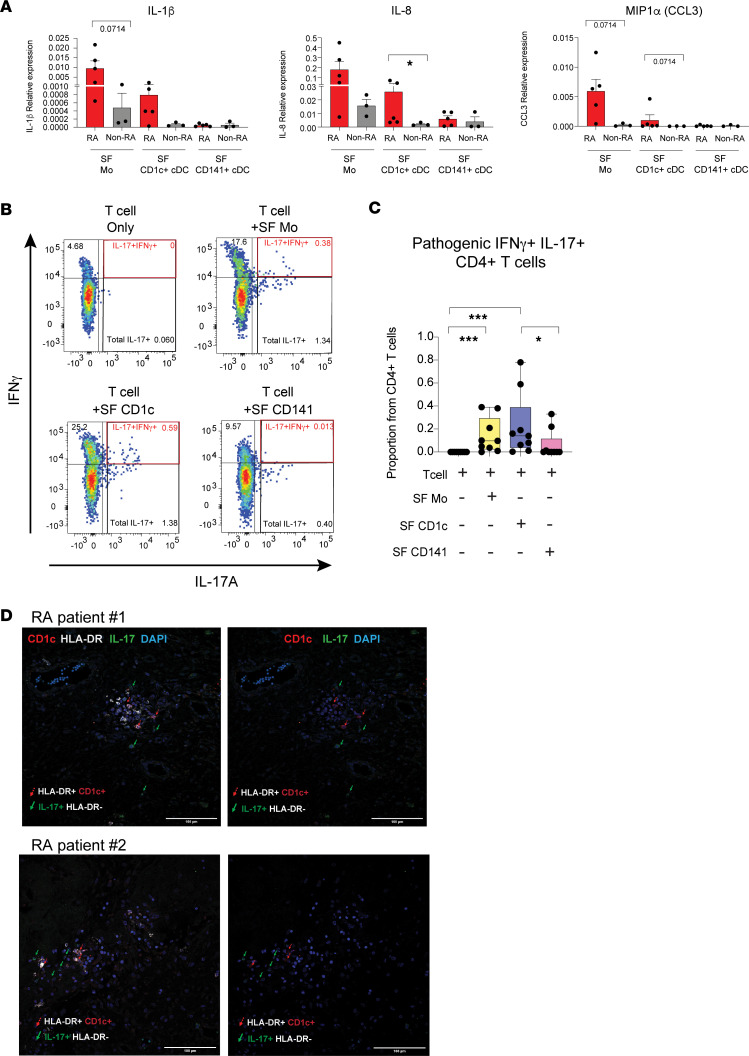
Inflammatory and functional profiles in circulating and synovial CD1c^+^ cDC from patients with RA. (**A**) qPCR analysis of the indicated cytokines relative to β-actin levels in sorted populations from RA (*n* = 5) and CPPD-associated arthropathy (*n* = 3) synovial fluids (SF). (**B**) Representative FACS analysis of IFN-γ versus IL-17a expression on CD4^+^ T cells cultured for 5 days alone or in the presence of Mo, CD1c^+^, or CD141^+^ cDC from the SF of patients with RA. Proportions of total IL17^+^ and pathogenic IL-17a^+^IFN-γ^+^ CD4^+^ T cells are highlighted in black and red gates, respectively. (**C**) Quantification of proportions of pathogenic IL-17a^+^IFN-γ^+^CD4^+^ T cells induced under the conditions defined in **B**, from *n* = 9 HC. Statistical significance was calculated using 2-tailed Wilcoxon matched pairs signed rank test. *P < 0.05; ***P < 0.001. (**D**) Representative confocal microscopy images (original magnification, 40×/1.4-0.75) showing infiltrated HLA-DR^+^CD1c^+^ cells and HLA-DR-IL-17^+^ cells in close proximity in histological sections of synovial membrane from *n* = 6 patients with RA. Images showing coexpression with HLA-DR or without this marker are shown on the left and right, respectively.

Recruitment of CD1c^+^ cDC to the SF suggested that these cells could contribute to the joint inflammation in patients with RA. Supporting this possibility, RNA-Seq and qPCR analyses confirmed that expression of IL-1β, IL-8, and MIP1α tended to be elevated in Mo and CD1c^+^ cDC but not CD141^+^ cells from SF samples of patients with RA compared with alternative synovial myeloid cells from individuals suffering CPPD crystal–associated arthropathy (Figure 4A and Supplemental Figure 6A). At a functional level, from the 3 synovial myeloid cell subsets isolated ex vivo from RA SF samples, Mo and CD1c^+^ cDC were both capable of inducing proliferation of allogeneic CD4^+^ T cells and capable of inducing the activation of a significant portion of these T cells acquiring a Th1-like IFN-γ^+^IL-17^–^ phenotype (Supplemental Figure 6, B and C). In addition, Mo and CD1c^+^ cDC also tended to support proliferation of CD8^+^ T cells and their activation leading to the induction of cytotoxic IFN-γ^+^CD107a^+^ cells in vitro (Supplemental Figure 6D). However, inflammatory patterns of SF CD1c^+^ cDC and Mo from patients with RA were associated with a more efficient induction of IL-17^+^CD4^+^ T cells, compared with CD141^+^ cDC (Figure 4, B and C, and Supplemental Figure 6B). Importantly, higher frequencies of pathogenic IFN-γ^+^IL-17^+^ T cells included within IL-17^+^ cells were more significantly induced by SF CD1c^+^ cDC (Figure 4, B and C). Moreover, we observed CD1c^+^ cDC in close proximity to IL-17^+^ T cells in the synovial membrane from patients with RA presenting advanced joint destruction, some of whom displayed a pathogenic IFN-γ^+^IL-17^+^ phenotype (Figure 4D and Supplemental Figure 6E). Thus, these data indicate that CD1c^+^ cDC may actively contribute to the inflammatory environment and to activating pathogenic Th17 cell responses in the joint of RA individuals.

### RA-like inflammatory profiles and function of CD1c^+^ cDC can be induced upon exposure to intracellular dsDNA.

**Figure 5:**
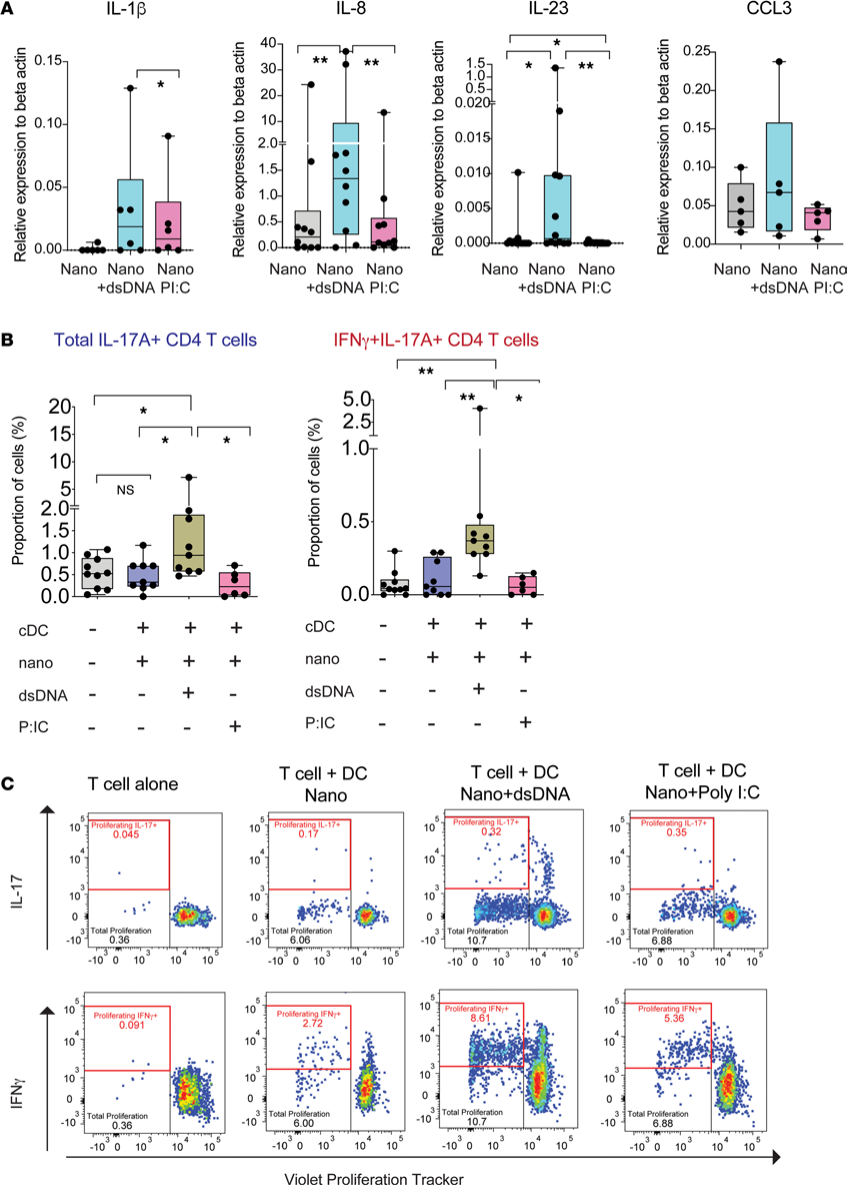
Intracellular dsDNA induces RA-like inflammatory cytokine profile and Th17-stimulating function on CD1c^+^ cDC. (**A**) qPCR analysis of mRNA levels of IL-1β (*n* =6), IL-8 (*n* = 10), IL-23 (*n* = 12), and CCL3 (MIP1α; *n* = 5) relative to β-actin in primary CD1c^+^ cDC after 24 hours of culture in the presence of media and with empty nanoparticles (Nano) or nanoparticles loaded with dsDNA (Nano+dsDNA) or Poly (I:C) (Nano+PI:C). Statistical significance was calculated using a 2-tailed Wilcoxon matched-pairs test. *P < 0.05; **P < 0.01. (**B**) Analysis of frequencies of total IL-17^+^ (left) and IFN-γ^+^IL-17^+^ (right) CD4^+^ T cells cultured for 5–6 days alone or in the presence of allogeneic primary circulating CD1c^+^ cDC prestimulated as previously mentioned at a T cell:DC ratio 1:1 (*n* = 9). Statistical significance was calculated using a 2-tailed Wilcoxon matched-pairs test. *P < 0.05; **P < 0.01. (**C**) Representative FACS dot plots showing levels of Violet Proliferation Tracker versus expression of IL-17 (upper plots) or IFN-γ (lower plots) on CD4^+^ T cells present in the different culture conditions described in **A**.

Our initial transcriptional study showed that innate pathways such as TLR and the inflammasome are altered in CD1c^+^ cDC from patients with RA, and this is associated with increased surface expression of CD64. Previous studies showed the induction of CD64^+^ cells in response to intracellular nucleic acids (39), and this induction might also involve the activation of TLR or inflammasome components. Therefore, we asked whether the inflammatory cytokine profiles observed in CD1c^+^ cDC from RA individuals might be associated with innate sensing of nucleic acids. To this end, we incubated healthy PB cDC (mainly enriched in CD1c^+^ cDC; Supplemental Figure 7A) with nanoparticles loaded with dsDNA or Poly (I:C), to mimic intracellular exposure. CD1c^+^ cDC stimulated with nanoparticles containing dsDNA recapitulated cytokine/TLR signatures from this subset in patients with RA inducing significantly higher levels of IL-1β, IL-8, CCL3, IL-23, and TNF-α transcripts compared with those cells exposed to Poly (I:C), which expressed higher levels of IL-12 and IFN-β (Figure 5A and Supplemental Figure 7B). Functionally, primary CD1c^+^ cDC exposed to dsDNA induced increased proportions of total IL-17^+^CD4^+^ T cells in vitro, and the majority of these cells were characterized by a pathogenic IL-17^+^IFN-γ^+^ phenotype (Figure 5B and Supplemental Figure 7C). Interestingly, induction of IL-17^+^CD4^+^ T cells in the presence of dsDNA-stimulated cDC was not due to proliferation of existing Th17 cells (Figure 5C and Supplemental Figure 7D) or differentiation from naive T cells (Supplemental Figure 7E). Instead, memory CXCR3^+^CD4^+^ T lymphocytes were enriched in cells capable of coexpressing IL-17 and IFN-γ and most likely accounted for the increase of pathogenic Th17 responses in the presence of DNA-primed DC (Supplemental Figure 7F). Together, the data indicate that activation of CD1c^+^ cDC in response to dsDNA induces phenotypical and functional properties that are similar to the inflammatory profiles present in PB and SF CD1c^+^ cDC from patients with RA.

### IgG-dsDNA complexes induce inflammasome activation in CD1c^+^ cDC and RA-like phenotypical characteristics.

**Figure 6:**
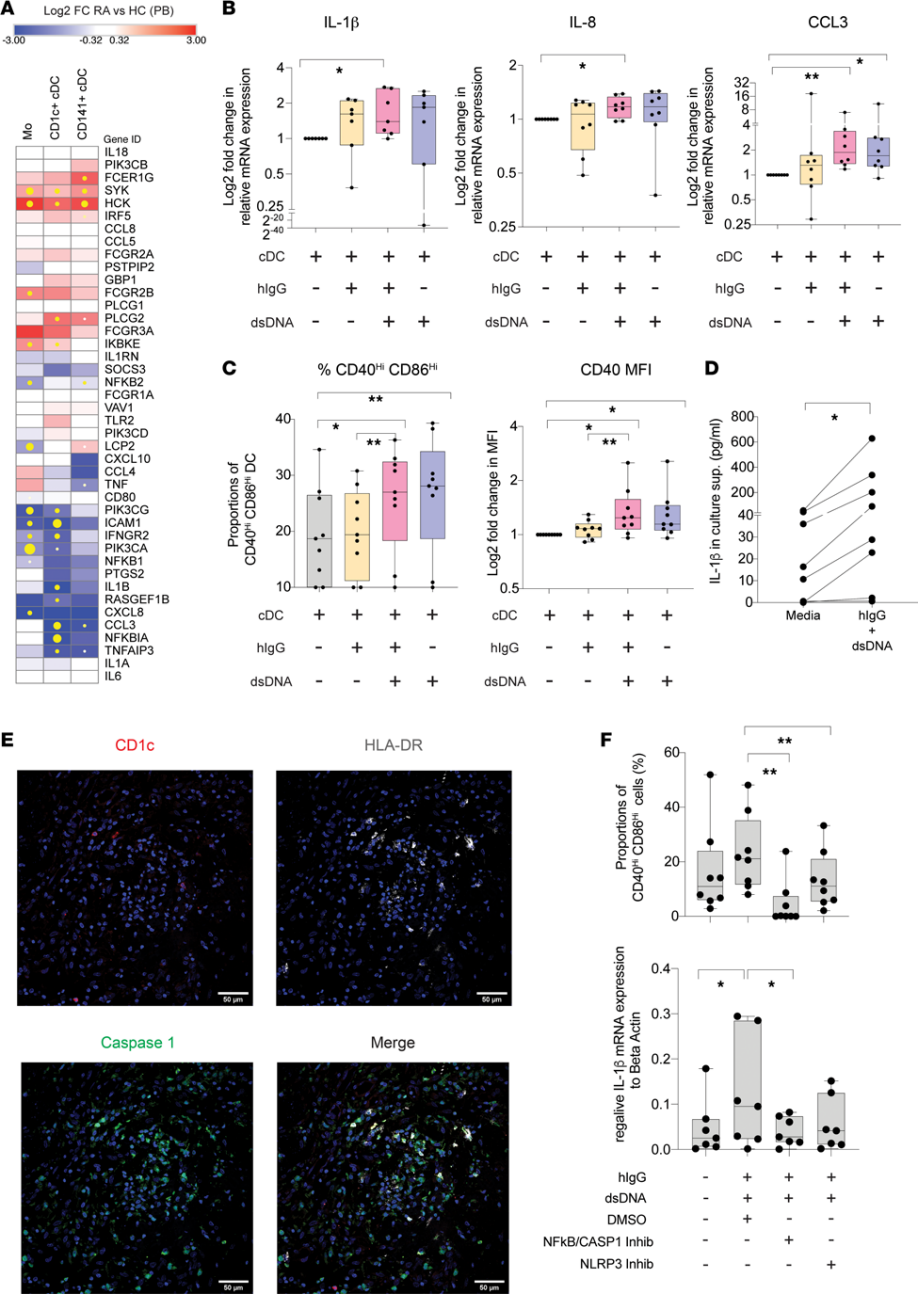
Crosstalk of FcγR and inflammasome in CD1c^+^ cDC in response to dsDNA/IgG complexes. (**A**) Heatmaps reflecting log_2_FC in transcription of 42 genes associated with Fc-receptor signaling on sorted Mo, CD1c^+^, and CD141^+^ cDC from the peripheral blood (PB) of *n* = 4 RA individuals compared with corresponding *n* = 4 healthy controls (HC). Significant DEG are highlighted in yellow (left heatmap, FDR-corrected *P* < 0.05) dots. Size of yellow dots is proportional to significance level. (**B**) qPCR analysis of expression of IL-1β (*n* = 6), IL-8, and CCL3 (*n* = 7) relative to β-actin in circulating cDC cultured for 24 hours in the presence of media or human IgG (hIgG) complexes alone (yellow bars) or in combination with dsDNA (pink bars) or media containing dsDNA (purple bars). *P < 0.05; **P < 0.01. (**C**) Proportions of CD40^+^CD86^hi^ cDC (left) and mean fluorescence intensity (MFI) of CD40 on these cells (right) and analyzed by FACS in the experiments detailed in **B**. *P < 0.05; **P < 0.01. (**D**) ELISA quantification of IL-1β on culture supernatants of CD1c^+^ cDC exposed to media or hIgG-dsDNA complexes for 24 hours. Significance was calculated using a 2-tailed Wilcoxon pairs-matched test. *P < 0.05. (**E**) Representative confocal microscopy image (magnification, 40×/1.4-0.75) analyzing expression of Caspase 1, CD1c, and HLA-DR on histological sections from inflamed synovial membrane from a representative RA patient from *n* = 3 individuals tested. (**F**) Proportions of CD40^hi^CD86^hi^ cDC cultured in media alone or activated with Ig-dsDNA complexes in the presence or either DMSO, a Caspase 1/NF-κB inhibitor, or a NLRP3 inhibitor (*n* = 8 experiments). Statistical significance was calculated using a 2-tailed Wilcoxon test. *P < 0.05; **P < 0.01.

We next investigated the molecular mechanisms connecting inflammatory profiles on CD1c^+^ cDC from patients with RA with intracellular sensing dsDNA, FcγR, and specific pathways driving innate immune activation. Increased levels of CD64 are present on circulating CD1c^+^ cDC in patients with RA (Figure 1C and Supplemental Figure 2D), and expression of molecules associated with FcR-related signaling (SYK, GBP1, PLC, or PI3K) (40) were differentially affected on PB and SF CD1c^+^ cDC from RA subjects (Figure 6A and Supplemental Figure 4A). Therefore, our data suggest that FcR could contribute to the activation of CD1c^+^ cDC from patients with RA. We further analyzed additional PRR pathways that were previously predicted from our RNA-Seq data set (Figure 2B) and that might participate in the activation of CD1c^+^ cDC. The majority of DEG defining the TLR signature of circulating CD1c^+^ cDC from RA individuals included MAPK1, TLR5, MAP2K6, MAP2K4, CD40, TRAF6, TLR10, JUN, and FOS (Supplemental Figure 4B, left). MAPK1 and MAP2K4 also tended to be increased in SF CD1c^+^ cDC compared with the same population from the PB of HC individuals (Supplemental Figure 4B, right). Interestingly, some of these TLR-associated genes have also been involved in TLR2/4/5, TNF-α, and IL-1β–mediated -inflammasome signaling (41–45) and created a unique interconnected signature in this subset from RA subjects (Supplemental Figure 4C). Furthermore, induction of IL-1β, IL-8, and CCL3 has been linked to inflammasome recognition of DNA-containing immunocomplexes, intracellular oxidized DNA, and the expression of CD64 (46–50). Therefore, we assessed whether dsDNA associated with immunoglobulins could trigger the inflammatory cytokine signatures associated with the inflammasome in CD1c^+^ cDC. As shown in Figure 6B, CD1c^+^ cDC stimulated with dsDNA preincubated with human IgGs significantly induced higher mRNAs levels of IL-1β, IL-8, and CCL3 similarly to naked dsDNA. In contrast, IgG alone did not induce significant changes on cytokine expression (Figure 6B). In addition, TLR and inflammasome cytokine signature induced after exposure to dsDNA-IgG complexes and soluble dsDNA was accompanied by higher expression levels of CD40 and CD64 on CD1c^+^ cDC (Figure 6C and Supplemental Figure 8A). Moreover, we observed that CD1c^+^ cDC also secreted higher levels of IL-1β (Figure 6D), suggesting that the activation of the inflammasome might be taking place in CD1c^+^ cDC from patients with RA in vivo. Supporting this possibility, CD1c^+^ cDC infiltrated in the synovial membrane from patients with RA expressed high levels of the inflammasome mediator Caspase 1 (51) (Figure 6E). Moreover, pharmacological inhibition of Caspase 1 and NF-κB prevented the maturation of CD1c^+^ cDC in the presence of the dsDNA-IgG complexes and the transcription of IL-1β (Figure 6F). Interestingly, pharmacological inhibition of Caspase 1 and NF-κB led to a complete abrogation of CD1c^+^ cDC activation and IL-1β expression in response to dsDNA-IgG complexes, while drugs specific for the NLRP3 inflammasome, previously involved in Mo activation in RA (32), led to a partial and less significant effect, suggesting that additional nonredundant inflammasome sensors and NF-κB might be involved in the process. Collectively, our results indicate that innate sensing of dsDNA-IgG complexes might be a mechanism inducing inflammatory signatures and inflammasome activation patterns observed in CD1c^+^ cDC in patients with RA.

### NLRC4 differentially contributes to FcγR-mediated sensing of dsDNA, induction of CD64, and RA inflammatory and functional profiles in CD1c^+^ cDC.

**Figure 7:**
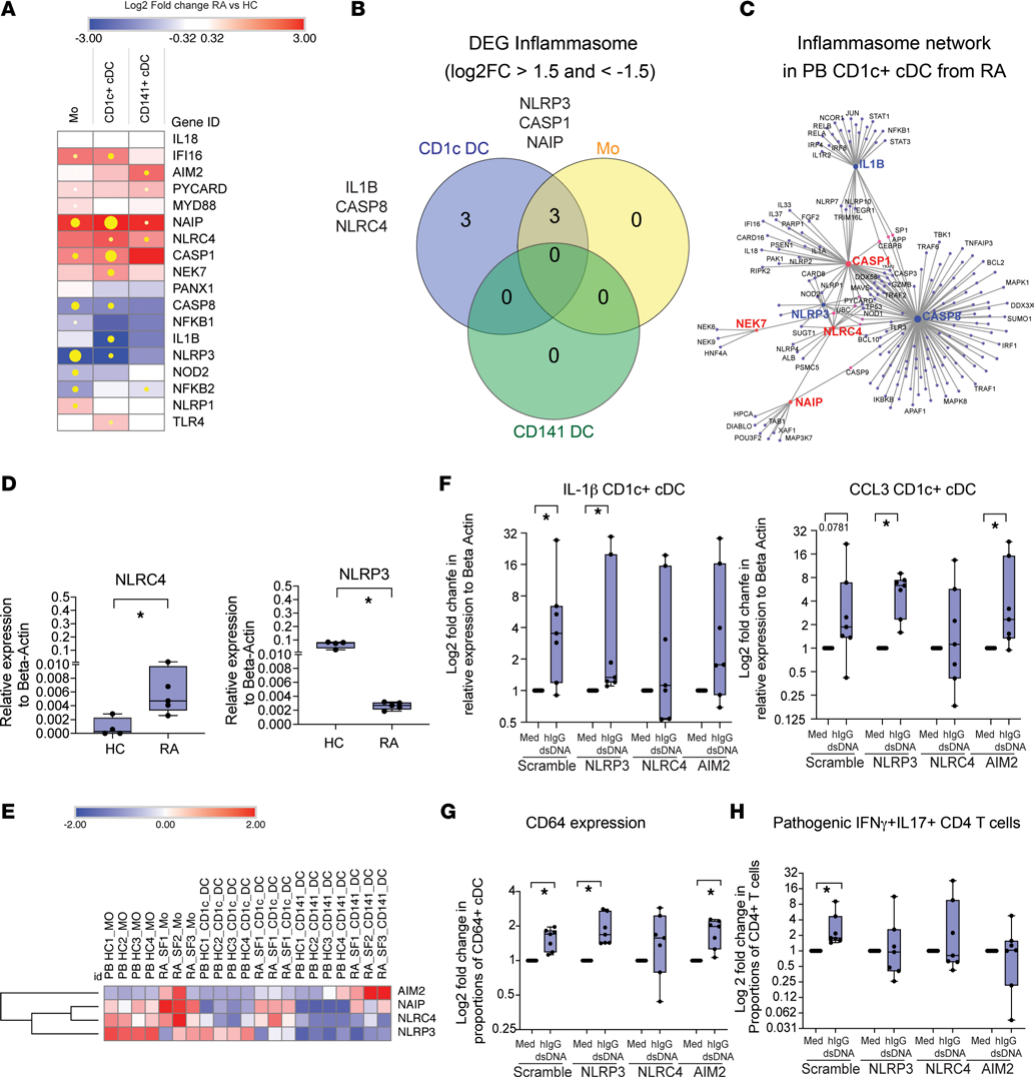
Identification of NLRC4 as the sensor potentially driving pathogenic activation of CD1c^+^ cDC in patients with RA. (**A**) Heatmap representing log_2_FC in transcription of 18 inflammasome genes on each sorted myeloid cell subset from the peripheral blood (PB) from *n* = 4 RA versus *n* = 4 healthy controls (HC) (red, upregulated; blue, downregulated). Size of yellow dots is proportional to statistical significance between PB RA and healthy donors (FDR-corrected *P* < 0.05). (**B**) Venn diagram showing overlap of DEG associated with the inflammasome by IPA in the indicated myeloid cell subsets from the PB of *n* = 4 patients with RA compared with *n* = 4 healthy donors. (**C**) Gene network including significantly upregulated (red) or downregulated (blue) DEG inflammasome genes in PB CD1c^+^ cDC from patients with RA compared with HC (right). Individual (purple) and connected target genes (red) are shown. (**D**) mRNA expression of NLRC4 (left plot) and NLRP3 (right plot) relative to β-actin validated by qPCR in sorted CD1c^+^ cDC from *n* = 5 patients with RA and *n* = 4 HC individuals. Statistical significance was calculated with a 2-tailed Mann Whitney *U* test. *P < 0.05. (**E**) Unsupervised heatmap reflecting normalized expression levels of the indicated inflammasome sensors in Mo, CD1c^+^ cDC, and CD141^+^ cDC from *n* = 3 SF of patients with RA versus the same myeloid subsets from the blood of *n* = 4 HC. (**F**–**H**) Fold change on IL-1β and CCL3 mRNA expression relative to β-actin mRNA levels analyzed by qPCR (**F**), on surface expression of CD64 (**G**), and on functional ability to induce pathogenic IFN-γ^+^IL-17^+^ cells (**H**) in CD1c^+^ cDC nucleofected with indicated siRNAs and cultured in media or in the presence of IgG-dsDNA complexes (*n* = 7 experiments). Statistical significance was calculated using a 2-tailed Wilcoxon matched-pairs test. *P < 0.05.

Finally, we mined our RNA-Seq data set to investigate whether specific sensors might be preferentially driving inflammasome activation in CD1c^+^ cDC in patients with RA. We first compared gene expression of 18 transcripts present on our data set associated with inflammasome activity in Mo, CD1c^+^, and CD141^+^ cDC from patients with RA, regardless our previous significance and log_2_ fold-change cutoff (Figure 7, A and B). Only 8 of these genes passed our original filter, of which 6 transcripts (CASP1, CASP8, NLRC4, NAIP, NLRP3, and IL-1β) were preferentially changed in CD1c^+^ cDC or altered both in these cells and Mo (NLRP3, CASP1, and NAIP). Notably, circulating CD1c^+^ cDC displayed significant differential upregulation and downregulation of NLRC4 and NLRP3 inflammasome sensors, respectively (Figure 7A and Supplemental Figure 8B). In contrast, we did not observe any inflammasome related gene that passed our significance and log_2_ fold-change criteria in PB CD141^+^ cDC from patients with RA, although they showed less significant differential levels of alternative AIM2, PYCARD, or NOD2 and NLRP1 inflammasome sensors (52, 53) (Figure 7B and Supplemental Figure 8B). Interestingly, NLRC4 and NLRP3 have been involved in innate sensing of bacterial products, nucleic acids, and TLR activation (46–50) and created specific differential signaling networks in these cells (Figure 7C). Moreover, higher expression of NLRC4 and lower levels of NLRP3 was validated by qPCR on circulating CD1c^+^ cDC from patients with RA, and cells from the SF presented similar patterns (Figure 7, D and E).

To test whether either NLRC4 or NLRP3 inflammasomes could indeed differentially contribute to the detection of dsDNA-IgG complexes, we performed siRNA-mediated knockdown in primary CD1c^+^ cDC (Supplemental Figure 8C). NLRC4 knockdown in CD1c^+^ cDC most significantly prevented increase of IL-1β and IL-8 mRNA levels compared with baseline in response to dsDNA-IgG complexes, while silencing of NLRP3 had a less significant effect (Figure 7F and Supplemental Figure 8D). Moreover, downregulation of NLRC4 and NLRP3 had opposite effects on CCL3 transcription by primary CD1c^+^ cDC (Figure 7F). In contrast, silencing of alternative inflammasome sensors such as AIM2 did significantly affect the induction of inflammatory cytokines in CD1c^+^ cDC (Figure 7E and Supplemental Figure 8D). Moreover, NLRC4 downregulation by siRNA specifically prevented upregulation of CD64 in response to IgG-dsDNA complexes (Figure 7G). Importantly, silencing of NLRC4 and NLRP3 also reduced the ability of cDC primed with IgG-dsDNA complexes to induce pathogenic IFN-γ^+^IL-17^+^CD4^+^ T cells in vitro, suggesting that activation of the inflammasome is required for such functional specialization (Figure 7H). Unexpectedly, CCR2 was downregulated in response to IgG-dsDNA complexes, and this was also prevented by NLRC4 silencing (Supplemental Figure 8E). Therefore, preferential crosstalk between NLRC4 with FcγRs such as CD64 might drive inflammasome-mediated inflammatory responses to intracellular dsDNA and IgG complexes in CD1c^+^ cDC from patients with RA while interactions between this and other inflammasome sensors are required to acquire Th17-activating functional abilities.

## Discussion

The present translational study compared, in parallel, specific phenotypical, transcriptional, and functional alterations of CD1c^+^ and CD141^+^ cDC, as well as Mo from PB and SF from patients with RA; therefore, it provides an understanding of potentially novel cell subset–specific contributions to chronic inflammation and perpetuation of RA pathology. We have shown that CD1c^+^ cDC, like CD141^+^ cDC, are decreased in the blood of untreated patients with RA and are found in high frequencies in inflamed SF from treated patients, in agreement with other studies (22, 26). In addition, we have shown that treatment more significantly induces the recovery of circulating CD1c^+^ cDC and that this is associated with clinical improvement, supporting that these cells actively contribute to chronicity of RA pathology. In contrast, no significant changes in Mo subsets were observed between our control cohort and patients with RA, and this is not in agreement with previous studies reporting an increase in circulating CD16^+^CD14^hi^ T-Mo. Such differences might be due to the age of our “early initiation” RA cohort, including patients almost 10 years younger than those in cohorts recruited in previous studies (54). This aspect is particularly relevant since higher levels of basal inflammation in older individuals have been associated with increased circulating CD16^+^ Mo at baseline (55, 56) and might explain some of the results obtained with our cohort. Importantly, we have identified expression of CD64 on CD1c^+^ cDC as a marker also for this cell type, and this expression to date had only been described for Mo for patients with RA (36). The expression of this molecule is enriched in a migratory CCR2 subset of CD1c^+^ cDC, which are highly enriched in the SF of patients with RA. Interestingly, CCR2 has been traditionally associated to recruitment of Mo in response to MCP-1 to inflamed sites (37) and has been recently associated with RA activity (38). We now provide evidence that CD1c^+^ cDC might be using similar mechanisms to be recruited to inflamed synovial membrane. However, further studies addressing functionality of this chemokine receptor in cDC are needed. In addition, our RNA-Seq analysis identifies specific transcriptional signatures for different myeloid subsets from patients with RA.

Some of the limitations of our study include the difficulty of obtaining SF samples from untreated patients and healthy subjects, and these limitations might significantly affect levels of inflammation and reduce the resolution of our analysis. In addition, different controls used to compare expression levels in circulating and synovial cDC might also affect our ability to detect differential expression of genes mediating inflammatory responses such as IL-1. On the other hand, due to sample availability, we could not study the impact of circulating and synovial DC on pathogenic CD4^+^ T cell response dynamics on patients at the same stage of RA pathology, and this could have limited our ability to establish more direct associations. In this regard, the synovial membrane sections from patients with advanced disease used in our histology study might have limited our ability to detect higher frequencies of pathogenic Th1/Th17 cells in the tissue. Moreover, differences in gene expression patterns in myeloid cells from SF from patients with RA compared with their circulating homologues or alternative inflammatory conditions, such as CPPD crystal-associated arthritis, might reflect either the selective migration of activated cells or different stages of activation in these 2 distinct immunopathogenic contexts. Therefore, future studies are needed to discriminate between these possibilities. Despite these limitations, we have identified specific transcriptional FcR, TLR, and inflammasome signatures differentially altered in CD1c^+^ cDC from both PB and SF. Interestingly, the ability of SF CD1c^+^ cDC to induce secretion of IL-17 by CD4^+^ T cells has been previously reported (26), although it was not compared in parallel with Mo and CD141^+^ cDC.

In the present study, we describe for the first time to our knowledge that SF CD1c^+^ cDC from patients with RA preferentially display a high capability to activate pathogenic IL-17^+^IFN-γ^+^CD4^+^ T cells involved in autoimmune diseases (8–10). This is particularly relevant, given the recent evidence implication of these cells in RA pathology in patients (57) and in animal models (58). In fact, we demonstrate that CD141^+^ cDC do not efficiently support Th17 responses, but future studies should address whether both cDC subsets functionally cooperate during disease pathogenesis. While recent data on the blockade of IL-17 did not yield results as promising in clinical trials as initially expected (59), it is important to highlight that Th1/Th17 cells have been shown to produce additional inflammatory cytokines such as GM-CSF (60), and it has been recently shown that the blockade of the receptor for this cytokine with mavrilimumab has an effect on improving clinical parameters in patients with RA (61). Moreover, we report enhanced abilities of dsDNA-stimulated CD1c^+^ cDC differentiation of CXCR3^+^CD4^+^ T cells into pathogenic Th1/Th17 T cells, supporting their capacity to increase memory T cells plasticity in patients with RA (62). Moreover, we provide evidence that dsDNA-IgG complexes might act as DAMPs and trigger pathogenic activation of CD1c^+^ cDC in a Caspase 1/NK-κB–dependent manner. This possibility is supported by previous studies reporting extracellular dsDNA from neutrophils through NETosis in patients with RA (63). In addition to CD4^+^ T cells, our data suggest that CD1c^+^ cDC and Mo might be able to induce activation of cytotoxic CD107a^+^IFN-γ^+^CD8^+^ T cells. It has been recently described that activated granzyme K^+^CD8^+^ T cells in the inflamed synovium might contribute to RA pathology (64). However, further studies are required to determine whether CD8^+^ T cells activated in the presence of CD1c^+^ cDC can specifically acquire the phenotypical and functional properties of this particular granzyme K^+^CD8^+^ T cell subset. Therefore, we provide a functional mechanism whereby CD1c^+^ cDC might contribute to the perpetuation of chronic inflammation and RA pathology.

At the molecular level, we have identified NLRC4 as an inflammasome sensor differentially upregulated in CD1c^+^ cDC from patients with RA, and this sensor seems to be nonredundantly involved in the detection of intracellular dsDNA. In contrast, downregulated expression of NLRP3 in CD1c^+^ cDC from patients with RA suggests a limited role of this sensor-driving activation of this particular subset, in agreement with the presented siRNA-knockdown results. However, NLRP3 can recognize a variety of DAMPs, including nucleic acids (48–50), and can mediate activation of Mo after FcγR stimulation with IgG and antigen immunocomplexes (39, 65, 66); this supports a relevant role of this sensor in other myeloid subsets such as Mo. On the other hand, NLRC4 inflammasome can be activated in response to bacterial components (67, 68) and innate antiviral responses (69, 70). Importantly, the expression of NLRC4 is also associated with autoimmune disorders in the nervous system (71, 72) or the skin (73). Interestingly, there was no information on the role of this molecule on dsDNA sensing or association with FcRs in specific innate immune cell subsets or in the context of RA. Remarkably, the expression of NLRC4 and NLRP3 seems to stablish a balance that tightly regulates CCL3 in CD1c^+^ cDC in response to dsDNA in a subset-specific manner. Therefore, these 2 molecules might represent 2 nonredundant therapeutic targets for RA. Supporting this interpretation, mutation on NLRC4 might affect predisposition to develop inflammatory diseases such as RA (74). While recent studies in animal models suggest that NLRP3 inhibitors might be useful for treating RA (33), our data suggest that combined targeting of CD64 and both NLRC4 and NLRP3 inflammasomes might be a useful synergistic strategy to reduce aberrant inflammatory responses in CD1c^+^ cDC, as well as other myeloid subsets, and prevent RA progression. A standing question is whether NLRC4 and NLRP3 might act in conjunction or independently to TLR stimulation. Our data are consistent with high levels of TLR activation on CD1c^+^ cDC. However, the implication of TLRs involved, components of the signaling cascade, and the interaction with the inflammasome has not been addressed in our study. Thus, more studies are required to fully understand these aspects and the nucleic acid–sensing pathways and their potential as therapeutic targets in human CD1c^+^ cDC and Mo. Together, our study provides relevant information about the contribution of CD1c^+^ cDC to cellular networks participating in RA pathogenesis and identifies crosstalk between the NLRC4 inflammasome and CD64 as potential future therapeutic targets for CD1c^+^ cDC in patients with RA.

## Methods

### Study participants.

PB mononuclear cells (PBMC) were obtained from patients included in Princesa Early Arthritis Register Longitudinal (PEARL) study (75). Twenty-five untreated individuals (15 fulfilling RA criteria and 10 undifferentiated arthritis [UA]) were studied. Their baseline features are shown in Supplemental Table 1. For comparison purposes, PBMC from 17 HC were isolated from buffy coat samples obtained from the Centro de Transfusiones Comunidad de Madrid, Madrid, Spain and from 13 age- and sex-matched individuals recruited from Hospital de la Princesa sample repository (Supplemental Table 1). Longitudinal studies with additional patients with RA were performed after 1 and 2 years in treatment (Supplemental Table 3). Mononuclear cells obtained from SF samples drained for therapeutic or diagnostic procedures from 16 individuals presenting different rheumatic disorders — including RA, CPPD crystal–associated arthropathy, and spondyloarthritis — who were receiving different treatment regimens (see Supplemental Table 2) were used for additional phenotypical and transcriptional validation studies.

### Flow cytometry phenotypical analysis and cell sorting.

Ex vivo and cultured PBMC were stained with APC-H7 (Tonbo Biosciences) or Brilliant Violet 405 (Molecular Probes) viability dye in the presence of different panels of monoclonal antibodies against lineage (Lin) (CD3 clone HIT3a, CD19 clone SJ25C1, CD20 clone 2H7, and CD56 clone 5.1H11), CD14 clone M5E2, CD16 clone 3G8, CD40 clone 5C3, CD86 clone IT2.2, ILT4 clone 42D1, HLA-DR clone L243, CD11c clone 3.9, CD1c clone L161, CD141 clone M80, CD64 clone 10.1, and PDL1 clone 29E.2A3 (BioLegend). Samples were analyzed on a Fortessa cytometer (BD Biosciences) at Centro Nacional de Investigaciones Cardiovasculares (CNIC, Madrid, Spain). Analysis of individual and multiparametric flow cytometry data was performed using FlowJo software (Tree Star Inc.).

For the transcriptional studies, viable human Lin^–^CD14^–^CD11c^+^HLADR^+^CD1c^+^ cDC, CD14^–^CD11c^+^HLADR^+^CD141^+^ cDC, and total CD14^+^ Mo were sorted using a FACS Aria II sorter (BD Biosciences) from either PBMC from *n* = 4 untreated patients with RA and *n* = 4 HC, or SF from *n* = 3 individuals with RA and *n* = 3 suffering mechanic CPPD crystal–associated arthritis.

### Gene expression analysis by RNA-Seq and computational data analysis.

RNA was isolated from sorted Mo, CD1c^+^, and CD141^+^ cDC populations from PB from *n* = 4 untreated patients with RA and *n* = 4 HC, or from the SF of *n* = 3 RA and *n* = 3 CPPD crystal–associated arthritis patients using the RNeasy Micro Kit (Qiagen). Quality and integrity of each RNA sample was checked using a Bioanalyzer 2100 instrument (Agilent) before proceeding to RNA-Seq protocol. Subsequently, selected RNAs from cDC were used to amplify the cDNA using the SMART-Seq v4 Ultra Low Input RNA Kit (Clontech-Takara). In total, 1 ng of amplified cDNA was used to generate barcoded libraries using the Nextera XT DNA library preparation kit (Illumina). The size of the libraries was checked using the Agilent 2100 Bioanalyzer High Sensitivity DNA chip, and their concentration was determined using the Qubit fluorometer (Thermo Fisher Scientific).

RNA from circulating Mo was processed as follows: 200 ng of total RNA were used to generate barcoded RNA-Seq libraries using the NEBNext Ultra II Directional RNA Library Prep Kit (New England Biolabs Inc). Libraries were sequenced on a HiSeq 2500 (Illumina) and on a HiSeq 4000 and processed with RTA v1.18.66.3. FastQ files for each sample were obtained using bcl2fastq v2.20.0.422 software (Illumina).

Sequencing reads were aligned to the human reference transcriptome (GRCh38 v91) and quantified with RSem v1.3.1 (76). Raw counts were normalized with transcripts per million (TPM) and trimmed Mean of M-value (TMM) methods, transformed into log_2_ expression (log_2_[rawcount + 1]), and compared with calculated fold-change and corrected *P* value. Only those genes expressed with at least 1 count in a number of samples equal to the number of replicate samples of the condition with less replicates were taken into account. Gene expression changes were considered significant if associated to Benjamini and Hochberg adjusted *P* <0.05 and a log_2_ fold change in gene expression greater than 1.5 and lower than –1.5.

Heatmaps were generated with Morpheus online tool from Broad Institute (https://software.broadinstitute.org/morpheus). Finally, pathway analysis and visualization of gene networks for each DEG list was performed using either IPA (Qiagen), DAVID Bioinformatics Resources 6.8, Metascape (http://metascape.org/gp/index.html#/main/step1), or NetworkAnalyst (77) Software.

### Data availability.

RNA-Seq data from the study have been deposited on the public Gene Expression Omnibus (GEO) repository (accession no. GSE157047; https://www.ncbi.nlm.nih.gov/geo/query/acc.cgi?acc=GSE157047)

### Validation of gene expression by qPCR.

RNA was isolated from sorted PB or SF myeloid subsets using RNeasy Micro Kit (Qiagen) according to manufacturer instructions. cDNA was synthesized using the reverse transcription kit (Promega), and gene expression was analyzed by semiquantitative PCR using the SYBR Green assay GoTaq qPCR Master Mix (Promega) with standardized primers (Metabion) on a StepOne Real-Time PCR system (Applied Biosystems). Relative gene expression was normalized to β-actin detection.

### siRNA-mediated gene knockdown.

Gene knockdown of NLRP3, NLRC4, and AIM2 was performed by nucleofection of fresh primary cDC (Amaxa4D-Nucleofector, Lonza) with specific siRNAs (SMART-pool, Horizon Discovery) or irrelevant scramble siRNA according to manufacturer instructions. siRNA-mediated knockdown was analyzed after 24 hours by qPCR.

### Isolation of primary DC and T cells.

Total cDC enriched for CD1c^+^ cDC were purified from total PBMC suspensions by immunomagnetic enrichment (purity > 90%) using the Human Myeloid DC Enrichment Kit (STEMCELL). Total T cells and CD4^+^ T cells were isolated using the Untouched Human T cell and CD4^+^ T cell kits (Invitrogen), leading to a cell suspension of purity > 95%. For some experiments, circulating naive and CXCR3^+^ memory CD4^+^ T cells were isolated from previously purified total CD4^+^ lymphocyte fractions using a manual EasySep Human Naive CD4^+^ T Cell Isolation Kit (Stemcell Technologies) or using PE-labeled anti-CXCR3 mAb (BioLegend) plus anti-PE microbeads (Miltenyi Biotec) and the AutoMACS cell sorter (Miltenyi Biotec), respectively. For functional assays with cells present in the SF, total CD14^+^ Mo, CD1c^+^ and CD141^+^ cDC were sorted by following the flow cytometry strategy previously described in the Methods section.

### In vitro stimulation of DC and functional assays.

Primary cDC were cultured for 24 hours in the presence of polymeric nano-particles (TransIT-X2, Mirus Bio) alone or preloaded with either 5 μg of Salmon Sperm dsDNA (Invitrogen) and 5 μg Poly I:C (MilliporeSigma) according to manufacturer instructions. In other set of experiments, primary cDC were incubated with human IgG alone (MilliporeSigma) or in complexes with the mentioned Salmon sperm dsDNA. Expression of CD86 (clone IT2.2, BIolegend) and CD40 (clone 5C3, Biolegend) was analyzed by flow cytometry. In some experiments, levels of CD64 (clone 10.1, Biolegend) and CCR2 (clone K036C3, Biolegend) were also assessed. Subsequently, DC were cocultured with allogeneic total, naive, or CXCR3^+^ CD4^+^ T cells for 5 days at a 1:1 ratio in media supplemented with 25 IU/mL IL-2 (Peprotech). In some experiments, cDC were previously nucleofected with siRNAs targeting candidate intracellular sensors. Intracellular expression of IFN-γ (clone 4S.B3, BD Pharmigen) and IL-17 (clone BL168, Biolegend) on cultured T cells was analyzed by flow cytometry at the end of the assay at day 5 after restimulation with 0.25 μg/mL PMA (MilliporeSigma) and Ionomycin (MilliporeSigma) for 1 hour and cultured for 4 hours in the presence of 0.5 μg/mL brefeldin A (MilliporeSigma) and 0.005 mM monensin. In some experiments, T cells were prelabeled with Violet CellTrace proliferation tracker (Invitrogen). In some experiments, cDC or MDDC were stimulated with 5 μg of Salmon Sperm dsDNA (Invitrogen) and preincubated for 3 hours at 37°C in the presence of media or 100μg/mL human IgG to facilitate complex formation. After 24 hours, cytokine expression was analyzed by qPCR.

### Histological analysis of synovial tissue.

Synovial membrane sections were paraffin embedded and segmented in fragments of 3–5 μm of thickness in a Leica microtome. Tissue section deparaffinization, hydration, and target retrival were performed with a PT-LINK (Dako) before staining. For staining of CD1c^+^ cDC, we used a mouse anti–human CD1c clone OTI2F4 and rabbit anti–human HLA-DR clone EPR6148 primary antibodies (Abcam). Expression of IL-17 or Caspase 1 was analyzed with either a goat anti–IL-17 or a goat anti–Caspase 1 primary antibodies (R&D), and expression of IFN-γ was evaluated using a rabbit anti–IFN-γ clone EPR 21704 antibody (Abcam). Secondary antibodies used included a donkey anti–rabbit Alexa Fluor 488, donkey anti–mouse Alexa Fluor 647, and donkey anti–goat Alexa Fluor 568 (ThermoFisher Scientific) as secondary antibodies. Images were taken with a Leica TCS SP5 confocal and processed with the LAS AF software. Images were further processed using the ImageJ software (NIH).

### Statistics.

Gene expression changes were considered significant following the criteria described above. Significance of differences between the cells from different patient cohorts or within the same patient cohort were assessed using Mann Whitney *U* or Wilcoxon matched-pairs signed-rank tests. Multiple-comparison correction using a Kruskal-Wallis test with post hoc Dunn’s test, the Bonferroni correction, or FDR methods was applied when appropriate. *P* values lower than 0.05 were considered significant.

### Study approval.

All subjects gave written informed consent, and the study was approved by the IRB of Hospital Universitario de La Princesa (register no. 3515) and following the Declaration or Helsinki.

## Author contributions

EMG, IGA, FSM, SC, CDA, MCM, and ATM developed the research idea and study concept, designed the study, and wrote the manuscript; EMG and IGA supervised the study; CDA, MCM, and ATM designed and conducted most experiments and equally contributed to the study; OP and IT participated in the functional analysis of si-RNA–treated DC; IGA, SC, and FSM provided PB and SF samples from study patient cohorts; ATM, SC, and IGA provided information of clinical parameters during the study; EVDL provided bioinformatic support for computational RNA-Seq data analyses and supervised statistical analysis; ABF, RMV, and AD performed and collaborated in the RNA-Seq experiments; EMG and MCM performed pathway analysis of transcriptional data; CDA, MCM, and ISC performed phenotypical analysis and sorting of samples; DCF participated in flow cytometry analysis; AR provided access and support for sample cell sorting; RL and GHB provided RA synovial membrane specimens for histological analyses; and HDLF and FSM provided qPCR reagents and cell samples from healthy donors and participated in data discussion.

## Supplementary Material

Supplemental data

Supplemental table 5

## Version 1 10/04/2022

In-Press Preview

## Version 2 11/22/2022

Electronic publication
