# Supplementary material for: NLRC4-mediated activation of CD1c+ DC contributes to perpetuation of synovitis in rheumatoid arthritis
Source: JCI Insight. 2022 Nov 22;7(22):e152886. doi: 10.1172/jci.insight.152886 (PMC9746818; doi:10.1172/jci.insight.152886)
Supplement: Supplemental data [file jciinsight-7-152886-s077.pdf]

SUPPLEMENTAL MATERIAL TITLES AND LEGENDS

Supplemental Figure 1. Frequencies of different circulating myeloid cell subsets in the blood from RA patients at baseline and after treatment initiation. (A): Flow cytometry gating strategy subdividing viable leukocytes into CD14^{+} Monocytes and Lineage (Lin; CD3, CD19, CD20, CD56) negative CD14^{-} HLA-DR^{+} CD11c^{+} conventional (cDC) and CD11c^{-} CD123^{+} plasmacytoid (pDC) dendritic cells. CD14^{+} Mo and cDC subsets were further defined on the basis of the expression of CD16 and CD1c vs CD141, respectively. (B): Box and whisker plots representing frequencies of Lin- HLA-DR^{+} CD11c^{+} CD1c^{+} cDCs and cDC141^{+} cDCs, Lin^{-} HLA-DR^{+} CD11c^{-} CD123^{+} pDCs, CD16^{-}CD14^{Hi} classical, CD16^{+}CD14^{+} transitional and non-classical CD16^{Hi}CD14^{Lo} Mo subsets in live mononuclear cells from peripheral blood from healthy controls (HC; blue, n=28) and untreated RA patients (magenta, n=31) obtained by flow cytometry. Synovial Fluid (SF) samples from treated RA patients (grey, n=13) were also included for comparison purposes. Significance was calculated using a Kruskal Wallis test and corrected for multiple comparison with a Dunn´s test. (C): Spearman correlation between age and proportions of CD1c^{+} cDCs in the blood from HC (upper plot) and RA (lower plot) individuals. Data are shown as dot plots and their fitted linear prediction with 95% confidence interval (grey shadow) estimated using the *two-way* command of Stata with the *lfitci* option. P and r values are shown on each plot. (D): Log2 fold change in proportions of CD141c^{+} cDCs, classical, transitional and non-classical Mo in blood samples from n=14 RA patients collected at the first visit (untreated baseline) and after 1 or 2 years after treatment duration.

Supplemental Figure 2. Phenotypical characteristics of circulating myeloid cell subsets in the blood from RA patients. (A-C): Box and Whiskers plots representing proportions of CD86^{+}, CD40^{+}, CD64^{+} and CD16^{+} cells on Lin^{-} HLADR^{+} CD11c^{-} CD123^{+} pDCs (A), Mo subsets (B) or only CD86^{+} cells for CD1c^{+} and CD141^{+} (C) cDCs from the blood of n=28 healthy control (HC; blue) and n=31 RA (magenta) individuals and in the synovial fluid (SF; grey) from RA patients. Statistical significance was calculated using a Kruskal Wallis test and corrected for multiple comparisons with a Dunn´s test. (D): Spearman correlation between DAS28-ESR of proportions of circulating CD1c^{+} cDCs and percentage of CD64^{+} cells in this population in n=30 RA patients. R and p values considering all patients (black), individuals with DAS28-ESR higher (red) or lower (blue) than 4 are shown.

Supplemental Figure 3. Visualization of differential transcriptional patterns of Mo and cDC subsets from the blood of RA patients. Principal Component analysis (PCA) of transcriptional patterns from Mo (left), CD1c^{+} cDCs (middle) and CD141^{+} cDCs (right) of n=4 RA patients (blue) compared with n=4 healthy control individuals (red). Percentages of DEG defining each PC are indicated on the axes.

Supplemental Figure 4. Differential transcriptional expression of TLR associated genes in cDC subsets and Mo from RA patients. (A-B): Heatmaps reflecting normalized expression of genes associated to FcR signaling (A) and TLR related genes (B) on circulating (left) and synovial fluid (SF, right) Mo, CD1c^{+} and CD141^{+} cDCs from RA (n=4 PB: n=3 SF patient samples) compared to circulating cells from n= 4 healthy controls (HC). Data from individual patients are shown (upregulated genes, red; downregulated genes blue). (C): Gene network including significantly upregulated (red) or downregulated (blue) DEG TLR-associated genes in PB CD1c^{+} cDCs from RA patients compared to HC (right). Individual (violet) and connected target genes (pink) are shown.

Supplemental Figure 5. Overlap of differentially expressed genes in synovial fluid myeloid cell subsets from RA and CPPD patients. Venn´s diagram of DEG (nominal $p<0.05$) detected in Mo, CD1c^{+} and CD141^{+} cDC from the synovial fluid (SF) from n=3 RA patients compared to n=3 Calcium Pyrophosphate Crystal Deposition (CPPD)-associated arthopathy patients.

Supplemental Figure 6. Functional characteristics of DC subsets from synovial fluid from RA patients. (A): Heatmaps representing normalized expression (left) or log2 fold change in transcription (right) of genes involved in inflammatory cytokine pathways (12 genes, lower right) in SF myeloid subsets from RA or CPPD patients. (B): Box and Whiskers plots representing raw proportions of total IL-17^{+} CD4^{+} T cells detected after 5 days in culture in the presence of allogeneic Mo (yellow), CD1c^{+} (blue) and CD141^{+} (pink) cDCs sorted from the synovial fluid (SF) of n=9 RA patients. Triplicates of control culture conditions are shown.

Statistical significance was calculated using a non-parametric two tailed Wilcoxon matched pairs test (*$p<0.05$; ***$p<0.001$). (C-D): Proportions of IFNγ^{+} IL-17^{-} Th1-like CD4^{+} T cells (C), CD107a^{+} CD8^{+} T cells (D) and proportions of total and IFNγ^{+} proliferating CD4^{+} (C, bottom) and CD8^{+} T cells (D, bottom) (defined as violet tracker low cells) in the in vitro conditions detailed in (B). Statistical significance in (C-D) was calculated using a non-parametric two tailed Wilcoxon matched pairs test (*$p<0.05$; ***$p<0.001$). (E): Representative confocal microscopy images (magnification 40X) showing infiltrated CD1c^{+} cells and IL-17^{+} and IFNγ+ cells in histological sections of synovial membrane from a RA patient from n=5 tested individuals. Pathogenic IL-17^{+} IFNγ^{+} (Th1/Th17) cells are highlighted with arrows. Merged and individual fluorescent marker combined with DAPI are shown.

Supplemental Figure 7. Acquisition of Th17-activating function by circulating CD1c^{+} cDCs after intracellular stimulation with nucleic acids in vitro. (A): Representative flow cytometry analysis of HLA-DR vs Lineage (CD3+CD19+CD20+CD56+CD14) markers (upper left plot) and HLA-DR vs CD11c and CD1c vs CD141 on gated Lin^{-} HLA-DR^{+} cells from cDCs immunomagnetically purified from the blood of healthy donors used for functional assays. (B): RT-qPCR analysis of expression of TNFα, IL-6, IL-12 and IFNβ in primary cDCs from blood stimulated with nanoparticles alone (grey) or loaded with either dsDNA (blue) or Poly I:C (pink). Statistical significance was calculated using a two-tailed Mann Whitney test (*$p<0.05$; **$p<0.01$). (C): Representative FACS analysis of IFNγ vs IL-17a expression on CD4^{+} T cells cultured for 5 days alone or in the presence CD1c^{+} cDCs stimulated in conditions defined in (B). (D): Mean Fluorescence Intensity of Violet proliferation tracker on indicated CD4^{+} T cells subsets after 5 days in culture in conditions specified in (B), left. Proportions of proliferating IL17^{+} and IFNγ^{+} cells are also shown, right. Data correspond to n=4 experiments (E-F): Proportions of IL-17^{+} (E) or IL-17^{+} IFNγ^{+} (F) T cells derived from total versus naïve CD4^{+} T cells (E; n =6) or from total CD4^{+} T cells versus CXCR3^{+} Th1-enriched CD4^{+}T cells (F; n=8) after 5 days of culture in the presence of allogeneic cDCs treated with nanoparticles alone or loaded with dsDNA or Poly (I:C). Statistical significance was calculated using a two tailed Wilcoxon matched-pairs test. *$p<0.05$.

Supplemental Figure 8. Inflammasome related genes and impact of siRNA-mediated knock down of NOD-like receptor sensors regulating activation of CD1c^{+} cDCs. (A): Proportion of CD64^{+} cDCs detected by FACS after culture in the presence of media alone (Med) or dsDNA-IgG complexes for 16h. Statistical significance was calculated using a two-tailed Wilcoxon test. (B): Heatmaps reflecting Log2 fold change of expression of genes associated to the inflammasome in Mo, CD1c^{+} and CD141^{+} cDCs from the blood of n=4 (left) and the synovial fluid of n=3 (right) RA patients vs circulating cells from n=4 healthy individuals (HC) (upregulated genes, red; downregulated genes, blue). (C): RT-qPCR analysis of expression of NLRP3, NLRC4 and AIM2 relative to β-Actin analyzed in primary cDCs nucleofected with specific siRNAs and normalized to cells treated with irrelevant scramble siRNA. (D-E): Fold change on mRNA expression of the indicated cytokines relative to β-Actin expression analyzed by RT-qPCR (D) and on surface levels of CCR2 (E) in circulating CD1c^{+} cDC nucleofected with either scramble siRNA or specific siRNAs and cultured for 24h in the presence of media alone or with human IgG (hIgG) and dsDNA complexes. Data correspond to n=6 experiments. Statistical significance highlighted in B and C was calculated using a two-tailed Wilcoxon test; *$p<0.05$.

Supplemental Table 1. Clinical characteristics of untreated early arthritis patients and matched healthy controls recruited for PBMC collection and our studies.

Supplemental Table 2. Clinical characteristics of patients providing synovial fluid samples used for flow cytometry and RNA-seq studies.

Supplemental Table 3. Clinical and treatment information of RA Patient samples used for longitudinal analysis.

Supplemental Table 4. Differentially expressed genes detected by RNA-seq in CD1c+ cDC from peripheral blood of n=4 rheumatoid arthritis (RA) versus n=4 healthy controls (HC) considering Log2FC >1.5 and <-1.5.

Supplemental Table 5. Cannonical Pathway Prediction by Ingenuity Pathway analysis of different myeloid subsets from DEG of RA patients compared to healthy donors considering 1.5 and -1.5 Log2 FC as a criteria.

Supplemental Table 6. Differentially expressed genes detected by RNA-seq in CD1c+ cDCs from synovial fluid of n=3 rheumatoid arthritis (RA) versus n=3 Calcium Pyrophosphate Deposition (CPPD) Crystal-associated arthritis patients.

Supplemental Figure 1

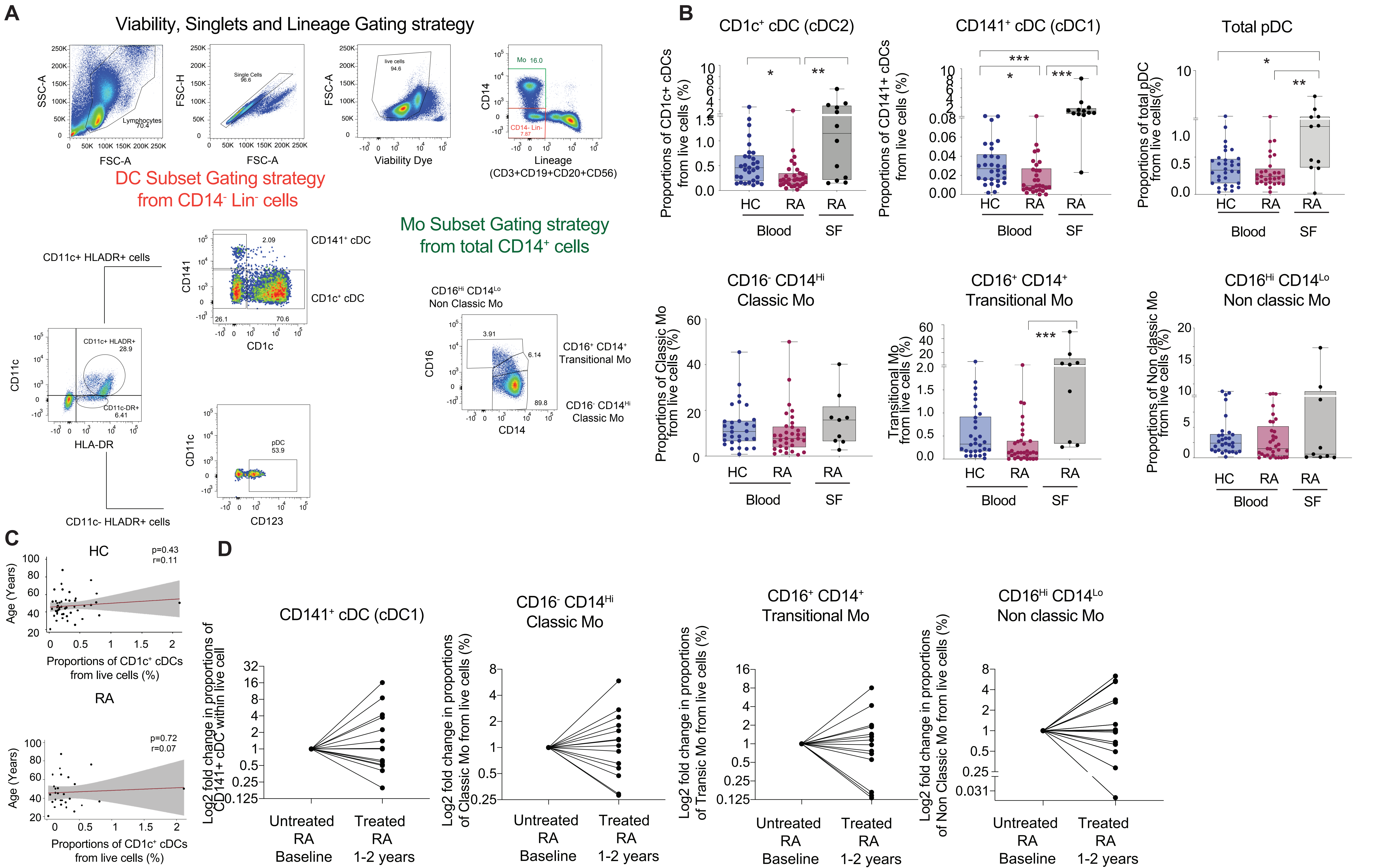

Supplemental Figure 2

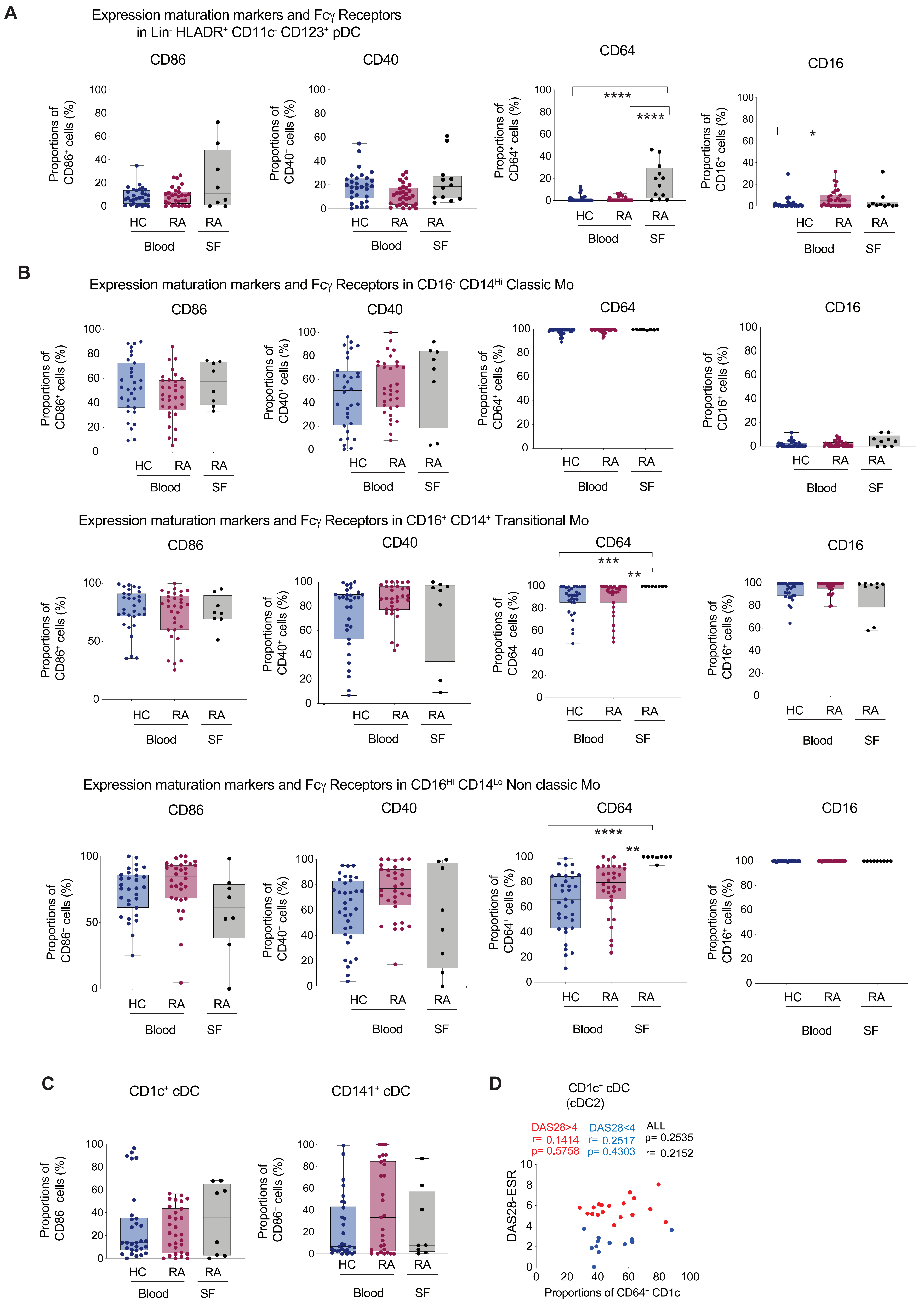

Supplemental Figure 3

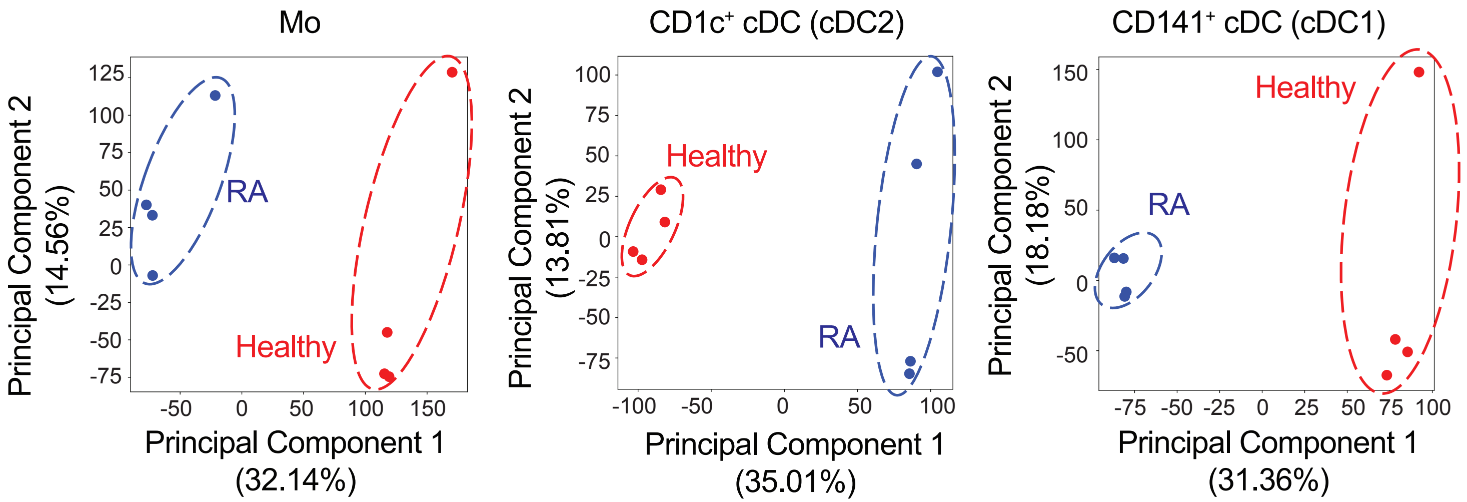

Supplemental Figure 4

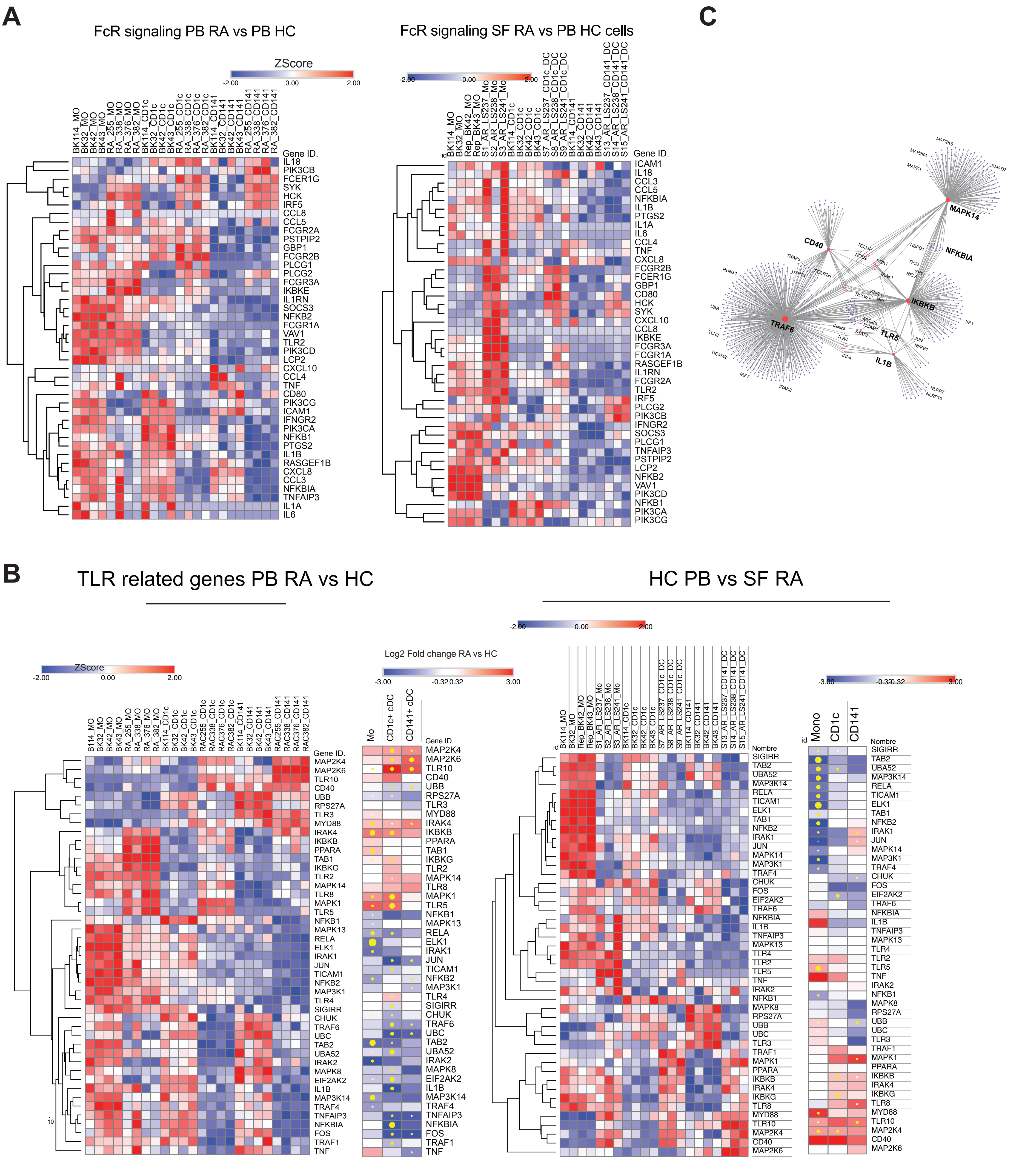

Supplemental Figure 5

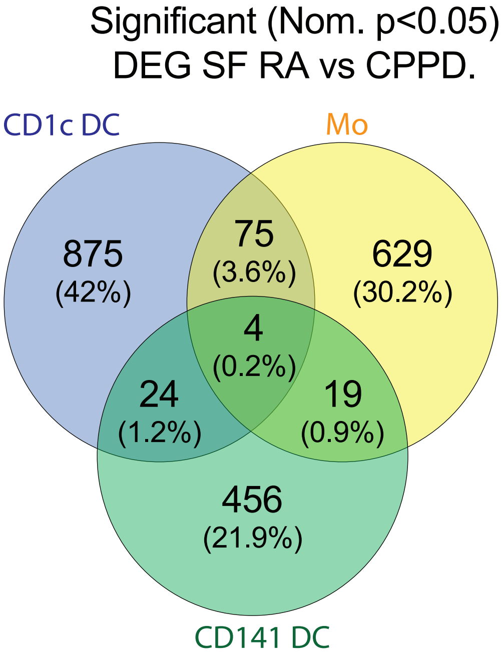

Supplemental Figure 6

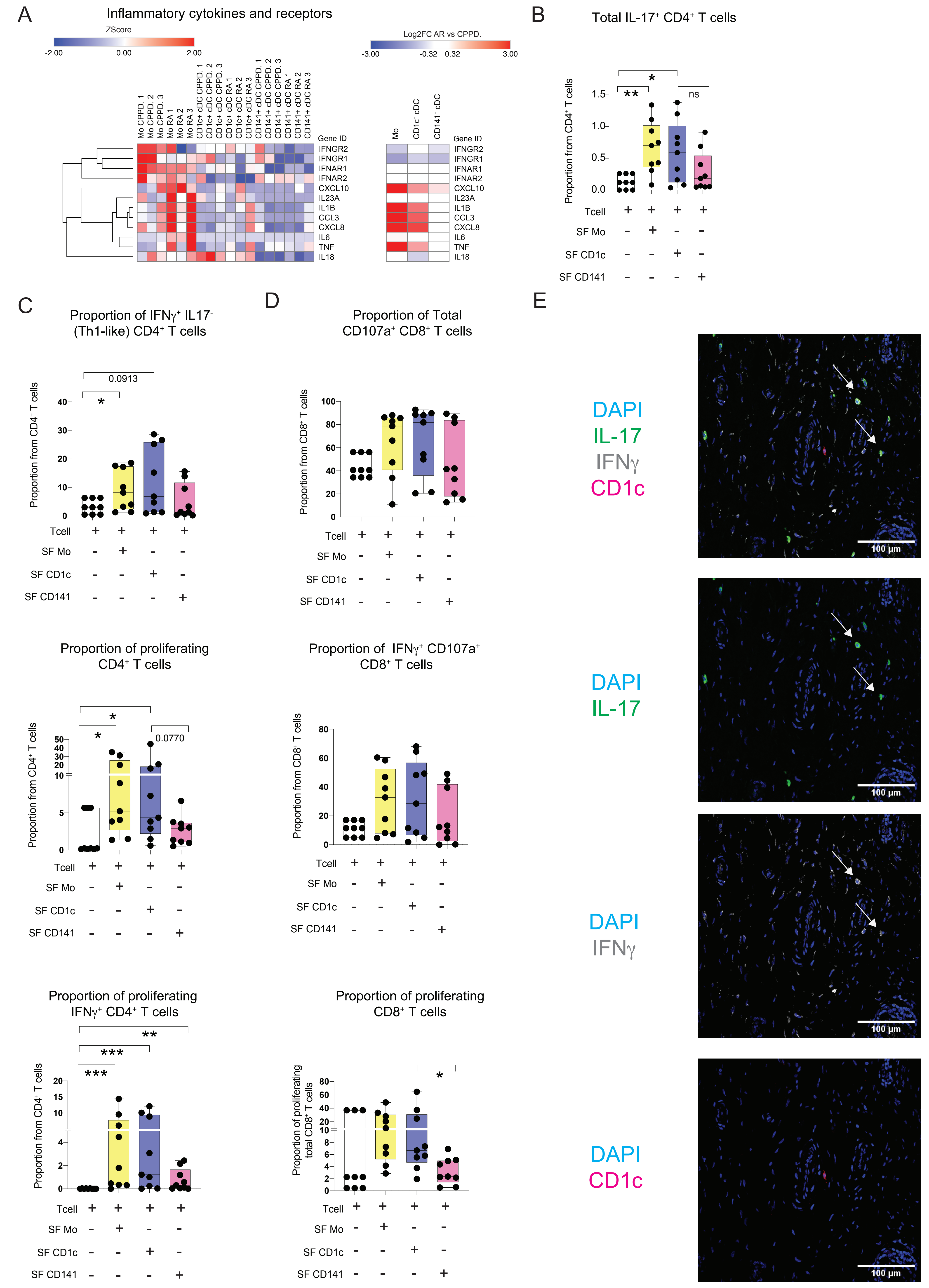

Supplemental Figure 7

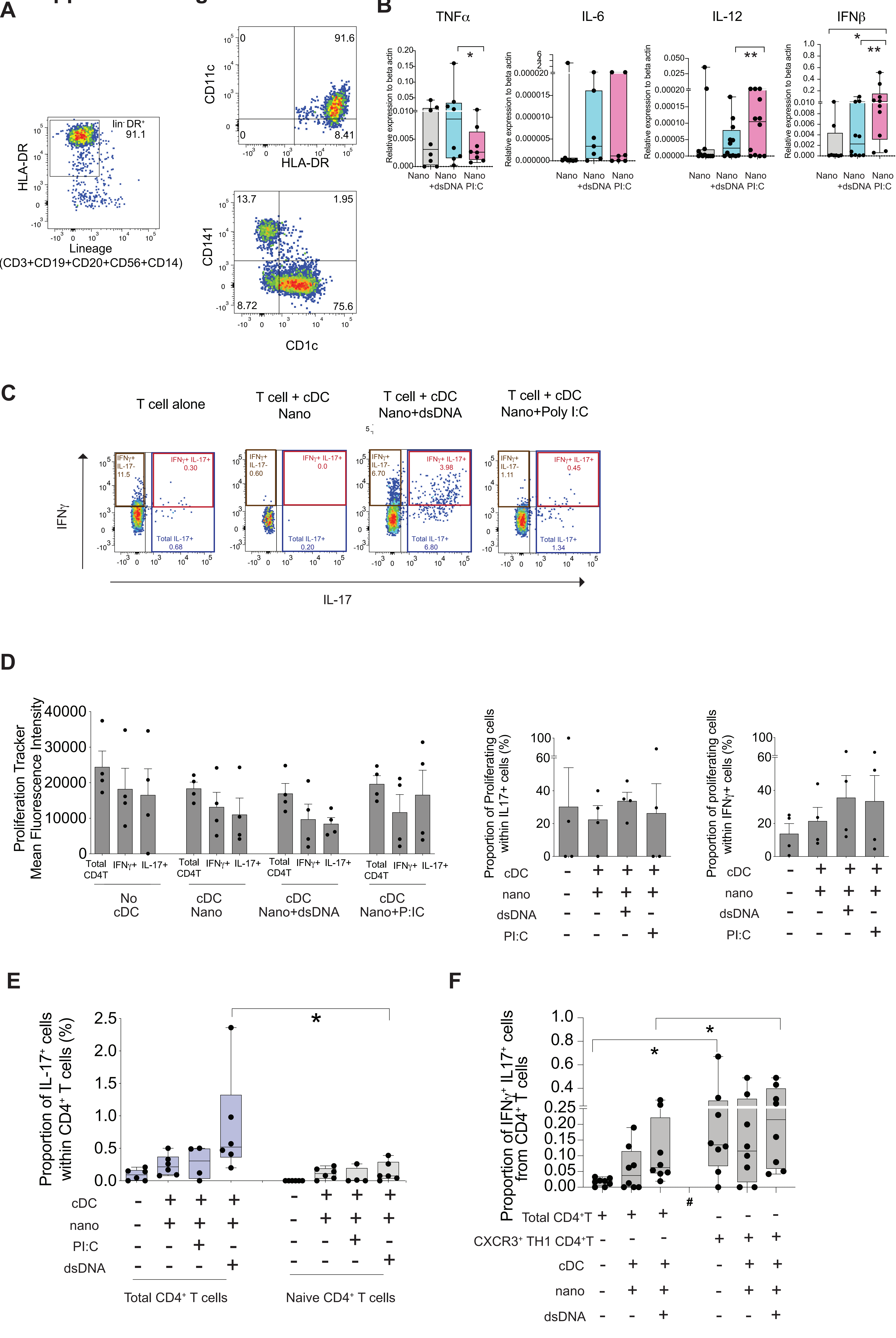

Supplemental Figure 8

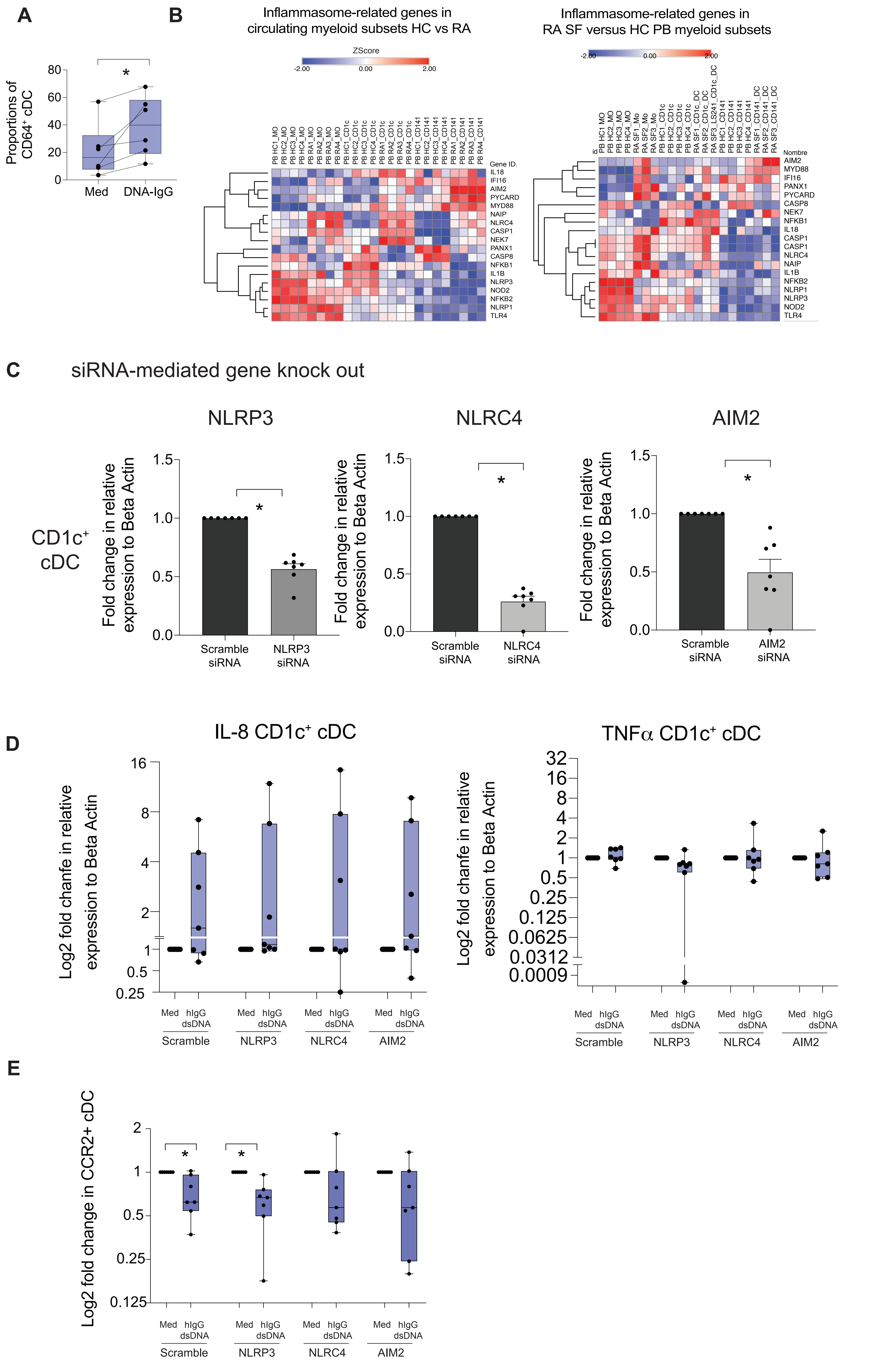

Supplemental Table 1. Clinical characteristics of untreated early arthritis patients and matched healthy controls recruited for PBMC collection in the study.

	RA patients	Sex and Age Matched Healthy controls
	(n =25)	(n =13)
Female; n (%)	23 (92)	12 (92.31)
Age; p50 [p25-p75]	44.38 [35.23 – 52.28]	46 [43 – 48]
RA / UA; n (%)	15 (60) / 10 (40)	
Disease duration (months); p50 [p25-p75]	4.96 [3 – 7.66]	
RF positive; n (%)	12 (48)	
ACPA positive; n (%)	12 (48)	
DAS28-ESR; p50 [p25-p75]	5.78 [2.70 – 6.11]	
HAQ; p50 [p25-p75]	0.87 [0.50 – 2]	

Abbreviations: n: number; p50: median or percentile 50; p25-p75: range between percentiles 25 and 75 or interquartile range; RA: rheumatoid arthritis; UA: undifferentiated arthritis; RF: rheumatoid factor; ACPA: anti-citrullinated protein antibodies; DAS28-ESR: baseline disease activity score estimated with the 28 joint count; HAQ: baseline health assessment questionnaire

Supplemental Table 2. Clinical characteristics of patients providing synovial fluid samples used for flow cytometry and RNA-seq studies.

	**Population**
	(n =16)
Female; n (%)	11 (68.75)
Age; p50 [p25-p75]	72 [56 – 81]
Diagnosis; n (% of patients)	
RA	7 (43.75)
Non RA	9 (56.25)
CPPD	4 (25.00)
SpA	2 (12.50)
Other arthritis	3 (18.75)
Treatment; n (%)	
Abatacept/Prednisone	1 (6.25)
Glucocorticoids	1 (6.25)
Leflunomide/Prednisone	1 (6.25)
MTX/Leflunomide	1 (6.25)
MTX/Prednisone	1 (6.25)
MTX/Rituximab	1 (6.25)
MTX/Secukinumab	1 (6.25)
NSAID	6 (37.5)
NSAID/colchicine	1 (6.25)
Prednisone	1 (6.25)
n.s.	1 (6.25)

Abbreviations: p50: median; p25-p75: interquartile range; RA: Rheumatoid Arthritis; SpA: Spondyloarthritis; MTX: methotrexate; NSAID: non steroid anti-inflammatory drugs; CPPD: Calcium Pyrophosphate Deposition Crystals-associated arthropathy; not specified: n.s

Supplemental Table 3. Clinical and treatment information of RA Patient samples used for longitudinal analysis.

	**Untreated RA patients Baseline**	**Treated RA patients 1-2 years**
	(n =14)	(n =14)
Female; n (%)	10 (71.43)	
Age; p50 [p25-p75]	54.99 [43.59-59.42]	
RA / UA; n (%)	14 (100) / 0 (0)	
RF positive; n (%)	7 (50)	
ACPA positive; n (%)	7 (50)	
Methotrexate; n (%)		11 (78.57)
Days with Methotrexate; p50 [p25-p75]		474 [377-725]
Dose Methotrexate (mg) ; p50 [p25-p75]		15 [10 -20]
Leflunomide; n (%)		3 (21.43)
Days with Leflunomide ; p50 [p25-p75]		363 [261-609]
Dose Leflunomide ; p50 [p25-p75]		20 [20-20]
Gold Salts; n (%)		1 (7.14)
Days with Gold salts ; p50 [p25-p75]		57 [57-57]
Dose Gold salts ; p50 [p25-p75]		200 [200-200]

Abbreviations: n: number; p50: median or percentile 50; p25-p75: range between percentiles 25 and 75 or interquartile range; RA: rheumatoid arthritis; UA: undifferentiated arthritis; RF: rheumatoid factor; ACPA: anti-citrullinated protein antibodies

Supplemental Table 4. Differentially expressed genes detected by RNA-seq in CD1c+ cDC from peripheral blood of n=4 rheumatoid arthritis (RA) *versus* n=4 healthy control (HC) individuals considering Log2FC >1.5 and <-1.5.

Transcipt ID	Gene name	Log2(FC) RA vs HC	pValue	FDR < 0.05
ENSG00000168329	CX3CR1	4,48061141	0,00037929	0,00790871
ENSG00000121807	CCR2	3,82961571	0,00220939	0,02137981
ENSG00000182578	CSF1R	3,76024591	0,00248329	0,02309163
ENSG00000111913	RIPOR2	3,53388674	1,5243E-06	0,00072361
ENSG00000101347	SAMHD1	3,50604418	0,00019459	0,00570753
ENSG00000140749	IGSF6	3,46377959	0,00057959	0,00984225
ENSG00000163683	SMIM14	3,44329664	2,5272E-05	0,00235489
ENSG00000110876	SELPLG	3,28994526	0,00446267	0,03329149
ENSG00000249437	NAIP	3,19506985	5,8815E-07	0,00049731
ENSG00000088986	DYNLL1	3,18041769	8,594E-05	0,00396731
ENSG00000139970	RTN1	3,15531368	0,00276867	0,02466053
ENSG00000124491	F13A1	3,11044269	0,00010576	0,0042443
ENSG00000133706	LARS	3,08687139	6,6015E-08	0,00014716
ENSG00000092964	DPYSL2	3,08658207	0,00134392	0,01593916
ENSG00000005020	SKAP2	3,07207885	1,0594E-06	0,00063084
ENSG00000179639	FCER1A	2,99303118	0,00200223	0,02017119
ENSG00000110077	MS4A6A	2,93279553	0,00015721	0,0051451
ENSG00000178927	C17orf62	2,90935819	7,361E-05	0,00370637
ENSG00000174123	TLR10	2,9089105	0,00043091	0,00844623
ENSG00000167220	HDHD2	2,86599238	1,5243E-07	0,00020766
ENSG00000133835	HSD17B4	2,8590816	0,00025039	0,00637563
ENSG00000148110	MFSD14B	2,84809917	2,9101E-05	0,00247091
ENSG00000177409	SAMD9L	2,84522583	4,4744E-06	0,00113545
ENSG00000163606	CD200R1	2,80754282	0,00060485	0,01007568
ENSG00000158517	NCF1	2,8049932	0,0056032	0,03848631
ENSG00000006715	VPS41	2,78696744	1,623E-05	0,00195084
ENSG00000175567	UCP2	2,74367436	0,00037172	0,00780397
ENSG00000161929	SCIMP	2,71741097	0,0036046	0,02910899
ENSG00000117676	RPS6KA1	2,67157659	0,00130031	0,01569137
ENSG00000197142	ACSL5	2,67137821	1,2461E-06	0,00067441
ENSG00000169413	RNASE6	2,63382722	0,00114346	0,01456565
ENSG00000170915	PAQR8	2,61926114	0,0051946	0,0367398
ENSG00000110031	LPXN	2,61258744	0,00039481	0,00807433
ENSG00000089127	OAS1	2,60907942	0,00177477	0,01877161
ENSG00000105483	CARD8	2,60439997	0,00026609	0,00657914
ENSG00000197043	ANXA6	2,59130743	0,00030611	0,0071693
ENSG00000175857	GAPT	2,58152619	0,00072136	0,01123558
ENSG00000127951	FGL2	2,52168178	0,00077143	0,01157559

ENSG00000135218	CD36	2,49600451	0,00332172	0,02758279
ENSG00000105281	SLC1A5	2,48772452	0,00506137	0,03611864
ENSG00000082074	FYB1	2,48238427	0,00201764	0,02024329
ENSG00000154930	ACSS1	2,47935522	0,00064796	0,01056432
ENSG00000093072	ADA2	2,47730298	0,0014379	0,01664721
ENSG00000125703	ATG4C	2,46599497	3,6471E-08	9,9367E-05
ENSG00000165168	CYBB	2,45698664	2,5284E-05	0,00235489
ENSG00000117054	ACADM	2,45339911	0,00052518	0,00933176
ENSG00000155097	ATP6V1C1	2,43300842	2,2249E-05	0,00223591
ENSG00000133943	DGLUCY	2,42967609	1,7473E-05	0,0019819
ENSG00000122986	HVCN1	2,42653105	0,00520796	0,03678399
ENSG00000145781	COMMD10	2,41876818	2,1757E-05	0,00220458
ENSG00000196975	ANXA4	2,4143072	0,00016641	0,00527776
ENSG00000164125	FAM198B	2,40708778	0,00077917	0,01164368
ENSG00000111335	OAS2	2,40350988	0,0003081	0,00719047
ENSG00000139641	ESYT1	2,3946385	0,00027209	0,00664527
ENSG00000170581	STAT2	2,38560484	0,00043299	0,00846677
ENSG00000164062	APEH	2,35970332	0,00063912	0,01046185
ENSG00000240065	PSMB9	2,35653428	0,00062402	0,01027881
ENSG00000165672	PRDX3	2,35012712	2,102E-06	0,00079299
ENSG00000113845	TIMMDC1	2,33704748	5,2769E-08	0,0001294
ENSG00000102893	PHKB	2,33288177	0,00022773	0,00618155
ENSG00000186088	GSAP	2,32970547	0,00039995	0,00815225
ENSG00000136485	DCAF7	2,31911671	0,00100774	0,01352533
ENSG00000197713	RPE	2,31791674	0,00010468	0,00422125
ENSG00000132514	CLEC10A	2,30730983	0,00055124	0,00954101
ENSG00000168310	IRF2	2,30304339	9,3187E-06	0,00157323
ENSG00000073849	ST6GAL1	2,30048546	0,00366277	0,02938002
ENSG00000104972	LILRB1	2,28750334	0,00160309	0,01767505
ENSG00000198771	RCSD1	2,27632314	1,087E-05	0,00167542
ENSG00000188554	NBR1	2,27288862	9,6062E-05	0,0040683
ENSG00000107551	RASSF4	2,27141897	0,00255776	0,02350784
ENSG00000136631	VPS45	2,26964348	3,5717E-05	0,00258691
ENSG00000145416	MARCH1	2,2678262	7,018E-05	0,00362046
ENSG00000082996	RNF13	2,25866168	9,4402E-05	0,00406059
ENSG00000084733	RAB10	2,25708789	0,00028046	0,00675657
ENSG00000093144	ECHDC1	2,25700032	0,00050395	0,00910856
ENSG00000116990	MYCL	2,25587312	0,00203949	0,02039571
ENSG00000065413	ANKRD44	2,24912525	2,5433E-06	0,0008909
ENSG00000103051	COG4	2,23980254	2,7994E-05	0,00244281
ENSG00000184432	COPB2	2,23644128	0,00114942	0,0145809
ENSG00000035403	VCL	2,23165574	9,5618E-06	0,00157323
ENSG00000138119	MYOF	2,22086902	0,00049806	0,00907344

ENSG00000132182	NUP210	2,21717303	2,7083E-05	0,00242375
ENSG00000135549	PKIB	2,21280839	0,00093318	0,01293522
ENSG00000112118	MCM3	2,20587762	3,1291E-05	0,00247091
ENSG00000164414	SLC35A1	2,20257592	0,00040265	0,00816665
ENSG00000196209	SIRPB2	2,19971041	0,00118233	0,01480687
ENSG00000090861	AARS	2,1976883	6,3164E-06	0,00130246
ENSG00000151702	FLI1	2,19655911	9,8468E-05	0,0041274
ENSG00000150681	RGS18	2,19573473	0,00050414	0,00910856
ENSG00000214114	MYCBP	2,18050468	6,5063E-05	0,00350639
ENSG00000107929	LARP4B	2,18016296	0,00012087	0,00451687
ENSG00000174944	P2RY14	2,17849686	0,00429313	0,03251137
ENSG00000059377	TBXAS1	2,17545683	0,00217222	0,02116209
ENSG00000116455	WDR77	2,17357757	0,00040834	0,00827509
ENSG00000143624	INTS3	2,17283674	0,00019661	0,00571762
ENSG00000160593	JAML	2,16917921	0,00022495	0,00614493
ENSG00000184992	BRI3BP	2,16506409	4,8554E-05	0,00304437
ENSG00000163154	TNFAIP8L2	2,16050941	0,00196356	0,01997036
ENSG00000138459	SLC35A5	2,16045603	0,00432107	0,03263973
ENSG00000100364	KIAA0930	2,1588752	0,00170495	0,01830649
ENSG00000134986	NREP	2,15294624	5,2178E-05	0,00318478
ENSG00000103381	CPPED1	2,1428926	0,00601614	0,04029549
ENSG00000074706	IPCEF1	2,12515763	0,00102215	0,0136367
ENSG00000136279	DBNL	2,12475187	6,406E-05	0,00347939
ENSG00000091106	NLRC4	2,12153991	0,00422623	0,03221366
ENSG00000243749	TMEM35B	2,12081502	1,2145E-05	0,00174153
ENSG00000145348	TBCK	2,11983735	0,00054632	0,00951443
ENSG00000075303	SLC25A40	2,11438947	0,00052717	0,00933176
ENSG00000134851	TMEM165	2,1112914	0,00053025	0,00934747
ENSG00000188641	DPYD	2,10810019	7,1888E-05	0,00366613
ENSG00000166326	TRIM44	2,09588341	1,3385E-05	0,00177415
ENSG00000136518	ACTL6A	2,09472527	0,00051119	0,0091718
ENSG00000148634	HERC4	2,09144461	0,00634886	0,04184958
ENSG00000132530	XAF1	2,08556349	0,00042117	0,00840325
ENSG00000104974	LILRA1	2,07555488	0,00331208	0,02754529
ENSG00000144218	AFF3	2,0746972	0,00339371	0,02798159
ENSG00000204261	PSMB8-AS1	2,07449864	0,00033371	0,00750029
ENSG00000138413	IDH1	2,07270266	0,00017122	0,00534257
ENSG00000258659	TRIM34	2,07242747	0,00022782	0,00618155
ENSG00000117632	STMN1	2,07063214	8,1391E-07	0,00058408
ENSG00000112367	FIG4	2,06010715	0,0010552	0,0138991
ENSG00000181381	DDX60L	2,05825221	0,00321941	0,02698914
ENSG00000099810	MTAP	2,04310849	0,00041693	0,00834397
ENSG00000134910	STT3A	2,03550427	0,00297555	0,02573666

ENSG00000141510	TP53	2,03263683	0,00027892	0,00673159
ENSG00000133574	GIMAP4	2,02455475	0,00165405	0,0179623
ENSG00000081087	OSTM1	2,01699248	0,00038293	0,00795755
ENSG00000166888	STAT6	2,01589534	0,00014176	0,00483474
ENSG00000072501	SMC1A	2,00810478	0,00233103	0,0222236
ENSG00000183486	MX2	2,00691253	2,7828E-05	0,00243708
ENSG00000050344	NFE2L3	2,00427228	0,00311514	0,02640052
ENSG00000090863	GLG1	2,00228223	2,1579E-05	0,00219563
ENSG00000180353	HCLS1	2,00117507	0,00032567	0,00741474
ENSG00000131844	MCCC2	1,99873949	4,7713E-05	0,00303099
ENSG00000197798	FAM118B	1,98193844	0,00452146	0,0335553
ENSG00000144468	RHBDD1	1,9790882	3,445E-05	0,00258668
ENSG00000155957	TMBIM4	1,97882377	1,4529E-07	0,00020766
ENSG00000204131	NHSL2	1,96937991	0,00262472	0,02390812
ENSG00000014919	COX15	1,96845915	5,5703E-05	0,00328338
ENSG00000132646	PCNA	1,96010266	9,2288E-05	0,00404409
ENSG00000163563	MNDA	1,9575603	0,00017169	0,00534257
ENSG00000146192	FGD2	1,95571479	0,00359128	0,02908251
ENSG00000137628	DDX60	1,95489513	0,00022884	0,00618155
ENSG00000121210	TMEM131L	1,95201873	0,00023246	0,00619904
ENSG00000079950	STX7	1,9492398	0,00068282	0,01090059
ENSG00000026751	SLAMF7	1,94753635	0,00054928	0,00953893
ENSG00000130021	PUDP	1,94733736	0,00662426	0,04310783
ENSG00000160310	PRMT2	1,94681729	0,00168156	0,01816459
ENSG00000187688	TRPV2	1,9424128	0,00360365	0,02910899
ENSG00000166002	SMCO4	1,9401174	0,00020978	0,00591255
ENSG00000166797	FAM96A	1,93885909	2,1485E-05	0,00219518
ENSG00000134256	CD101	1,938281	0,00665078	0,04321243
ENSG00000040933	INPP4A	1,93825604	1,7308E-05	0,0019819
ENSG00000166801	FAM111A	1,93694383	0,00198427	0,02005975
ENSG00000004455	AK2	1,92554196	0,00011655	0,00445151
ENSG00000124357	NAGK	1,92531085	0,00114593	0,01457675
ENSG00000101336	HCK	1,9207607	0,00142293	0,01652489
ENSG00000159228	CBR1	1,91788964	7,5042E-05	0,00373753
ENSG00000125863	MKKS	1,91758787	0,00201031	0,02019972
ENSG00000130309	COLGALT1	1,91512685	0,00073415	0,01131434
ENSG00000064115	TM7SF3	1,91202276	0,00011004	0,00429678
ENSG00000143799	PARP1	1,90822374	1,5479E-05	0,00191997
ENSG00000280153	AC133065.6	1,90788151	1,4511E-06	0,00071859
ENSG00000103479	RBL2	1,9056599	7,6788E-06	0,00141771
ENSG00000111481	COPZ1	1,90112607	0,00023186	0,00619904
ENSG00000274536	AL034397.3	1,90103507	0,0002185	0,00605543
ENSG00000048052	HDAC9	1,90086053	0,00293813	0,02554823

ENSG00000187554	TLR5	1,89883198	1,7626E-05	0,00198257
ENSG00000149311	ATM	1,89458291	0,00032215	0,00737585
ENSG00000169220	RGS14	1,89323786	0,00021884	0,00605543
ENSG00000124356	STAMBP	1,88960865	6,3999E-06	0,00130777
ENSG00000121281	ADCY7	1,88955827	0,00475444	0,03473888
ENSG00000138078	PREPL	1,884667	0,00067772	0,0108546
ENSG00000125124	BBS2	1,87972798	0,00123045	0,01509351
ENSG00000123106	CCDC91	1,87738933	0,00064878	0,01056603
ENSG00000103423	DNAJA3	1,87709108	8,0711E-05	0,00388825
ENSG00000157637	SLC38A10	1,87635036	0,00182582	0,01910843
ENSG00000153071	DAB2	1,87389799	0,00206746	0,02056638
ENSG00000136040	PLXNC1	1,87260536	0,0037511	0,0298639
ENSG00000165178	NCF1C	1,87195637	0,00059169	0,00992395
ENSG00000213445	SIPA1	1,86661479	1,7269E-05	0,0019819
ENSG00000134318	ROCK2	1,86171155	0,00016368	0,00524714
ENSG00000109911	ELP4	1,86062956	0,00022455	0,00614493
ENSG00000135317	SNX14	1,85797198	8,9103E-09	5,4622E-05
ENSG00000162736	NCSTN	1,85502857	0,00022669	0,00618155
ENSG00000133313	CNDP2	1,85377486	0,00461658	0,03403916
ENSG00000143252	SDHC	1,85172045	0,00010663	0,00425732
ENSG00000100030	MAPK1	1,84978086	1,7539E-05	0,0019819
ENSG00000112079	STK38	1,84798966	0,00180769	0,01895907
ENSG00000134014	ELP3	1,84755915	5,9244E-05	0,00338854
ENSG00000170006	TMEM154	1,84121516	0,00016724	0,00527776
ENSG00000168538	TRAPPC11	1,83298923	4,8259E-05	0,0030432
ENSG00000171766	GATM	1,83086753	0,00658812	0,04294187
ENSG00000134255	CEPT1	1,82860878	0,00013528	0,00477982
ENSG00000102699	PARP4	1,82823123	0,00036217	0,00772242
ENSG00000159131	GART	1,82773346	0,00141094	0,0164282
ENSG00000160712	IL6R	1,82646145	0,00198462	0,02005975
ENSG00000132383	RPA1	1,82456052	0,00187832	0,01936854
ENSG00000115234	SNX17	1,82349364	0,00012503	0,00460347
ENSG00000107201	DDX58	1,82223905	8,3749E-06	0,00149899
ENSG00000106785	TRIM14	1,82209559	0,00024494	0,00630241
ENSG00000010671	BTK	1,82005531	9,6383E-06	0,00157323
ENSG00000139508	SLC46A3	1,81990555	0,00107366	0,01403634
ENSG00000150867	PIP4K2A	1,81392909	0,00017616	0,0054335
ENSG00000134452	FBXO18	1,81303052	0,00012753	0,00467205
ENSG00000103544	C16orf62	1,80980864	0,00044119	0,00851842
ENSG00000107099	DOCK8	1,80821302	0,00156579	0,01745211
ENSG00000008869	HEATR5B	1,80620624	0,00114032	0,01454071
ENSG00000197943	PLCG2	1,8049588	0,00133262	0,01588805
ENSG00000014216	CAPN1	1,80223522	0,00017916	0,00547016

ENSG00000111667	USP5	1,80219568	8,7783E-05	0,00399188
ENSG00000140395	WDR61	1,80087709	2,1332E-07	0,00024909
ENSG00000256043	CTSO	1,79973505	3,6587E-05	0,00258691
ENSG00000138246	DNAJC13	1,79880305	0,00046729	0,00879473
ENSG00000070785	EIF2B3	1,78811279	9,0415E-05	0,00402538
ENSG00000162704	ARPC5	1,78537794	0,00023852	0,00624858
ENSG00000181192	DHTKD1	1,78454455	3,879E-05	0,00266433
ENSG00000096968	JAK2	1,78368132	0,00077183	0,01157559
ENSG00000243927	MRPS6	1,78329767	0,00037785	0,00788531
ENSG00000172269	DPAGT1	1,7788743	3,6717E-05	0,00258691
ENSG00000092531	SNAP23	1,77861236	0,00017503	0,00541218
ENSG00000007923	DNAJC11	1,7769984	0,00071439	0,01120142
ENSG00000110917	MLEC	1,77564297	0,00033974	0,00753305
ENSG00000155926	SLA	1,77445735	0,00030888	0,00719293
ENSG00000164494	PDSS2	1,772626	9,0514E-07	0,00058408
ENSG00000130150	MOSPD2	1,77230019	0,00171183	0,0183567
ENSG00000127946	HIP1	1,76967345	0,00294765	0,02560375
ENSG00000145246	ATP10D	1,76898507	6,0141E-05	0,00340579
ENSG00000118596	SLC16A7	1,76754091	0,00153256	0,01725431
ENSG00000033050	ABCF2	1,76635713	8,0997E-06	0,00148218
ENSG00000113522	RAD50	1,76599828	0,00017232	0,00535533
ENSG00000128915	ICE2	1,76558885	0,00074477	0,01140723
ENSG00000131828	PDHA1	1,76388484	0,0004139	0,0083328
ENSG00000156110	ADK	1,75376453	0,00059746	0,00998656
ENSG00000058668	ATP2B4	1,75365865	0,00112853	0,01445049
ENSG00000165071	TMEM71	1,74845268	0,0005813	0,00986448
ENSG00000198951	NAGA	1,7482429	0,00245709	0,02292632
ENSG00000167797	CDK2AP2	1,74657394	0,00069689	0,01103198
ENSG00000196510	ANAPC7	1,74350096	0,00086873	0,01231659
ENSG00000110079	MS4A4A	1,74015601	5,9103E-05	0,00338854
ENSG00000106049	HIBADH	1,73647252	0,00010394	0,00421955
ENSG00000129675	ARHGEF6	1,73332951	0,000278	0,00672283
ENSG00000104325	DECR1	1,73243016	0,000493	0,00902828
ENSG00000165476	REEP3	1,72862276	0,00030022	0,00711948
ENSG00000085224	ATRX	1,72685376	9,3561E-05	0,00404623
ENSG00000010292	NCAPD2	1,72683475	8,4432E-07	0,00058408
ENSG00000102189	EEA1	1,72613896	3,7848E-06	0,00109843
ENSG00000100442	FKBP3	1,72590534	4,6332E-05	0,0029741
ENSG00000118961	LDAH	1,72553287	0,00096201	0,0131815
ENSG00000143110	C1orf162	1,72259036	0,00626623	0,04142966
ENSG00000280071	FP565260.6	1,71311392	0,00021978	0,00606891
ENSG00000134955	SLC37A2	1,71209736	9,0029E-05	0,00402422
ENSG00000111540	RAB5B	1,71147472	3,3654E-05	0,00257887

ENSG00000149573	MPZL2	1,70877446	0,0003976	0,00811784
ENSG00000188352	FOCAD	1,7027923	1,783E-06	0,00072361
ENSG00000137752	CASP1	1,70148715	4,3937E-06	0,00113545
ENSG00000136169	SETDB2	1,69379799	0,00016233	0,00522738
ENSG00000134996	OSTF1	1,69345603	0,0002535	0,00640971
ENSG00000120860	WASHC3	1,69330411	1,6403E-05	0,00196208
ENSG00000088888	MAVS	1,69116304	0,00023213	0,00619904
ENSG00000170632	ARMC10	1,68966314	0,00073702	0,01133096
ENSG00000186470	BTN3A2	1,68720215	0,00042906	0,00843962
ENSG00000075239	ACAT1	1,68482328	0,00025355	0,00640971
ENSG00000178537	SLC25A20	1,68408896	0,00188556	0,01942682
ENSG00000185722	ANKFY1	1,67702254	0,00017587	0,00543132
ENSG00000101096	NFATC2	1,66964172	0,00010702	0,00425732
ENSG00000104998	IL27RA	1,66864811	0,00541958	0,03769313
ENSG00000102710	SUPT20H	1,66707065	0,00039008	0,00804467
ENSG00000132256	TRIM5	1,6668103	4,2975E-05	0,00284526
ENSG00000170854	RIOX2	1,66589789	0,00022939	0,00618155
ENSG00000143106	PSMA5	1,6596845	0,00019816	0,00573684
ENSG00000183597	TANGO2	1,65951463	0,00021451	0,00600181
ENSG00000133103	COG6	1,65795453	0,00085227	0,01216754
ENSG00000087338	GMCL1	1,65556265	6,8351E-05	0,00360489
ENSG00000159063	ALG8	1,6548367	0,00559617	0,03845953
ENSG00000119471	HSDL2	1,65369752	0,00431639	0,03263715
ENSG00000151665	PIGF	1,65119022	8,2969E-05	0,00391999
ENSG00000155229	MMS19	1,64938365	0,00010455	0,00422125
ENSG00000163565	IFI16	1,64583944	0,00110117	0,01422749
ENSG00000183978	COA3	1,64218892	6,646E-05	0,00355049
ENSG00000157483	MYO1E	1,64094009	0,00579626	0,03934944
ENSG00000109436	TBC1D9	1,63906624	4,485E-05	0,00291714
ENSG00000143390	RFX5	1,63616126	0,00018465	0,00554421
ENSG00000142687	KIAA0319L	1,63465456	0,00084454	0,01210519
ENSG00000176783	RUFY1	1,63259568	0,0004159	0,00834397
ENSG00000185432	METTL7A	1,63086959	6,3544E-05	0,00347031
ENSG00000081189	MEF2C	1,63075264	6,8781E-05	0,00360489
ENSG00000188321	ZNF559	1,63009183	0,00120256	0,01492471
ENSG00000104518	GSDMD	1,62758197	0,00030767	0,00719047
ENSG00000178685	PARP10	1,62568346	0,00018499	0,00554421
ENSG00000250687	AC146944.2	1,62211084	0,00041211	0,00830353
ENSG00000158467	AHCYL2	1,6220047	0,00032675	0,00741876
ENSG00000009790	TRAF3IP3	1,6209906	0,00039389	0,00807433
ENSG00000155850	SLC26A2	1,61861679	0,00046141	0,00874028
ENSG00000149196	HIKESHI	1,61843099	1,9633E-06	0,00075221
ENSG00000197157	SND1	1,61796547	0,00046841	0,00879473

ENSG00000132405	TBC1D14	1,61793217	2,5564E-05	0,00235489
ENSG00000124532	MRS2	1,61642814	5,9991E-05	0,0034052
ENSG00000130985	UBA1	1,61608016	0,00338801	0,02795335
ENSG00000118855	MFSD1	1,60912431	0,00014683	0,00494973
ENSG00000196511	TPK1	1,60908018	0,00276372	0,02466022
ENSG00000151116	UEVLD	1,60878312	0,0005791	0,00984225
ENSG00000254505	CHMP4A	1,60519077	0,00073797	0,01133105
ENSG00000068079	IFI35	1,60370017	0,00019552	0,00570755
ENSG00000139278	GLIPR1	1,6002492	0,00058493	0,00987721
ENSG00000088682	COQ9	1,60021544	0,00032123	0,00736161
ENSG00000030066	NUP160	1,5970384	0,00105587	0,0138991
ENSG00000116406	EDEM3	1,59653967	0,00590848	0,03983553
ENSG00000168710	AHCYL1	1,59560036	0,00041977	0,00838212
ENSG00000196730	DAPK1	1,59552344	0,0006625	0,0107158
ENSG00000040341	STAU2	1,59299663	0,00011326	0,00435519
ENSG00000185305	ARL15	1,58897636	0,0015847	0,0175574
ENSG00000136100	VPS36	1,5871675	0,00022423	0,00614493
ENSG00000198814	GK	1,58649058	0,000436	0,0084912
ENSG00000163933	RFT1	1,58636589	0,00024414	0,00629335
ENSG00000110665	C11orf21	1,582826	0,00153674	0,01727464
ENSG00000184178	SCFD2	1,58061375	0,00279391	0,02475948
ENSG00000078081	LAMP3	1,58037164	1,785E-06	0,00072361
ENSG00000198700	IPO9	1,57820554	0,00142503	0,01653719
ENSG00000102316	MAGED2	1,57762468	0,0002993	0,00711168
ENSG00000155846	PPARGC1B	1,57018146	0,00189369	0,01950242
ENSG00000159200	RCAN1	1,5685297	0,00626578	0,04142966
ENSG00000163932	PRKCD	1,566364	0,00240727	0,02264239
ENSG00000104299	INTS9	1,56476881	4,9216E-05	0,00304896
ENSG00000115204	MPV17	1,56212816	0,0002115	0,00594075
ENSG00000128340	RAC2	1,56068114	0,0006815	0,01090059
ENSG00000075292	ZNF638	1,56059318	0,00022799	0,00618155
ENSG00000166479	TMX3	1,55957644	0,00225671	0,02172622
ENSG00000111653	ING4	1,55857715	2,4958E-05	0,0023538
ENSG00000137509	PRCP	1,55794289	0,00755677	0,04703037
ENSG00000100304	TTLL12	1,55626777	0,00079881	0,01176428
ENSG00000104365	IKBKB	1,55620465	0,00014605	0,00493969
ENSG00000108622	ICAM2	1,55449847	0,00350252	0,02866665
ENSG00000172086	KRCC1	1,55408178	0,00197364	0,02002285
ENSG00000121486	TRMT1L	1,55272096	6,347E-05	0,00347031
ENSG00000138660	AP1AR	1,54636611	0,00034697	0,00755632
ENSG00000128928	IVD	1,54602401	3,7887E-05	0,00260964
ENSG00000178695	KCTD12	1,5457285	0,00677466	0,04372765
ENSG00000189091	SF3B3	1,5446092	3,6572E-05	0,00258691

ENSG00000198876	DCAF12	1,54444489	0,00071513	0,01120142
ENSG00000099194	SCD	1,54415832	0,00062647	0,01029685
ENSG00000196305	IARS	1,53948705	0,00078136	0,01164368
ENSG00000243943	ZNF512	1,53922632	6,8792E-05	0,00360489
ENSG00000148218	ALAD	1,53801383	0,00441851	0,03306262
ENSG00000112685	EXOC2	1,53703903	0,00058462	0,00987721
ENSG00000139291	TMEM19	1,53469373	0,00035706	0,00766855
ENSG00000155660	PDIA4	1,53424555	0,00154319	0,01729986
ENSG00000074201	CLNS1A	1,53399002	0,00031894	0,00732956
ENSG00000105639	JAK3	1,53396236	0,00065778	0,01066754
ENSG00000104812	GYS1	1,53281396	0,00444366	0,03321802
ENSG00000119912	IDE	1,53194049	0,00271768	0,02444613
ENSG00000008130	NADK	1,53125751	0,00023416	0,00621422
ENSG00000128731	HERC2	1,52713891	1,6122E-05	0,00195084
ENSG00000204713	TRIM27	1,52629045	0,00133829	0,01592242
ENSG00000219545	UMAD1	1,52621874	0,00017813	0,00546683
ENSG00000140575	IQGAP1	1,52480599	0,00214008	0,02098685
ENSG00000221817	PPP3CB-AS1	1,52298093	0,00018578	0,00554421
ENSG00000136824	SMC2	1,52223807	0,00402461	0,03123025
ENSG00000139163	ETNK1	1,52207688	0,00078825	0,0116915
ENSG00000106034	CPED1	1,51917466	0,00165901	0,01798439
ENSG00000214078	CPNE1	1,5174491	0,00249304	0,02313844
ENSG00000247556	OIP5-AS1	1,51735596	0,00270808	0,02440763
ENSG00000028277	POU2F2	1,51689626	5,8105E-05	0,00336037
ENSG00000214013	GANC	1,51512885	1,1944E-05	0,00172682
ENSG00000120008	WDR11	1,51453886	0,00060752	0,01011337
ENSG00000137478	FCHSD2	1,51434924	0,00589139	0,03976406
ENSG00000018699	TTC27	1,51402038	3,0772E-05	0,00247091
ENSG00000123689	G0S2	-7,3437378	0,00359531	0,02909593
ENSG00000090104	RGS1	-4,9226152	0,00284958	0,02508171
ENSG00000113448	PDE4D	-4,6161911	0,00238124	0,02250114
ENSG00000112715	VEGFA	-4,3980717	0,00798186	0,04873585
ENSG00000095794	CREM	-4,2324773	0,00234142	0,02225351
ENSG00000131669	NINJ1	-4,2256981	8,4938E-05	0,00395962
ENSG00000088826	SMOX	-4,1917929	0,0062834	0,04149614
ENSG00000161921	CXCL16	-4,0852069	0,00093127	0,01292339
ENSG00000120063	GNA13	-4,0117319	0,00341068	0,02808777
ENSG00000113070	HBEGF	-3,9180449	0,00165633	0,01796323
ENSG00000169155	ZBTB43	-3,9152501	1,703E-06	0,00072361
ENSG00000186594	MIR22HG	-3,9033207	0,00031213	0,00724792
ENSG00000166920	C15orf48	-3,8726255	0,00206516	0,02056638
ENSG00000118515	SGK1	-3,8284618	0,00015865	0,00516159
ENSG00000100644	HIF1A	-3,8142279	0,00020787	0,0058883

ENSG00000169508	GPR183	-3,8011148	3,3133E-06	0,00105137
ENSG00000168036	CTNNB1	-3,7994288	0,00075164	0,01143762
ENSG00000112149	CD83	-3,7781679	0,0068626	0,04408971
ENSG00000104312	RIPK2	-3,7714782	0,0002475	0,00635483
ENSG00000170525	PFKFB3	-3,6547474	0,00539139	0,0375788
ENSG00000123358	NR4A1	-3,6002194	4,8902E-06	0,00116551
ENSG00000008083	JARID2	-3,5873303	0,00210027	0,02074134
ENSG00000105835	NAMPT	-3,565579	0,0035389	0,0288393
ENSG00000153234	NR4A2	-3,5240212	0,00406834	0,03144339
ENSG00000155307	SAMSN1	-3,5110917	0,00090081	0,01263658
ENSG00000075426	FOSL2	-3,4696744	0,00269874	0,02438315
ENSG00000135604	STX11	-3,4604487	7,5449E-05	0,00373753
ENSG00000167996	FTH1	-3,4455424	0,00014065	0,0048288
ENSG00000162496	DHRS3	-3,4281745	0,00238119	0,02250114
ENSG00000217801	AL390719.1	-3,4129673	0,00765756	0,04740497
ENSG00000122644	ARL4A	-3,3881954	0,00051131	0,0091718
ENSG00000184205	TSPYL2	-3,3691133	0,00234818	0,02229371
ENSG00000160223	ICOSLG	-3,3006313	0,00048174	0,0089086
ENSG00000140743	CDR2	-3,2995217	0,00091354	0,01275674
ENSG00000163376	KBTBD8	-3,2941765	0,00049193	0,00901549
ENSG00000154640	BTG3	-3,2904506	1,1769E-05	0,00172682
ENSG00000145860	RNF145	-3,266945	3,4804E-07	0,00035913
ENSG00000143507	DUSP10	-3,2582835	0,00051351	0,00919784
ENSG00000011422	PLAUR	-3,2576446	0,00052867	0,00933176
ENSG00000231721	LINC-PINT	-3,2519897	0,00299457	0,02588294
ENSG00000162772	ATF3	-3,2383567	9,7321E-05	0,00410742
ENSG00000173166	RAPH1	-3,2330503	0,0037718	0,02998976
ENSG00000165030	NFIL3	-3,2231619	0,00031284	0,00725755
ENSG00000099985	OSM	-3,1985254	0,00035458	0,00764037
ENSG00000180530	NRIP1	-3,1974701	9,1247E-05	0,00402538
ENSG00000132906	CASP9	-3,1930446	0,00635242	0,0418618
ENSG00000166128	RAB8B	-3,1858229	0,00426519	0,03237977
ENSG00000277632	CCL3	-3,1722004	2,4131E-05	0,00232537
ENSG00000255073	ZFP91-CNTF	-3,1210466	9,7693E-05	0,00410899
ENSG00000185022	MAFF	-3,1036688	0,00152327	0,01718125
ENSG00000140564	FURIN	-3,0914186	0,00362575	0,02918809
ENSG00000169895	SYAP1	-3,0818613	0,00426163	0,03237283
ENSG00000057657	PRDM1	-3,066736	0,00273727	0,02454103
ENSG00000259884	AC025259.3	-3,0396069	6,6133E-06	0,00131841
ENSG00000272888	LINC01578	-3,0132913	0,00111079	0,01432376
ENSG00000059728	MXD1	-2,9742484	0,00046198	0,00874028
ENSG00000120705	ETF1	-2,96736	0,00061556	0,01019874
ENSG00000059804	SLC2A3	-2,9612668	0,00383134	0,03023761

ENSG00000221869	CEBPD	-2,9607654	9,5534E-06	0,00157323
ENSG00000162711	NLRP3	-2,9523592	0,0025683	0,02354519
ENSG00000106004	HOXA5	-2,9484577	0,00133377	0,01588805
ENSG00000277117	FP565260.3	-2,9438277	0,0010183	0,01361128
ENSG00000117036	ETV3	-2,9299673	0,0066802	0,04335762
ENSG00000174738	NR1D2	-2,9271361	5,1845E-06	0,00119932
ENSG00000173334	TRIB1	-2,9113062	0,0031129	0,02640052
ENSG00000087074	PPP1R15A	-2,8937247	0,00075601	0,01145649
ENSG00000161835	GRASP	-2,8887151	0,00135249	0,01601374
ENSG00000125538	IL1B	-2,8806117	0,00058656	0,00987721
ENSG00000141682	PMAIP1	-2,857255	9,4933E-05	0,00406059
ENSG00000134107	BHLHE40	-2,8520684	0,00042873	0,00843962
ENSG00000102760	RGCC	-2,849535	0,00025673	0,00644618
ENSG00000130340	SNX9	-2,8272045	0,00134383	0,01593916
ENSG00000120129	DUSP1	-2,8174266	0,00052671	0,00933176
ENSG00000271614	ATP2B1-AS1	-2,8128714	0,00015494	0,00510664
ENSG00000125657	TNFSF9	-2,8075585	3,1878E-05	0,00249272
ENSG00000197170	PSMD12	-2,7951382	1,276E-06	0,00067441
ENSG00000102908	NFAT5	-2,7828521	0,00091732	0,01280232
ENSG00000114784	EIF1B	-2,7799349	1,9323E-05	0,00209417
ENSG00000211445	GPX3	-2,7773061	0,00406162	0,03141797
ENSG00000121966	CXCR4	-2,763678	0,00036551	0,00772987
ENSG00000131408	NR1H2	-2,761791	0,00050954	0,00916688
ENSG00000124762	CDKN1A	-2,7611648	0,00013491	0,00477951
ENSG00000175040	CHST2	-2,7558327	0,00732683	0,0460788
ENSG00000100906	NFKBIA	-2,747788	7,5289E-06	0,00141771
ENSG00000115165	CYTIP	-2,7401929	0,00043019	0,00843962
ENSG00000011566	MAP4K3	-2,736966	0,0008043	0,01181026
ENSG00000118689	FOXO3	-2,7360317	0,00031782	0,00731077
ENSG00000177606	JUN	-2,7318881	0,00170161	0,01830053
ENSG00000015475	BID	-2,7233884	0,00479351	0,03494101
ENSG00000123091	RNF11	-2,7101978	9,5726E-05	0,0040683
ENSG00000100284	TOM1	-2,707532	0,00600538	0,04027804
ENSG00000030110	BAK1	-2,7072439	0,00522044	0,03682696
ENSG00000198355	PIM3	-2,6996107	8,9683E-05	0,00402422
ENSG00000141506	PIK3R5	-2,6994431	0,00600861	0,04027804
ENSG00000156671	SAMD8	-2,6948845	2,7608E-05	0,00243708
ENSG00000146457	WTAP	-2,6940655	0,00010862	0,0042889
ENSG00000272196	HIST2H2AA4	-2,6801845	0,00706165	0,04494127
ENSG00000139832	RAB20	-2,6641025	0,00137979	0,01619616
ENSG00000143622	RIT1	-2,6510089	0,00288927	0,02528543
ENSG00000180628	PCGF5	-2,6501131	0,00008536	0,00396422
ENSG00000128272	ATF4	-2,6466246	3,3257E-06	0,00105137

ENSG00000196843	ARID5A	-2,6398105	0,00015215	0,005035
ENSG00000020633	RUNX3	-2,6389185	0,0003005	0,00711948
ENSG00000140379	BCL2A1	-2,6344394	0,00347329	0,02845588
ENSG00000013441	CLK1	-2,633208	0,00020547	0,00585161
ENSG00000132819	RBM38	-2,6277567	0,00520311	0,03676816
ENSG00000110852	CLEC2B	-2,6164879	0,00648737	0,04249892
ENSG00000173812	EIF1	-2,6138885	3,4934E-05	0,00258691
ENSG00000239305	RNF103	-2,6048989	1,6121E-05	0,00195084
ENSG00000261740	BOLA2-SMG1P6	-2,603438	1,1826E-05	0,00172682
ENSG00000138166	DUSP5	-2,597417	0,00354307	0,02885405
ENSG00000168209	DDIT4	-2,5972149	0,00010116	0,00416329
ENSG00000025156	HSF2	-2,5851156	8,5907E-06	0,00152417
ENSG00000186660	ZFP91	-2,579018	0,00012003	0,00450047
ENSG00000090339	ICAM1	-2,5701898	4,4527E-06	0,00113545
ENSG00000108179	PPIF	-2,563852	0,00063541	0,01040807
ENSG00000144802	NFKBIZ	-2,5631017	0,00080858	0,01183711
ENSG00000155252	PI4K2A	-2,5584594	0,00015931	0,00516159
ENSG00000132952	USPL1	-2,5410932	0,00011966	0,00450045
ENSG00000117318	ID3	-2,5397202	0,00414193	0,03181836
ENSG00000228830	AL160408.2	-2,5305551	0,00343734	0,02825437
ENSG00000173575	CHD2	-2,5225793	1,9728E-05	0,00209417
ENSG00000128016	ZFP36	-2,5175055	1,8579E-06	0,00072361
ENSG00000086062	B4GALT1	-2,5172071	0,00106507	0,01396612
ENSG00000005379	TSPOAP1	-2,5162249	0,00570047	0,03890379
ENSG00000136603	SKIL	-2,515192	0,00467578	0,03435865
ENSG00000136950	ARPC5L	-2,5118218	0,00026344	0,0065383
ENSG00000165233	CARD19	-2,5089893	0,00351351	0,02872782
ENSG00000175130	MARCKSL1	-2,5082756	0,00365836	0,0293543
ENSG00000126524	SBDS	-2,5079443	2,7787E-05	0,00243708
ENSG00000171867	PRNP	-2,5063261	3,1033E-05	0,00247091
ENSG00000055483	USP36	-2,4915636	0,00406875	0,03144339
ENSG00000069956	MAPK6	-2,4890829	1,1972E-05	0,00172682
ENSG00000136738	STAM	-2,4888095	0,00120461	0,0149286
ENSG00000113742	CPEB4	-2,4886715	0,00021852	0,00605543
ENSG00000256235	SMIM3	-2,4813013	0,00043941	0,00849869
ENSG00000170345	FOS	-2,4797898	0,00134429	0,01593916
ENSG00000070495	JMJD6	-2,4786541	3,1498E-08	9,9367E-05
ENSG00000110721	CHKA	-2,4735609	0,00130225	0,01569382
ENSG00000114098	ARMC8	-2,4716311	0,00002934	0,00247091
ENSG00000140044	JDP2	-2,4644309	0,00023073	0,00618832
ENSG00000158050	DUSP2	-2,462857	0,00029188	0,00699359
ENSG00000120616	EPC1	-2,4584806	0,00033466	0,00750709

ENSG00000122862	SRGN	-2,4560601	2,4029E-05	0,00232537
ENSG00000123908	AGO2	-2,4547857	0,0065846	0,04293032
ENSG00000143384	MCL1	-2,4441334	0,00194474	0,01982826
ENSG00000145675	PIK3R1	-2,441854	0,0023946	0,02256253
ENSG00000078269	SYNJ2	-2,4373158	0,00598325	0,04016293
ENSG00000168264	IRF2BP2	-2,429963	0,00064653	0,01054802
ENSG00000137331	IER3	-2,4252383	0,00123386	0,01511267
ENSG00000130522	JUND	-2,4248099	0,00033001	0,00744788
ENSG00000183696	UPP1	-2,4172579	6,2311E-05	0,00345687
ENSG00000185728	YTHDF3	-2,4148913	3,4295E-05	0,00258668
ENSG00000137409	MTCH1	-2,4108513	0,00141461	0,01646306
ENSG00000079332	SAR1A	-2,4094834	0,00500256	0,03588879
ENSG00000102265	TIMP1	-2,4086179	9,1403E-05	0,00402538
ENSG00000100614	PPM1A	-2,394345	9,8995E-06	0,00158657
ENSG00000141580	WDR45B	-2,3922129	0,00067328	0,0108117
ENSG00000164823	OSGIN2	-2,3918182	2,4714E-05	0,00234909
ENSG00000176407	KCMF1	-2,389679	0,00097099	0,01322029
ENSG00000101558	VAPA	-2,3894321	0,00041469	0,00834066
ENSG00000165195	PIGA	-2,3874839	0,00042498	0,00843127
ENSG00000196428	TSC22D2	-2,3853791	5,9877E-06	0,00127673
ENSG00000122068	FYTTD1	-2,3851106	6,3208E-06	0,00130246
ENSG00000169251	NMD3	-2,3808098	8,8323E-05	0,00399188
ENSG00000150991	UBC	-2,3801737	0,00050444	0,00910856
ENSG00000120690	ELF1	-2,3757085	1,1868E-05	0,00172682
ENSG00000186162	CIDECP	-2,3749423	0,00019007	0,00562895
ENSG00000116741	RGS2	-2,3737744	0,00014258	0,0048558
ENSG00000213923	CSNK1E	-2,364823	0,0003246	0,00741101
ENSG00000171488	LRRC8C	-2,355966	0,00115187	0,01458483
ENSG00000140332	TLE3	-2,3545328	0,00306489	0,02615157
ENSG00000270681	AC095055.1	-2,3542244	9,1423E-05	0,00402538
ENSG00000068697	LAPTM4A	-2,3523167	0,00096277	0,0131815
ENSG00000121274	PAPD5	-2,3520944	0,00547955	0,03790244
ENSG00000152484	USP12	-2,3404817	0,00034941	0,00758211
ENSG00000234290	AC116366.1	-2,3380617	0,00540315	0,03763941
ENSG00000166165	CKB	-2,3311161	3,0882E-05	0,00247091
ENSG00000101421	CHMP4B	-2,3272269	0,00049382	0,00903649
ENSG00000132912	DCTN4	-2,3188327	0,00014025	0,0048288
ENSG00000118503	TNFAIP3	-2,3179108	0,00203299	0,02035782
ENSG00000147119	CHST7	-2,3162529	0,00076582	0,01154907
ENSG00000107937	GTPBP4	-2,3136002	0,00107913	0,01404534
ENSG00000272886	DCP1A	-2,3080158	1,7346E-09	2,1268E-05
ENSG00000082153	BZW1	-2,3026953	0,00129785	0,01567717
ENSG00000172216	CEBPB	-2,3021651	0,00185136	0,01919806

ENSG00000164211	STARD4	-2,300471	0,00523323	0,03687472
ENSG00000033327	GAB2	-2,3000569	0,00120573	0,0149286
ENSG00000112511	PHF1	-2,2944395	0,00802777	0,04890658
ENSG00000141551	CSNK1D	-2,2934417	0,00472347	0,0346054
ENSG00000083799	CYLD	-2,2931427	0,00011127	0,00430336
ENSG00000132326	PER2	-2,2884686	2,9883E-05	0,00247091
ENSG00000232956	SNHG15	-2,2853377	0,0023843	0,02250781
ENSG00000176946	THAP4	-2,281352	0,00272668	0,02448096
ENSG00000153066	TXNDC11	-2,2811407	0,00029918	0,00711168
ENSG00000196396	PTPN1	-2,279553	0,00407724	0,03146931
ENSG00000141232	TOB1	-2,2775336	0,00300804	0,02593533
ENSG00000176845	METRNL	-2,2703934	1,3273E-05	0,00177415
ENSG00000185947	ZNF267	-2,2670556	0,00026616	0,00657914
ENSG00000183735	TBK1	-2,2642713	3,629E-08	9,9367E-05
ENSG00000119801	YPEL5	-2,2635621	0,00120605	0,0149286
ENSG00000170852	KBTBD2	-2,2578628	0,00016862	0,00529405
ENSG00000131051	RBM39	-2,2573887	0,00025623	0,00644618
ENSG00000205423	CNEP1R1	-2,2529694	0,00370519	0,02959442
ENSG00000105856	HBP1	-2,2432646	1,6323E-06	0,00072361
ENSG00000125148	MT2A	-2,237605	0,00160791	0,01771402
ENSG00000101782	RIOK3	-2,2375008	0,0001606	0,00518852
ENSG00000105968	H2AFV	-2,2363222	0,00061544	0,01019874
ENSG00000138069	RAB1A	-2,2356487	0,00058729	0,00987721
ENSG00000135655	USP15	-2,232304	1,4667E-05	0,00188686
ENSG00000106635	BCL7B	-2,2308254	9,4275E-08	0,00016512
ENSG00000005812	FBXL3	-2,2248525	0,00038735	0,00800864
ENSG00000124782	RREB1	-2,2180143	0,00152731	0,01721445
ENSG00000162511	LAPTM5	-2,2173198	0,00592627	0,03991159
ENSG00000005483	KMT2E	-2,2143326	0,00330295	0,02749203
ENSG00000102034	ELF4	-2,2096423	0,00248492	0,0230981
ENSG00000171988	JMJD1C	-2,2060666	6,9035E-05	0,00360489
ENSG00000151553	FAM160B1	-2,2005891	8,6546E-05	0,00398162
ENSG00000160789	LMNA	-2,2003875	0,00133985	0,0159333
ENSG00000184007	PTP4A2	-2,198067	0,0001128	0,00434893
ENSG00000182831	C16orf72	-2,1978721	0,00059956	0,01001484
ENSG00000146232	NFKBIE	-2,1936715	0,00010427	0,00422125
ENSG00000006607	FARP2	-2,1883606	0,00024109	0,00627852
ENSG00000044574	HSPA5	-2,1867724	0,00329351	0,02744928
ENSG00000092820	EZR	-2,1817306	0,00337019	0,02789077
ENSG00000115520	COQ10B	-2,1772177	7,5037E-05	0,00373753
ENSG00000260708	AL118516.1	-2,1736574	7,6062E-05	0,00374838
ENSG00000182149	IST1	-2,1692753	1,7594E-06	0,00072361
ENSG00000137817	PARP6	-2,1688117	0,00114274	0,01456394

ENSG00000164674	SYTL3	-2,1676364	0,0074625	0,04666828
ENSG00000134758	RNF138	-2,1645196	0,00019202	0,00564574
ENSG00000130803	ZNF317	-2,1600237	7,1528E-06	0,00139049
ENSG00000177426	TGIF1	-2,1553759	0,00037358	0,00782615
ENSG00000156535	CD109	-2,1546015	0,0004598	0,00873341
ENSG00000179094	PER1	-2,1537697	0,00101636	0,01359632
ENSG00000163961	RNF168	-2,1499481	0,00030598	0,0071693
ENSG00000163877	SNIP1	-2,1482308	9,8193E-05	0,00412294
ENSG00000112245	PTP4A1	-2,1461397	0,00304256	0,02604765
ENSG00000117569	PTBP2	-2,1458648	0,00253458	0,02336483
ENSG00000025772	TOMM34	-2,145201	0,00079147	0,01171957
ENSG00000169826	CSGALNACT2	-2,1432393	0,00001423	0,00185609
ENSG00000185477	GPRIN3	-2,1386978	1,0875E-07	0,00016726
ENSG00000179361	ARID3B	-2,1373472	0,00013508	0,00477951
ENSG00000134480	CCNH	-2,1351455	0,00339487	0,02798173
ENSG00000006451	RALA	-2,1337589	0,0016652	0,01804342
ENSG00000135093	USP30	-2,1307402	0,00766592	0,04743277
ENSG00000149658	YTHDF1	-2,1250147	1,3862E-06	0,00070816
ENSG00000154710	RABGEF1	-2,1241169	0,00722388	0,0456538
ENSG00000130202	NECTIN2	-2,123706	0,0069477	0,04444678
ENSG00000263020	AL662899.2	-2,1191539	7,6267E-05	0,00374838
ENSG00000111011	RSRC2	-2,1167826	3,9491E-06	0,00111306
ENSG00000155304	HSPA13	-2,1139615	2,6476E-06	0,0009144
ENSG00000116044	NFE2L2	-2,1117551	0,0001582	0,00515843
ENSG00000145780	FEM1C	-2,1067878	0,00649791	0,04252345
ENSG00000197063	MAFG	-2,1067368	3,5441E-06	0,00108631
ENSG00000083937	CHMP2B	-2,1058028	1,4845E-05	0,00188736
ENSG00000140941	MAP1LC3B	-2,1050437	9,1727E-05	0,00403089
ENSG00000143702	CEP170	-2,1045508	0,00244717	0,02289341
ENSG00000132823	OSER1	-2,1017848	0,0002765	0,00669315
ENSG00000114120	SLC25A36	-2,099683	0,00007293	0,0036936
ENSG00000100401	RANGAP1	-2,0993948	0,00106151	0,01392682
ENSG00000161011	SQSTM1	-2,0886163	1,7665E-06	0,00072361
ENSG00000117000	RLF	-2,0859921	5,6293E-05	0,00328822
ENSG00000173349	SFT2D3	-2,08517	3,7546E-05	0,00260074
ENSG00000174574	AKIRIN1	-2,0834098	3,4495E-05	0,00258668
ENSG00000171310	CHST11	-2,0808294	0,00151149	0,01707982
ENSG00000172845	SP3	-2,0791592	5,4418E-07	0,00047656
ENSG00000138750	NUP54	-2,0755067	0,00010725	0,00425732
ENSG00000139826	ABHD13	-2,072781	1,1253E-06	0,00063084
ENSG00000106610	STAG3L4	-2,066698	0,00036494	0,00772987
ENSG00000117139	KDM5B	-2,0652782	0,00659469	0,04296183
ENSG00000135404	CD63	-2,0559971	0,004084	0,03150736

ENSG00000197619	ZNF615	-2,0552613	0,0038289	0,0302281
ENSG00000153914	SREK1	-2,0547499	0,00017427	0,00540231
ENSG00000114796	KLHL24	-2,0470183	0,0019059	0,01961168
ENSG00000023734	STRAP	-2,0437329	0,00164543	0,01790842
ENSG00000111832	RWDD1	-2,0430504	0,0012627	0,01532809
ENSG00000139572	GPR84	-2,0428968	0,00725546	0,04577079
ENSG00000109787	KLF3	-2,0384561	0,00013609	0,00479465
ENSG00000115816	CEBPZ	-2,037684	6,2004E-05	0,00344763
ENSG00000143751	SDE2	-2,0372621	5,5253E-06	0,00124298
ENSG00000267520	AC010733.2	-2,031499	7,0174E-05	0,00362046
ENSG00000051108	HERPUD1	-2,0311363	5,6174E-05	0,00328822
ENSG00000109332	UBE2D3	-2,0311111	0,00035349	0,00764037
ENSG00000121671	CRY2	-2,0302442	0,00190733	0,01961816
ENSG00000143761	ARF1	-2,0295856	8,7079E-05	0,00399188
ENSG00000143153	ATP1B1	-2,0288151	0,00032286	0,00738511
ENSG00000184182	UBE2F	-2,0263867	0,00023071	0,00618832
ENSG00000159128	IFNGR2	-2,0254313	0,000427	0,00843962
ENSG00000239857	GET4	-2,0251793	4,7721E-06	0,00116551
ENSG00000085433	WDR47	-2,0250441	0,00192267	0,01968505
ENSG00000107263	RAPGEF1	-2,023814	0,00473279	0,03462596
ENSG00000019995	ZRANB1	-2,0233393	0,00036565	0,00772987
ENSG00000145241	CENPC	-2,0232326	0,00420615	0,03211049
ENSG00000091317	CMTM6	-2,0221887	0,00028177	0,00678051
ENSG00000115548	KDM3A	-2,0199995	0,00047633	0,00885519
ENSG00000135241	PNPLA8	-2,0196379	0,00095777	0,0131616
ENSG00000144597	EAF1	-2,0179009	9,3837E-05	0,00405102
ENSG00000279766	AC067931.1	-2,0172766	0,00116658	0,01467708
ENSG00000110046	ATG2A	-2,0099996	0,00705654	0,04492697
ENSG00000117410	ATP6V0B	-2,0093181	3,5244E-05	0,00258691
ENSG00000205189	ZBTB10	-2,0077124	6,2508E-05	0,00345998
ENSG00000158122	AAED1	-2,0076846	0,0004963	0,00906014
ENSG00000156273	BACH1	-2,0067723	0,00070176	0,01108048
ENSG00000100393	EP300	-2,0047207	5,6321E-05	0,00328822
ENSG00000114013	CD86	-2,0034798	0,0015014	0,01702067
ENSG00000160570	DEDD2	-2,0010766	0,00009588	0,0040683
ENSG00000069399	BCL3	-1,9952345	7,6222E-05	0,00374838
ENSG00000137947	GTF2B	-1,9938134	2,8517E-05	0,00247091
ENSG00000150977	RILPL2	-1,9931871	0,00443688	0,03318999
ENSG00000171604	CXXC5	-1,9927001	0,00046609	0,0087915
ENSG00000163660	CCNL1	-1,9921012	0,00199872	0,02016072
ENSG00000124688	MAD2L1BP	-1,9919107	0,00117061	0,01472033
ENSG00000116560	SFPQ	-1,98941	1,132E-06	0,00063084
ENSG00000266094	RASSF5	-1,987248	0,0003844	0,00798125

ENSG00000163874	ZC3H12A	-1,9863859	0,00013008	0,00471742
ENSG00000086666	ZFAND6	-1,9833031	0,00010379	0,00421955
ENSG00000137309	HMGA1	-1,9823649	0,00148099	0,01690807
ENSG00000165006	UBAP1	-1,9794182	0,00094539	0,01305407
ENSG00000104969	SGTA	-1,9783205	0,00018231	0,00553267
ENSG00000122257	RBBP6	-1,9756874	0,00030178	0,00713546
ENSG00000165806	CASP7	-1,9727297	0,00029205	0,00699359
ENSG00000160741	CRTC2	-1,9696444	0,00421073	0,03211885
ENSG00000124226	RNF114	-1,9665901	4,8957E-06	0,00116551
ENSG00000100425	BRD1	-1,9661507	0,00154947	0,01734116
ENSG00000124201	ZNFX1	-1,9658692	0,00014703	0,00494973
ENSG00000104450	SPAG1	-1,9631236	0,00079623	0,0117405
ENSG00000170779	CDCA4	-1,9617448	0,00152838	0,01721521
ENSG00000143514	TP53BP2	-1,9610361	0,0013878	0,01624933
ENSG00000132475	H3F3B	-1,9591803	0,00049177	0,00901549
ENSG00000140367	UBE2Q2	-1,9563624	8,5912E-05	0,00396731
ENSG00000133606	MKRN1	-1,9549911	3,0783E-05	0,00247091
ENSG00000178381	ZFAND2A	-1,9531504	0,00026682	0,0065821
ENSG00000140455	USP3	-1,9501203	0,00502428	0,03597612
ENSG00000104064	GABPB1	-1,9471738	0,00039587	0,00808929
ENSG00000159346	ADIPOR1	-1,9443994	4,0613E-05	0,00275867
ENSG00000109670	FBXW7	-1,9427164	0,00046952	0,00880143
ENSG00000166822	TMEM170A	-1,9424891	1,6983E-08	7,9645E-05
ENSG00000162924	REL	-1,9401396	0,00026966	0,00661576
ENSG00000076053	RBM7	-1,9379193	0,00013044	0,00471742
ENSG00000095574	IKZF5	-1,9370463	0,00018585	0,00554421
ENSG00000171522	PTGER4	-1,9349875	0,00105331	0,0138991
ENSG00000175606	TMEM70	-1,9339488	0,00043356	0,00847119
ENSG00000187522	HSPA14	-1,9329154	2,3788E-05	0,00232537
ENSG00000113615	SEC24A	-1,9321413	0,00146432	0,01683258
ENSG00000116670	MAD2L2	-1,9319801	0,00024433	0,00629335
ENSG00000102580	DNAJC3	-1,9303562	8,7282E-05	0,00399188
ENSG00000078804	TP53INP2	-1,9300858	5,1588E-05	0,00316792
ENSG00000283154	IQCJ-SCHIP1	-1,9285495	0,00315838	0,02662668
ENSG00000156875	MFSD14A	-1,927556	7,6062E-06	0,00141771
ENSG00000280138	AC027290.2	-1,9273757	0,00061416	0,0101963
ENSG00000008294	SPAG9	-1,9270071	0,00153876	0,01728443
ENSG00000166200	COPS2	-1,9253271	2,9176E-05	0,00247091
ENSG00000101544	ADNP2	-1,9250302	0,00304443	0,02604765
ENSG00000173846	PLK3	-1,9230511	1,0914E-07	0,00016726
ENSG00000162664	ZNF326	-1,9226518	4,5154E-06	0,00113545
ENSG00000167491	GATAD2A	-1,9226206	0,00140666	0,01638797
ENSG00000162616	DNAJB4	-1,9225243	3,6924E-05	0,00258691

ENSG00000091527	CDV3	-1,9211819	0,00187522	0,01936096
ENSG00000196646	ZNF136	-1,9206159	0,00076302	0,01152089
ENSG00000165650	PDZD8	-1,9181506	0,00164997	0,01794985
ENSG00000027697	IFNGR1	-1,9176802	4,4224E-06	0,00113545
ENSG00000169018	FEM1B	-1,9155893	0,00203857	0,0203948
ENSG00000101150	TPD52L2	-1,9151767	0,00825724	0,04980954
ENSG00000196233	LCOR	-1,9144308	0,00013188	0,00472799
ENSG00000121749	TBC1D15	-1,9116716	4,4102E-06	0,00113545
ENSG00000069849	ATP1B3	-1,905183	0,00561832	0,03853627
ENSG00000112137	PHACTR1	-1,9030696	9,0759E-06	0,0015563
ENSG00000070540	WIPI1	-1,8995384	0,00610367	0,040691
ENSG00000224531	SMIM13	-1,8989	0,00460264	0,03397391
ENSG00000121741	ZMYM2	-1,8973386	0,00023054	0,00618832
ENSG00000067064	IDI1	-1,896822	0,00034395	0,00753305
ENSG00000125037	EMC3	-1,8962576	0,00027441	0,00665886
ENSG00000111615	KRR1	-1,8958842	0,00013257	0,00473877
ENSG00000269968	AC006064.4	-1,8953496	0,00096458	0,0131989
ENSG00000166669	ATF7IP2	-1,8914247	0,00394007	0,03080821
ENSG00000125651	GTF2F1	-1,8903177	1,3929E-05	0,00183637
ENSG00000189376	C8orf76	-1,8894899	0,00038043	0,00791888
ENSG00000163788	SNRK	-1,8893194	0,00011611	0,00444169
ENSG00000119523	ALG2	-1,8879524	0,00027052	0,00662029
ENSG00000126775	ATG14	-1,8878052	0,00054084	0,00944078
ENSG00000105193	RPS16	-1,8876881	0,00451693	0,03355334
ENSG00000156232	WHAMM	-1,8860028	0,00044085	0,00851842
ENSG00000180611	MB21D2	-1,8852915	0,00018132	0,00551626
ENSG00000065809	FAM107B	-1,8852689	0,0014047	0,01637887
ENSG00000116786	PLEKHM2	-1,8838202	0,00542316	0,03769313
ENSG00000181555	SETD2	-1,8830817	0,00084727	0,01212123
ENSG00000169682	SPNS1	-1,8828497	0,00107785	0,01404353
ENSG00000008056	SYN1	-1,8814414	0,00472236	0,0346054
ENSG00000130734	ATG4D	-1,8813276	1,7341E-06	0,00072361
ENSG00000137876	RSL24D1	-1,8804316	0,00054598	0,00951443
ENSG00000099860	GADD45B	-1,8752665	0,00385345	0,0303535
ENSG00000135018	UBQLN1	-1,8746454	0,00055505	0,00959146
ENSG00000056972	TRAF3IP2	-1,873637	0,00014084	0,0048288
ENSG00000124145	SDC4	-1,870902	0,00018193	0,00552794
ENSG00000160799	CCDC12	-1,8692001	2,5576E-05	0,00235489
ENSG00000153922	CHD1	-1,8686646	1,1098E-06	0,00063084
ENSG00000280254	AC233723.2	-1,8668753	0,00225305	0,02169954
ENSG00000121879	PIK3CA	-1,8658033	0,00592383	0,03990612
ENSG00000131263	RLIM	-1,8646344	0,00082311	0,01197831
ENSG00000197872	FAM49A	-1,8634608	0,00056969	0,00974833

ENSG00000164169	PRMT9	-1,8633177	1,7294E-05	0,0019819
ENSG00000162910	MRPL55	-1,8623997	0,00580486	0,03938384
ENSG00000184014	DENND5A	-1,8610079	0,00435655	0,03273891
ENSG00000109113	RAB34	-1,8596354	3,4066E-05	0,00258042
ENSG00000100225	FBXO7	-1,8586611	6,7845E-05	0,0035987
ENSG00000104267	CA2	-1,8574431	0,00153379	0,01726026
ENSG00000113811	SELENOK	-1,856016	0,00106898	0,01400238
ENSG00000057704	TMCC3	-1,8536734	0,00274785	0,02459124
ENSG00000172062	SMN1	-1,8510165	0,00159167	0,0175966
ENSG00000128524	ATP6V1F	-1,8491847	3,9417E-06	0,00111306
ENSG00000141753	IGFBP4	-1,8470212	0,00315385	0,02661422
ENSG00000165782	PIP4P1	-1,84491	0,00203353	0,02035782
ENSG00000168066	SF1	-1,8425982	0,00647376	0,04243329
ENSG00000230551	AC021078.1	-1,8401631	0,00050427	0,00910856
ENSG00000136732	GYPC	-1,8359854	0,00045059	0,00860502
ENSG00000089737	DDX24	-1,8349212	0,00016643	0,00527776
ENSG00000151247	EIF4E	-1,8344615	0,00010678	0,00425732
ENSG00000180228	PRKRA	-1,8339559	7,3975E-07	0,00056685
ENSG00000059769	DNAJC25	-1,8325861	0,00787315	0,0483544
ENSG00000158615	PPP1R15B	-1,8301027	0,00045054	0,00860502
ENSG00000134108	ARL8B	-1,8298506	0,00116021	0,01462124
ENSG00000163605	PPP4R2	-1,829435	0,00011931	0,00449414
ENSG00000143771	CNIH4	-1,8289985	0,00208569	0,02064588
ENSG00000173960	UBXN2A	-1,8276751	0,0003896	0,00804148
ENSG00000188229	TUBB4B	-1,8272672	0,00112149	0,01438282
ENSG00000241839	PLEKHO2	-1,8267147	1,9994E-05	0,00209514
ENSG00000168883	USP39	-1,8244239	1,9428E-05	0,00209417
ENSG00000108175	ZMIZ1	-1,8238276	0,00045941	0,00873341
ENSG00000067334	DNTTIP2	-1,8238099	0,00120071	0,01491516
ENSG00000183484	GPR132	-1,8235978	0,00567584	0,03881743
ENSG00000089818	NECAP1	-1,8218209	0,00018447	0,00554421
ENSG00000179119	SPTY2D1	-1,8213693	0,00245135	0,02289341
ENSG00000065548	ZC3H15	-1,8192952	0,00035458	0,00764037
ENSG00000070501	POLB	-1,8181216	0,00048623	0,00895117
ENSG00000072110	ACTN1	-1,8179557	0,00499961	0,03587808
ENSG00000115339	GALNT3	-1,8165794	0,00045128	0,00861151
ENSG00000124766	SOX4	-1,8142482	0,00447135	0,03333599
ENSG00000116752	BCAS2	-1,8140817	0,00009355	0,00404623
ENSG00000111640	GAPDH	-1,8130592	9,7557E-05	0,00410899
ENSG00000101216	GMEB2	-1,8095024	0,00018457	0,00554421
ENSG00000084463	WBP11	-1,8092926	8,8763E-07	0,00058408
ENSG00000184203	PPP1R2	-1,809088	6,3197E-06	0,00130246
ENSG00000196235	SUPT5H	-1,8076369	0,00109886	0,01422121

ENSG00000055208	TAB2	-1,8062379	0,00343832	0,02825437
ENSG00000113575	PPP2CA	-1,8044306	1,8608E-05	0,00205537
ENSG00000167004	PDIA3	-1,8038034	0,00012177	0,00452563
ENSG00000267519	AC020916.1	-1,7998897	0,00149693	0,01700953
ENSG00000182481	KPNA2	-1,7993866	0,00034453	0,00753632
ENSG00000154124	OTULIN	-1,7983195	0,0002233	0,00614267
ENSG00000204977	TRIM13	-1,7978517	1,8591E-06	0,00072361
ENSG00000139505	MTMR6	-1,79779	0,00036179	0,00772096
ENSG00000104765	BNIP3L	-1,7977636	0,00034129	0,00753305
ENSG00000106615	RHEB	-1,7962902	0,00059722	0,00998656
ENSG00000136802	LRRC8A	-1,7951508	0,00090018	0,01263658
ENSG00000170540	ARL6IP1	-1,7943216	0,00011055	0,00430067
ENSG00000123066	MED13L	-1,7929845	0,00112148	0,01438282
ENSG00000057757	PITHD1	-1,7924197	0,00022859	0,00618155
ENSG00000115956	PLEK	-1,7917007	0,00226462	0,02176824
ENSG00000168092	PAFAH1B2	-1,7895901	4,3281E-05	0,00284526
ENSG00000139725	RHOF	-1,7882095	7,4882E-06	0,00141771
ENSG00000138670	RASGEF1B	-1,7858155	0,00244992	0,02289341
ENSG00000111647	UHRF1BP1L	-1,7857895	7,2017E-06	0,00139049
ENSG00000198431	TXNRD1	-1,7766712	0,00008881	0,00399582
ENSG00000153201	RANBP2	-1,7762647	0,0032495	0,02714851
ENSG00000070831	CDC42	-1,7755383	1,1149E-05	0,00167723
ENSG00000163694	RBM47	-1,7748066	0,0001073	0,00425732
ENSG00000082515	MRPL22	-1,7705954	0,0002366	0,00623987
ENSG00000196704	AMZ2	-1,7691896	0,00651042	0,04259391
ENSG00000132661	NXT1	-1,7687846	0,00249973	0,023183
ENSG00000081320	STK17B	-1,7639004	4,6843E-05	0,00299126
ENSG00000054523	KIF1B	-1,7630675	0,00385884	0,0303667
ENSG00000168175	MAPK1IP1L	-1,7600731	0,00014942	0,0049783
ENSG00000205937	RNPS1	-1,7596979	0,00023666	0,00623987
ENSG00000162702	ZNF281	-1,7590484	0,00062443	0,01027881
ENSG00000115540	MOB4	-1,7574505	5,1677E-05	0,00316792
ENSG00000136826	KLF4	-1,7541843	0,00084466	0,01210519
ENSG00000104763	ASAH1	-1,7491634	0,0009309	0,01292339
ENSG00000162928	PEX13	-1,749129	3,7579E-06	0,00109843
ENSG00000225648	SBDSP1	-1,7468889	2,7661E-06	0,00094205
ENSG00000105821	DNAJC2	-1,7452263	0,00106696	0,01398339
ENSG00000146872	TLK2	-1,7439484	0,00030205	0,00713546
ENSG00000198833	UBE2J1	-1,7408017	0,00506701	0,03611864
ENSG00000054967	RELT	-1,7406158	0,00082301	0,01197831
ENSG00000106952	TNFSF8	-1,7403975	0,00395369	0,03086549
ENSG00000161526	SAP30BP	-1,7378564	0,00029422	0,0070385
ENSG00000160218	TRAPPC10	-1,7360795	0,00184837	0,01919806

ENSG00000111252	SH2B3	-1,7352715	0,00235105	0,02231037
ENSG00000138081	FBXO11	-1,7347254	6,1688E-05	0,00343784
ENSG00000197019	SERTAD1	-1,7339951	1,1573E-05	0,00172682
ENSG00000004897	CDC27	-1,7327003	0,00006437	0,00348437
ENSG00000112033	PPARD	-1,7323666	0,00758703	0,047123
ENSG00000119977	TCTN3	-1,7317186	0,00115604	0,01460451
ENSG00000157954	WIPI2	-1,7287099	0,0002805	0,00675657
ENSG00000138433	CIR1	-1,7280964	0,00069052	0,01093809
ENSG00000065526	SPEN	-1,7276894	4,1756E-05	0,00278803
ENSG00000101247	NDUFAF5	-1,7270455	0,00026251	0,00652636
ENSG00000106346	USP42	-1,7237896	4,8657E-05	0,00304437
ENSG00000119231	SENP5	-1,7206753	0,00089807	0,01262081
ENSG00000080546	SESN1	-1,7172725	0,00170466	0,01830649
ENSG00000113013	HSPA9	-1,7163892	0,00055963	0,00965489
ENSG00000171222	SCAND1	-1,716169	6,1576E-05	0,00343784
ENSG00000137393	RNF144B	-1,7147283	0,00596577	0,04008951
ENSG00000241978	AKAP2	-1,7140075	0,00179238	0,01884348
ENSG00000132155	RAF1	-1,7131307	0,00057625	0,00980887
ENSG00000160908	ZNF394	-1,7117536	0,00194298	0,01982826
ENSG00000089693	MLF2	-1,7114292	2,0728E-05	0,00215374
ENSG00000165527	ARF6	-1,7099215	0,00042303	0,00842663
ENSG00000151239	TWF1	-1,7097674	0,00286803	0,02518874
ENSG00000111711	GOLT1B	-1,7079327	1,2642E-05	0,00175782
ENSG00000099968	BCL2L13	-1,7073099	0,00153547	0,01727121
ENSG00000185359	HGS	-1,7056297	0,00099723	0,013458
ENSG00000196850	PPTC7	-1,7053864	5,8164E-06	0,00126265
ENSG00000070756	PABPC1	-1,7035992	0,00111104	0,01432376
ENSG00000105851	PIK3CG	-1,702099	0,00091801	0,01280457
ENSG00000021574	SPAST	-1,6994598	9,1393E-05	0,00402538
ENSG00000129351	ILF3	-1,6941141	0,00576926	0,03923132
ENSG00000147872	PLIN2	-1,6933619	0,00011509	0,00440969
ENSG00000023287	RB1CC1	-1,6930407	0,00099999	0,01346588
ENSG00000215717	TMEM167B	-1,6928171	4,0502E-06	0,00112858
ENSG00000103978	TMEM87A	-1,6873027	0,00175798	0,01863617
ENSG00000135956	TMEM127	-1,6870198	0,00076207	0,01151365
ENSG00000181467	RAP2B	-1,6868173	0,00170236	0,01830058
ENSG00000107372	ZFAND5	-1,6852259	0,00016391	0,00524714
ENSG00000183876	ARSI	-1,6839177	0,00157749	0,01750301
ENSG00000198160	MIER1	-1,6838008	2,9236E-05	0,00247091
ENSG00000170185	USP38	-1,6834352	0,00070256	0,01108048
ENSG00000114126	TFDP2	-1,6831053	0,00017381	0,00539499
ENSG00000067182	TNFRSF1A	-1,6798694	0,00111436	0,01433644
ENSG00000197021	CXorf40B	-1,6774724	0,00068791	0,01092021

ENSG00000114125	RNF7	-1,6769504	0,00041096	0,00829391
ENSG00000048405	ZNF800	-1,6754336	1,1026E-05	0,0016769
ENSG00000155330	C16orf87	-1,6752709	0,00453358	0,03360575
ENSG00000120727	PAIP2	-1,6744342	5,1802E-10	1,2702E-05
ENSG00000163661	PTX3	-1,6735087	0,00019931	0,00575641
ENSG00000117614	SYF2	-1,6734453	9,1437E-05	0,00402538
ENSG00000133773	CCDC59	-1,6712181	0,00046115	0,00874028
ENSG00000147526	TACC1	-1,670705	0,00361087	0,02913529
ENSG00000137449	CPEB2	-1,6703251	9,9125E-05	0,00413489
ENSG00000144566	RAB5A	-1,6681798	5,9378E-05	0,00338854
ENSG00000123562	MORF4L2	-1,6651618	0,00019502	0,00570755
ENSG00000217128	FNIP1	-1,6643642	0,00359671	0,02909762
ENSG00000166503	HDGFL3	-1,6641001	3,5278E-05	0,00258691
ENSG00000146676	PURB	-1,6635264	0,00595242	0,04002171
ENSG00000138032	PPM1B	-1,6625842	0,00314484	0,02660039
ENSG00000196470	SIAH1	-1,6625181	0,00816077	0,04944294
ENSG00000198612	COPS8	-1,6618427	0,00372146	0,0296954
ENSG00000172766	NAA16	-1,6616869	0,00125398	0,0152751
ENSG00000067082	KLF6	-1,6588447	1,4653E-06	0,00071859
ENSG00000106829	TLE4	-1,6566475	0,00061631	0,01020433
ENSG00000142227	EMP3	-1,6557176	2,1409E-05	0,00219518
ENSG00000083896	YTHDC1	-1,6532322	0,00058671	0,00987721
ENSG00000115738	ID2	-1,6511283	0,00063253	0,01036781
ENSG00000015479	MATR3	-1,6492457	5,3137E-06	0,00121772
ENSG00000144747	TMF1	-1,6473253	0,00310324	0,02634646
ENSG00000022840	RNF10	-1,6468618	0,00041023	0,008286
ENSG00000279602	AC109326.1	-1,6461877	0,00490367	0,03538639
ENSG00000148344	PTGES	-1,6440044	0,00054067	0,00944078
ENSG00000215251	FASTKD5	-1,6432355	0,00309637	0,02629927
ENSG00000100083	GGA1	-1,6423568	0,00018258	0,0055339
ENSG00000143774	GUK1	-1,6421811	0,00201586	0,02023368
ENSG00000072364	AFF4	-1,6400133	3,6274E-06	0,00109812
ENSG00000251022	THAP9-AS1	-1,6393295	0,00643178	0,04222591
ENSG00000075415	SLC25A3	-1,6386383	0,00213686	0,02096761
ENSG00000173875	ZNF791	-1,6379764	0,00055063	0,00954101
ENSG00000105993	DNAJB6	-1,6377509	0,00011084	0,00430067
ENSG00000133134	BEX2	-1,6366951	0,0035304	0,02880826
ENSG00000171612	SLC25A33	-1,6365563	0,00037109	0,00780397
ENSG00000117155	SSX2IP	-1,6351118	0,00022277	0,00613776
ENSG00000106546	AHR	-1,6343365	0,0004623	0,00874028
ENSG00000111897	SERINC1	-1,6323158	0,00013278	0,00473933
ENSG00000137815	RTF1	-1,6308239	0,00061743	0,01020955
ENSG00000080822	CLDND1	-1,630272	0,00008856	0,00399188

ENSG00000159216	RUNX1	-1,6288137	0,00073177	0,01130557
ENSG00000009307	CSDE1	-1,6285677	0,00001273	0,00175782
ENSG00000124214	STAU1	-1,6276452	6,0965E-05	0,00342088
ENSG00000165714	BORCS5	-1,6272762	0,002772	0,02468125
ENSG00000170776	AKAP13	-1,6264214	0,00148196	0,01690807
ENSG00000278311	GGNBP2	-1,6255304	0,0008554	0,01218005
ENSG00000105849	TWISTNB	-1,6229385	0,00186054	0,01924991
ENSG00000173276	ZBTB21	-1,6222779	0,00035435	0,00764037
ENSG00000173020	GRK2	-1,6220951	0,00024664	0,00633939
ENSG00000162783	IER5	-1,6213869	0,00012539	0,0046099
ENSG00000204103	MAFB	-1,6209133	0,00171397	0,0183567
ENSG00000094975	SUCO	-1,620635	0,00200437	0,02018445
ENSG00000152700	SAR1B	-1,6196923	4,5379E-06	0,00113545
ENSG00000224032	EPB41L4A-AS1	-1,6180491	8,1626E-05	0,00390836
ENSG00000141985	SH3GL1	-1,6164306	0,00234017	0,0222502
ENSG00000136807	CDK9	-1,6153714	0,00229623	0,02200305
ENSG00000100221	JOSD1	-1,6140052	0,00296886	0,02572414
ENSG00000128271	ADORA2A	-1,6125313	0,00056546	0,00969626
ENSG00000089234	BRAP	-1,6123065	0,00018673	0,0055635
ENSG00000103121	CMC2	-1,6114033	0,00676338	0,04367787
ENSG00000090061	CCNK	-1,6090458	0,00031452	0,00728247
ENSG00000116030	SUMO1	-1,6080789	3,6753E-05	0,00258691
ENSG00000140153	WDR20	-1,6057338	0,00030788	0,00719047
ENSG00000102225	CDK16	-1,6052522	0,00489624	0,03534952
ENSG00000141428	C18orf21	-1,6039097	0,00057043	0,00975424
ENSG00000269220	LINC00528	-1,6037884	0,00311582	0,02640052
ENSG00000183624	HMCES	-1,6032051	2,8923E-05	0,00247091
ENSG00000107341	UBE2R2	-1,60101	0,00103665	0,01374929
ENSG00000163811	WDR43	-1,6006509	0,00024372	0,00629335
ENSG00000177374	HIC1	-1,6004999	0,00266936	0,02419588
ENSG00000137492	THAP12	-1,6004513	3,3496E-05	0,00257887
ENSG00000146425	DYNLT1	-1,5999791	0,00056261	0,00966225
ENSG00000104626	ERI1	-1,5999432	0,00824111	0,04973783
ENSG00000119048	UBE2B	-1,5993423	8,302E-06	0,00149686
ENSG00000170385	SLC30A1	-1,5985009	0,00027204	0,00664527
ENSG00000122482	ZNF644	-1,5957031	0,00179454	0,01885348
ENSG00000150787	PTS	-1,5953152	0,00615819	0,04087169
ENSG00000177879	AP3S1	-1,5948509	0,00360524	0,02910899
ENSG00000182957	SPATA13	-1,5924152	0,00179225	0,01884348
ENSG00000101367	MAPRE1	-1,5923949	0,0016547	0,0179623
ENSG00000047634	SCML1	-1,5915689	0,00432178	0,03263973
ENSG00000110713	NUP98	-1,5900196	5,4925E-05	0,00326105

ENSG00000214176	PLEKHM1P1	-1,5896179	0,00301926	0,0259955
ENSG00000135269	TES	-1,5880541	0,00024962	0,00637054
ENSG00000198369	SPRED2	-1,5880513	0,0028475	0,02508031
ENSG00000119725	ZNF410	-1,5878749	0,0010975	0,01422121
ENSG00000174437	ATP2A2	-1,5877555	0,0004964	0,00906014
ENSG00000132510	KDM6B	-1,5863382	0,00033292	0,00749123
ENSG00000143256	PFDN2	-1,5856519	0,00107715	0,01404353
ENSG00000157540	DYRK1A	-1,5853711	0,00118608	0,01481413
ENSG00000149806	FAU	-1,584453	0,00011991	0,00450047
ENSG00000119899	SLC17A5	-1,5811686	3,6743E-05	0,00258691
ENSG00000110367	DDX6	-1,5792661	0,00023117	0,00618832
ENSG00000178623	GPR35	-1,5789416	0,006557	0,04279587
ENSG00000144228	SPOPL	-1,578151	0,00082589	0,01199033
ENSG00000137177	KIF13A	-1,577648	1,7293E-05	0,0019819
ENSG00000197579	TOPORS	-1,5775088	0,00073457	0,01131434
ENSG00000197780	TAF13	-1,5764018	0,00013428	0,00477892
ENSG00000107560	RAB11FIP2	-1,5761879	0,00173135	0,01845047
ENSG00000178951	ZBTB7A	-1,5758716	0,00056147	0,00965489
ENSG00000108100	CCNY	-1,5749568	0,00374792	0,02984824
ENSG00000196352	CD55	-1,5728941	0,00089224	0,01258117
ENSG00000163125	RPRD2	-1,5709714	0,00150894	0,0170667
ENSG00000109606	DHX15	-1,5703117	3,0392E-05	0,00247091
ENSG00000213079	SCAF8	-1,5701087	0,0004743	0,00883761
ENSG00000134153	EMC7	-1,5692563	2,9486E-05	0,00247091
ENSG00000170881	RNF139	-1,5656968	0,00763541	0,04732757
ENSG00000124198	ARFGEF2	-1,5654846	0,00325136	0,02715483
ENSG00000173457	PPP1R14B	-1,5648761	0,00094723	0,01305626
ENSG00000102753	KPNA3	-1,5643338	1,0829E-06	0,00063084
ENSG00000089902	RCOR1	-1,5619388	0,0001514	0,00503033
ENSG00000083828	ZNF586	-1,5615407	0,00103788	0,01374929
ENSG00000242125	SNHG3	-1,558513	1,5503E-05	0,00191997
ENSG00000140299	BNIP2	-1,558468	1,6162E-05	0,00195084
ENSG00000112242	E2F3	-1,5583594	0,0001338	0,00476863
ENSG00000169641	LUZP1	-1,5569554	0,00704146	0,04487102
ENSG00000143217	NECTIN4	-1,5563837	0,00241062	0,02266191
ENSG00000106608	URGCP	-1,5519776	5,4905E-06	0,00124298
ENSG00000068745	IP6K2	-1,5514366	7,2373E-05	0,00367423
ENSG00000267165	CHMP1B-AS1	-1,5502047	0,00074602	0,01140723
ENSG00000173039	RELA	-1,5488635	0,0013765	0,01618283
ENSG00000121797	CCRL2	-1,54804	0,00011075	0,00430067
ENSG00000139620	KANSL2	-1,5479633	0,00022196	0,00612234
ENSG00000178127	NDUFV2	-1,5469243	2,4274E-06	0,00086264
ENSG00000151532	VTI1A	-1,5466598	0,0003943	0,00807433

ENSG00000026508	CD44	-1,5460159	0,00537649	0,03751369
ENSG00000104081	BMF	-1,5454684	0,00097398	0,01323891
ENSG00000125835	SNRPB	-1,544093	0,00034568	0,0075413
ENSG00000198961	PJA2	-1,5427631	0,00071962	0,01121793
ENSG00000141030	COPS3	-1,5421275	6,3186E-05	0,00347031
ENSG00000183604	SMG1P5	-1,5396437	0,00465212	0,03422925
ENSG00000065978	YBX1	-1,5392975	0,00138297	0,01621794
ENSG00000104472	CHRAC1	-1,5389364	2,4342E-05	0,00233163
ENSG00000104885	DOT1L	-1,538866	0,00289909	0,02533445
ENSG00000011007	ELOA	-1,5386703	0,0045458	0,03367602
ENSG00000071462	BUD23	-1,5371673	0,00173906	0,01850048
ENSG00000168438	CDC40	-1,5363589	0,00365334	0,02934261
ENSG00000111328	CDK2AP1	-1,5358855	0,00079334	0,01172189
ENSG00000164609	SLU7	-1,5354172	0,00027603	0,00668833
ENSG00000100532	CGRRF1	-1,5349048	0,00099505	0,01344327
ENSG00000163659	TIPARP	-1,5330827	0,00295973	0,02567224
ENSG00000136699	SMPD4	-1,5320939	0,00248197	0,02308818
ENSG00000196756	SNHG17	-1,5317573	0,00587659	0,03968597
ENSG00000182899	RPL35A	-1,5283942	6,1549E-05	0,00343784
ENSG00000064012	CASP8	-1,5276084	0,00097006	0,01322029
ENSG00000144560	VGLL4	-1,52722	0,0064373	0,04225081
ENSG00000155508	CNOT8	-1,5268935	0,00244613	0,02289341
ENSG00000103274	NUBP1	-1,5261115	0,00013863	0,0048288
ENSG00000134352	IL6ST	-1,5246728	0,00077317	0,01158855
ENSG00000223773	CD99P1	-1,5239963	6,0643E-05	0,00341061
ENSG00000148926	ADM	-1,5221721	1,3383E-05	0,00177415
ENSG00000155545	MIER3	-1,5211048	0,0023951	0,02256253
ENSG00000090520	DNAJB11	-1,5209554	0,00027454	0,00665886
ENSG00000204178	TMEM57	-1,5195792	0,00021673	0,0060254
ENSG00000165637	VDAC2	-1,5191953	0,00036382	0,00772987
ENSG00000119682	AREL1	-1,5187602	0,00286319	0,02515527
ENSG00000154582	ELOC	-1,5179657	1,0932E-05	0,00167542
ENSG00000120709	FAM53C	-1,5179444	0,00028817	0,00692078
ENSG00000006327	TNFRSF12A	-1,5166765	6,8703E-05	0,00360489
ENSG00000086598	TMED2	-1,5158135	0,00023988	0,00627635
ENSG00000138434	SSFA2	-1,5143154	0,00084169	0,01210519
ENSG00000181220	ZNF746	-1,5127894	0,00047836	0,00885939
ENSG00000182827	ACBD3	-1,51273	9,0603E-06	0,0015563
ENSG00000162923	WDR26	-1,5124333	0,00034893	0,00758211
ENSG00000122406	RPL5	-1,5122884	1,2398E-05	0,00174716
ENSG00000167522	ANKRD11	-1,511768	1,5334E-05	0,00191839
ENSG00000113580	NR3C1	-1,5105798	6,266E-06	0,00130246
ENSG00000156502	SUPV3L1	-1,5100722	0,00536043	0,03748025

ENSG00000134333	LDHA	-1,5083255	0,00082809	0,01200799
ENSG00000076321	KLHL20	-1,5069341	0,00020031	0,00577845
ENSG00000105991	HOXA1	-1,5049651	0,00480805	0,03498466
ENSG00000121864	ZNF639	-1,5044182	0,00023656	0,00623987
ENSG00000123595	RAB9A	-1,5040803	0,00119668	0,01488027
ENSG00000136986	DERL1	-1,5027124	4,3884E-05	0,00286956
ENSG00000184863	RBM33	-1,5023784	0,00016711	0,00527776
ENSG00000170638	TRABD	-1,5020257	0,00200769	0,02019972
ENSG00000271303	SRXN1	-1,5017594	6,9078E-05	0,00360489
ENSG00000113734	BNIP1	-1,5017031	0,00270093	0,02438662

Supplemental Table 6. Differentially expressed genes detected by RNA-seq in CD1c+ cDC from synovial fluid of n=3 rheumatoid arthritis (RA) versus n=3 Calcium Pyrophosphate Deposition (CPPD) Crystals-associated arthritis patients.

Transcipt ID	Gene name	Log2(FC) RA vs CPPD	Nom. P value
ENSG00000236324	AL035634.1	0,04413487	2,8166E-05
ENSG00000151376	ME3	0,2787453	7,3632E-05
ENSG00000066044	ELAVL1	-0,3972953	7,5638E-05
ENSG00000107099	DOCK8	-0,7399609	8,4494E-05
ENSG00000122490	PQLC1	-0,4720239	0,00010181
ENSG00000180881	CAPS2	0,05626666	0,00011238
ENSG00000119231	SENP5	0,59139435	0,00013926
ENSG00000184640	SEPT9	-0,6552974	0,00017242
ENSG00000273559	CWC25	0,56064912	0,00019546
ENSG00000164418	GRIK2	0,02046335	0,00022135
ENSG00000100364	KIAA0930	-0,7298747	0,00026625
ENSG00000251474	RPL32P3	0,32603879	0,00026868
ENSG00000175220	ARHGAP1	-0,4265553	0,00029408
ENSG00000176170	SPHK1	0,67327158	0,00038197
ENSG00000159658	EFCAB14	-0,5796293	0,0004103
ENSG00000200463	SNORD118	0,0437644	0,00041997
ENSG00000075407	ZNF37A	0,59784885	0,00044136
ENSG00000166265	CYYR1	-0,4311383	0,00058213
ENSG00000104365	IKBKB	-0,3166059	0,00063733
ENSG00000198690	FAN1	-0,2816823	0,0007078
ENSG00000099810	MTAP	-0,5501413	0,00080355
ENSG00000081237	PTPRC	-0,1771452	0,00086054
ENSG00000102172	SMS	0,49517841	0,00086974
ENSG00000183208	GDPGP1	0,05254401	0,00091327
ENSG00000253558	AC024568.1	0,16689599	0,00101779
ENSG00000250264	AL669918.1	-0,7551793	0,00107893
ENSG00000197961	ZNF121	0,36177087	0,00110403
ENSG00000185614	FAM212A	0,04863385	0,00134027
ENSG00000178149	DALRD3	0,23119043	0,00136963
ENSG00000127328	RAB3IP	0,36256423	0,00139724
ENSG00000113580	NR3C1	0,21336152	0,0014387
ENSG00000146826	C7orf43	-0,0854818	0,00149468
ENSG00000033100	CHPF2	-0,6136945	0,00156834
ENSG00000164970	FAM219A	0,08377457	0,00160821
ENSG00000122121	XPNPEP2	-0,1752661	0,00163048
ENSG00000276952	AL121772.3	0,03310734	0,00167489
ENSG00000264247	LINC00909	-0,1309243	0,00183905

ENSG00000272219	AC005072.1	0,1141836	0,00184377
ENSG00000249961	TERB1	0,01958259	0,00190133
ENSG00000132405	TBC1D14	-1,4380523	0,00191829
ENSG00000116120	FARSB	-0,3208522	0,00209006
ENSG00000119711	ALDH6A1	-0,348771	0,00224793
ENSG00000175826	CTDNEP1	-0,3302751	0,00226234
ENSG00000166289	PLEKHF1	-0,0564246	0,00227034
ENSG00000277476	AC005332.8	0,07624984	0,0023447
ENSG00000279331	RBM12B-AS1	0,02161593	0,00240169
ENSG00000126012	KDM5C	0,45909801	0,00243604
ENSG00000101391	CDK5RAP1	0,39628792	0,00245031
ENSG00000169246	NPIPB3	0,5430996	0,00245055
ENSG00000182158	CREB3L2	-0,6394086	0,00253278
ENSG00000233845	AC093732.1	0,06021491	0,00253328
ENSG00000204271	SPIN3	-0,2322425	0,0025789
ENSG00000259972	AC009120.2	0,22232572	0,002587
ENSG00000277865	GOLGA6L22	0,01582296	0,00260596
ENSG00000142459	EVI5L	-0,2056085	0,00267369
ENSG00000260272	AC093525.2	-0,0263569	0,00270323
ENSG00000269752	AC008761.2	0,01779376	0,00275168
ENSG00000089159	PXN	-0,6511732	0,00276604
ENSG00000177595	PIDD1	-0,176804	0,00285449
ENSG00000256206	AC018523.2	-0,3570329	0,00295253
ENSG00000178997	EXD1	0,0381082	0,0030042
ENSG00000203761	MSTO2P	0,09291765	0,00305253
ENSG00000237914	SIRPG-AS1	0,01651323	0,00306017
ENSG00000196591	HDAC2	-0,2098289	0,00332972
ENSG00000259354	AC025580.2	0,18380604	0,0033833
ENSG00000105204	DYRK1B	-0,1007025	0,00339152
ENSG00000173889	PHC3	-0,3104746	0,00341004
ENSG00000116212	LRRC42	0,33304378	0,00346433
ENSG00000137200	CMTR1	-0,6358545	0,00347037
ENSG00000278774	U2	0,87989571	0,00351114
ENSG00000138071	ACTR2	-0,5253385	0,00354013
ENSG00000258050	AL139316.1	0,01700951	0,00355059
ENSG00000149485	FADS1	0,47375331	0,00363258
ENSG00000274598	AC087893.1	0,08424228	0,00364084
ENSG00000136485	DCAF7	-0,3727641	0,00375595
ENSG00000125962	ARMCX5	-0,3709361	0,00386137
ENSG00000111676	ATN1	-0,0832679	0,00387567
ENSG00000246203	AL353807.3	0,08034114	0,00391055
ENSG00000162688	AGL	-0,5298671	0,00402618
ENSG00000128923	MINDY2	0,36462771	0,00408648

ENSG00000130175	PRKCSH	-0,5529391	0,00413349
ENSG00000229509	AC244023.1	0,05199782	0,00423073
ENSG00000166783	MARF1	-0,2799936	0,0042365
ENSG00000186111	PIP5K1C	-0,3545879	0,00430162
ENSG00000249870	AC005920.2	0,01588069	0,00430451
ENSG00000279010	AL031587.6	0,26915441	0,00432789
ENSG00000165458	INPPL1	-0,3624529	0,00449835
ENSG00000189223	PAX8-AS1	-1,0474591	0,00457727
ENSG00000251139	AC084871.1	0,02293316	0,00465015
ENSG00000078814	MYH7B	0,04613281	0,00466337
ENSG00000279608	AL353795.3	0,14532608	0,00468059
ENSG00000242156	AC000041.1	-0,0221784	0,00475257
ENSG00000183032	SLC25A21	0,01156443	0,00477011
ENSG00000183091	NEB	0,05844868	0,0049399
ENSG00000162065	TBC1D24	-0,0916067	0,00494196
ENSG00000229807	XIST	3,04456297	0,00502295
ENSG00000106780	MEGF9	-0,7830079	0,00509024
ENSG00000156787	TBC1D31	-0,2938265	0,00509804
ENSG00000100280	AP1B1	-0,9824889	0,00531633
ENSG00000113552	GNPDA1	-0,5707424	0,00531951
ENSG00000146828	SLC12A9	-0,4154437	0,00533099
ENSG00000272345	AL031775.1	0,07104334	0,00544911
ENSG00000235478	LINC01664	0,05458707	0,00548633
ENSG00000121104	FAM117A	-0,2716481	0,0056124
ENSG00000163513	TGFBR2	-0,8472339	0,00567547
ENSG00000025434	NR1H3	0,13690766	0,00569356
ENSG00000278133	AC135050.6	-0,2116855	0,00575155
ENSG00000251364	AC107884.1	-0,1178506	0,00579204
ENSG00000160799	CCDC12	0,33411184	0,00594564
ENSG00000085644	ZNF213	-0,1133384	0,00601551
ENSG00000162458	FBLIM1	0,07170574	0,00602788
ENSG00000121680	PEX16	0,29741918	0,00616302
ENSG00000130997	POLN	0,16838275	0,00631163
ENSG00000260727	SLC7A5P1	0,05015752	0,00631442
ENSG00000243678	NME2	0,29443145	0,00638759
ENSG00000261829	AC009407.1	0,01701565	0,00643642
ENSG00000265018	AGAP12P	0,17558402	0,00666721
ENSG00000113657	DPYSL3	0,40330999	0,00670026
ENSG00000231365	AL359915.2	0,09525531	0,00673687
ENSG00000167604	NFKBID	1,25943071	0,00684504
ENSG00000228486	C2orf92	0,08530151	0,00687405
ENSG00000126767	ELK1	-0,5092141	0,00690054
ENSG00000165997	ARL5B	0,64919345	0,00699502

ENSG00000235790	AC114488.2	0,01413567	0,00701242
ENSG00000188511	C22orf34	-0,1295826	0,0070164
ENSG00000268518	AC020909.2	-0,0232088	0,00712784
ENSG00000117676	RPS6KA1	-0,5788766	0,00719201
ENSG00000228028	AC069257.1	0,11970449	0,00719629
ENSG00000161647	MPP3	0,47840506	0,00724682
ENSG00000229180	AC006001.3	0,27218005	0,00727458
ENSG00000129933	MAU2	-0,5140468	0,0072765
ENSG00000123815	COQ8B	-0,2594783	0,00739009
ENSG00000179743	FLJ37453	0,14663549	0,00749084
ENSG00000270181	BIVM-ERCC5	-0,6919548	0,00756224
ENSG00000071794	HLTF	-0,1768074	0,00756522
ENSG00000226167	AP4B1-AS1	0,0502656	0,00757713
ENSG00000189149	CRYM-AS1	0,08852173	0,0075913
ENSG00000260007	AC107871.1	0,33643724	0,00760297
ENSG00000102977	ACD	0,3098474	0,00761572
ENSG00000213563	C8orf82	0,14171535	0,00767233
ENSG00000138002	IFT172	0,22008223	0,00770954
ENSG00000175104	TRAF6	0,49157534	0,00771324
ENSG00000143190	POU2F1	0,44466553	0,0077357
ENSG00000271270	TMCC1-AS1	0,0504425	0,00775072
ENSG00000215256	DHRS4-AS1	-0,4775055	0,00778153
ENSG00000272644	AC097468.3	0,03568667	0,00790176
ENSG00000130725	UBE2M	-0,2440445	0,00790548
ENSG00000256218	AC007848.2	0,0636682	0,00790602
ENSG00000259563	AC025430.1	0,03446511	0,0079081
ENSG00000261684	AC018362.1	-0,3136431	0,00796767
ENSG00000224599	BMS1P12	0,0342179	0,00807453
ENSG00000107815	TWNK	-0,1375747	0,0080853
ENSG00000269145	AC007192.2	0,03946072	0,00812107
ENSG00000270084	GAS5-AS1	0,02262035	0,00812315
ENSG00000123243	ITIH5	0,02628742	0,00818395
ENSG00000198553	KCNRG	0,06974554	0,00827704
ENSG00000108774	RAB5C	-0,6978494	0,00837205
ENSG00000279416	AC099689.1	0,03190756	0,00837814
ENSG00000131188	PRR7	0,04046716	0,00842042
ENSG00000100276	RASL10A	0,42420733	0,00849259
ENSG00000269755	AC008758.6	-0,0854232	0,00855445
ENSG00000275888	AC132872.3	0,02393064	0,00859491
ENSG00000176055	MBLAC2	-0,1974193	0,00860591
ENSG00000010610	CD4	-0,4837938	0,00870584
ENSG00000243660	ZNF487	0,34814381	0,00872901
ENSG00000261140	AC093525.4	0,01285498	0,00890002

ENSG00000146409	SLC18B1	-0,5802829	0,00892441
ENSG00000170858	LILRP2	-0,0195646	0,00903825
ENSG00000250069	AC011379.1	0,10718964	0,0090425
ENSG00000267221	C17orf113	0,05315482	0,00904335
ENSG00000222724	RNU2-63P	0,10363916	0,00904713
ENSG00000248458	AL139147.1	0,26487985	0,00906181
ENSG00000204519	ZNF551	-0,158469	0,00920206
ENSG00000173457	PPP1R14B	0,30004081	0,00921846
ENSG00000064313	TAF2	0,41444197	0,00931721
ENSG00000177201	OR2T12	0,06525848	0,00933619
ENSG00000167074	TEF	-0,1267592	0,00941463
ENSG00000160691	SHC1	-0,5479219	0,00949571
ENSG00000142230	SAE1	0,48139623	0,00960204
ENSG00000168286	THAP11	-0,2783236	0,00966405
ENSG00000133884	DPF2	-0,6554378	0,00977563
ENSG00000274845	uc_338	0,03352688	0,00980286
ENSG00000112062	MAPK14	-0,6588506	0,00980782
ENSG00000258659	TRIM34	-0,4996997	0,00981188
ENSG00000242588	AC108010.1	0,04913014	0,00981258
ENSG00000260852	FBXL19-AS1	0,1073475	0,00981813
ENSG00000189339	SLC35E2B	-0,7043823	0,00988519
ENSG00000088247	KHSRP	-0,2729439	0,01000468
ENSG00000213654	GPSM3	-0,5055367	0,0100226
ENSG00000259330	INAFM2	-0,7843612	0,01005347
ENSG00000279861	AC073548.1	0,06461739	0,01005712
ENSG00000281207	SLFNL1-AS1	0,0323724	0,01006371
ENSG00000135124	P2RX4	0,64864714	0,01007422
ENSG00000174946	GPR171	-0,4719514	0,01014554
ENSG00000164823	OSGIN2	0,49618517	0,01018275
ENSG00000023191	RNH1	0,36322611	0,01032933
ENSG00000254431	AC084083.1	0,04190279	0,01049956
ENSG00000111885	MAN1A1	-0,8086805	0,01052267
ENSG00000139629	GALNT6	-0,3659243	0,01063481
ENSG00000015285	WAS	-0,3986954	0,01074738
ENSG00000101752	MIB1	-0,3981572	0,01081097
ENSG00000280399	AC022497.1	0,04848344	0,01087231
ENSG00000264229	RNU4ATAC	0,09854947	0,01087814
ENSG00000180964	TCEAL8	-0,3346415	0,01093501
ENSG00000277668	Metazoa_SRP	0,03486177	0,01093851
ENSG00000258704	SRP54-AS1	0,08506094	0,01096983
ENSG00000108344	PSMD3	-0,4608135	0,01099785
ENSG00000169957	ZNF768	-0,1260377	0,01100202
ENSG00000133488	SEC14L4	0,02555657	0,01103165

ENSG00000100209	HSCB	-0,213152	0,01117356
ENSG00000161847	RAVER1	-0,2471424	0,01117529
ENSG00000201519	RNU6-645P	0,00887001	0,01118434
ENSG00000165105	RASEF	0,30900577	0,01123574
ENSG00000106258	CYP3A5	0,15166477	0,01127452
ENSG00000249252	AC098829.1	0,00789269	0,01127826
ENSG00000176654	NANOGP1	0,00789269	0,01127826
ENSG00000267543	AC015802.3	0,03322924	0,01131095
ENSG00000259956	RBM15B	-0,5763117	0,01132289
ENSG00000228224	NACAP1	0,03501617	0,01133191
ENSG00000137692	DCUN1D5	-0,2976847	0,01134496
ENSG00000198218	QRICH1	0,40990648	0,01141615
ENSG00000151693	ASAP2	0,27977356	0,01150704
ENSG00000160714	UBE2Q1	-0,205258	0,01158908
ENSG00000278920	AC005005.4	0,07835144	0,01161394
ENSG00000174945	AMZ1	0,07105458	0,01164565
ENSG00000161011	SQSTM1	0,76164659	0,01168524
ENSG00000261804	AC007342.4	0,05225235	0,01169392
ENSG00000132915	PDE6A	0,07739287	0,01171055
ENSG00000270140	AC005520.3	0,03314142	0,01175427
ENSG00000259773	AC012100.2	0,02170853	0,01177148
ENSG00000254093	PINX1	0,19546866	0,01180922
ENSG00000115271	GCA	-0,9517501	0,01181633
ENSG00000214182	PTMAP5	0,23992356	0,01182873
ENSG00000186862	PDZD7	0,12201	0,01183774
ENSG00000231826	LINC01819	-0,0831203	0,01184662
ENSG00000142409	ZNF787	-0,0812477	0,01187961
ENSG00000080824	HSP90AA1	0,74828699	0,01192024
ENSG00000154511	FAM69A	0,45801646	0,01198463
ENSG00000162231	NXF1	0,62802556	0,01208974
ENSG00000219545	UMAD1	-0,4477987	0,01212052
ENSG00000102225	CDK16	0,4821612	0,01214459
ENSG00000164332	UBLCP1	-0,5418413	0,01217668
ENSG00000203588	IGBP1-AS1	0,02565574	0,01218378
ENSG00000279412	AC020763.3	0,04582311	0,01221205
ENSG00000260235	AC105020.3	0,05004099	0,01221286
ENSG00000260269	AC105036.3	0,11749097	0,01229785
ENSG00000204381	LAYN	-0,2184094	0,0123687
ENSG00000212607	SNORA3B	0,00977041	0,01244223
ENSG00000122778	KIAA1549	0,01574301	0,01245399
ENSG00000100345	MYH9	-0,837988	0,0125348
ENSG00000103479	RBL2	-0,534661	0,01261254
ENSG00000196453	ZNF777	-0,2314911	0,01270973

ENSG00000135473	PAN2	-0,4611363	0,01271888
ENSG00000178585	CTNNBIP1	-0,3216808	0,01272375
ENSG00000206417	H1FX-AS1	0,06218783	0,01275596
ENSG00000215717	TMEM167B	-0,3355952	0,01280176
ENSG00000258908	AL355075.3	0,13240923	0,01287428
ENSG00000168918	INPP5D	-0,733761	0,01288781
ENSG00000256646	AC010132.3	0,64406981	0,0129039
ENSG00000174667	OR7D4	0,03543421	0,01293276
ENSG00000149499	EML3	-0,3671501	0,01312667
ENSG00000005075	POLR2J	-0,1544824	0,01317559
ENSG00000136045	PWP1	0,30458438	0,01328654
ENSG00000128918	ALDH1A2	-0,129158	0,0134752
ENSG00000260252	AC009087.1	-0,0207719	0,01357601
ENSG00000273156	AC124016.2	0,02341199	0,01363152
ENSG00000279891	FLJ42393	0,22389985	0,01368376
ENSG00000169375	SIN3A	0,6840291	0,01368522
ENSG00000257218	GATC	-0,4310401	0,01380242
ENSG00000104964	AES	0,20757602	0,01382066
ENSG00000090060	PAPOLA	-0,3369715	0,0138838
ENSG00000188266	HYKK	0,01792399	0,01397308
ENSG00000150455	TIRAP	-0,2951498	0,01407616
ENSG00000033867	SLC4A7	-0,8108015	0,01419603
ENSG00000273319	AC058791.1	0,8154672	0,01421132
ENSG00000214367	HAUS3	0,24422215	0,01422247
ENSG00000196208	GREB1	0,02482208	0,01427246
ENSG00000106723	SPIN1	-0,5227884	0,01443593
ENSG00000213722	DDAH2	0,61578837	0,01450967
ENSG00000254737	OR10G4	0,04093401	0,01456138
ENSG00000136560	TANK	0,69187215	0,01458468
ENSG00000082146	STRADB	-0,5044698	0,01459703
ENSG00000215014	AL645728.1	0,06559054	0,01478893
ENSG00000169251	NMD3	-0,3811337	0,01479779
ENSG00000074370	ATP2A3	-0,4571078	0,01480424
ENSG00000103275	UBE2I	-0,3797751	0,01488956
ENSG00000248476	BACH1-IT1	0,08165461	0,01489481
ENSG00000177189	RPS6KA3	-0,6262512	0,01501708
ENSG00000124702	KLHDC3	-0,6454344	0,01503712
ENSG00000284052	AC006460.2	0,04230466	0,01507133
ENSG00000132874	SLC14A2	0,01662438	0,01512186
ENSG00000067057	PFKP	0,83865565	0,01517084
ENSG00000114395	CYB561D2	0,20828785	0,01517874
ENSG00000270641	TSIX	0,61690451	0,0151881
ENSG00000102032	RENBP	0,05879758	0,01525178

ENSG00000228918	LINC01344	0,00595856	0,01525597
ENSG00000187847	OR7E25P	0,01777097	0,01525597
ENSG00000133101	CCNA1	0,41347082	0,01529474
ENSG00000272501	AL662844.4	0,14425614	0,01545678
ENSG00000274512	TBC1D3L	0,3183011	0,01547683
ENSG00000165533	TTC8	-0,2952769	0,0155092
ENSG00000106546	AHR	-0,631426	0,01551704
ENSG00000085185	BCORL1	0,04058892	0,01552943
ENSG00000171206	TRIM8	-0,3969157	0,01567769
ENSG00000243364	EFNA4	0,08398454	0,01568418
ENSG00000203364	AL390760.1	0,03671881	0,01571228
ENSG00000171291	ZNF439	0,42021564	0,01572947
ENSG00000213780	GTF2H4	-0,2589755	0,01578338
ENSG00000140090	SLC24A4	-0,2563525	0,01578838
ENSG00000263494	AC004702.1	0,04585166	0,01579255
ENSG00000261279	ULK4P1	0,87778783	0,01583367
ENSG00000176915	ANKLE2	0,57126186	0,0158684
ENSG00000085224	ATRX	0,34909593	0,01591809
ENSG00000176463	SLCO3A1	0,63687948	0,01602387
ENSG00000204130	RUFY2	0,22133273	0,01604195
ENSG00000138078	PREPL	-0,4469861	0,01605284
ENSG00000254254	AC012349.1	0,05996325	0,01615555
ENSG00000144550	CPNE9	-0,056278	0,01617782
ENSG00000196235	SUPT5H	-0,2871477	0,01619886
ENSG00000275832	ARHGAP23	0,07107087	0,01626043
ENSG00000206828	U1	0,33396381	0,01629697
ENSG00000181036	FCRL6	-0,2840966	0,0163218
ENSG00000257621	PSMA3-AS1	0,25489584	0,01638283
ENSG00000069424	KCNAB2	-0,2655806	0,01646662
ENSG00000260219	AC106782.2	0,04545812	0,0164859
ENSG00000245293	AC096564.1	0,05817298	0,016535
ENSG00000139266	MARCH9	0,3558817	0,01655103
ENSG00000070182	SPTB	0,04629203	0,01655943
ENSG00000187650	VMAC	-0,0756011	0,01659004
ENSG00000115841	RMDN2	-0,1645324	0,01662726
ENSG00000111731	C2CD5	-0,3700651	0,01667362
ENSG00000278999	AC008985.1	0,04679646	0,01673355
ENSG00000133424	LARGE1	0,02300469	0,01681245
ENSG00000163807	KIAA1143	-0,4010728	0,01686135
ENSG00000250041	AC069360.1	0,05703169	0,01686461
ENSG00000101199	ARFGAP1	-0,1933427	0,016932
ENSG00000074356	NCBP3	0,45031098	0,01714076
ENSG00000258655	ARHGAP5-AS1	-0,0986119	0,01717867

ENSG00000177853	ZNF518A	0,48675788	0,01718841
ENSG00000180917	CMTR2	-0,4965662	0,01721629
ENSG00000154359	LONRF1	0,66703138	0,01739361
ENSG00000259342	AC025580.1	0,09578151	0,01744739
ENSG00000175489	LRRC25	-0,4888447	0,01768095
ENSG00000167962	ZNF598	-0,1377189	0,01769989
ENSG00000262420	AC007613.1	0,03826325	0,01783815
ENSG00000100906	NFKBIA	1,04228457	0,01801406
ENSG00000172922	RNASEH2C	0,27098402	0,01803096
ENSG00000173692	PSMD1	0,3725807	0,01812291
ENSG00000277688	AC243585.2	0,0251436	0,01813232
ENSG00000196636	SDHAF3	-0,2604031	0,01814523
ENSG00000091542	ALKBH5	-0,4112265	0,01819423
ENSG00000145911	N4BP3	0,27278116	0,01838137
ENSG00000136100	VPS36	-0,5378078	0,01839466
ENSG00000259782	AC008915.1	0,03734747	0,01842839
ENSG00000236709	DAPK1-IT1	0,1007792	0,01843843
ENSG00000197140	ADAM32	0,08485257	0,01844831
ENSG00000284624	AC092902.5	0,36738097	0,01847085
ENSG00000186767	SPIN4	-0,0900211	0,01848318
ENSG00000147471	PLPBP	-0,4804459	0,01848467
ENSG00000126070	AGO3	0,34902896	0,01851086
ENSG00000177669	MBOAT4	-0,0557773	0,01851947
ENSG00000255421	AP002340.1	0,01839875	0,01853829
ENSG00000260661	AC116903.2	0,03251757	0,01855926
ENSG00000201076	RNU4-51P	0,01712664	0,01863358
ENSG00000123739	PLA2G12A	-0,2669248	0,01876667
ENSG00000152223	EPG5	0,48737208	0,01882417
ENSG00000157593	SLC35B2	0,3033321	0,01886353
ENSG00000048649	RSF1	0,28789588	0,01887202
ENSG00000167553	TUBA1C	0,94931168	0,01889731
ENSG00000234572	LINC01800	0,12267742	0,01889831
ENSG00000168237	GLYCTK	0,26582178	0,01890679
ENSG00000127452	FBXL12	-0,1955299	0,01894624
ENSG00000279080	AL022322.2	0,23444708	0,01898193
ENSG00000250893	AC098869.2	0,07173409	0,01900254
ENSG00000116473	RAP1A	-0,6387361	0,01904291
ENSG00000244203	FOXP1-AS1	0,05459795	0,01906241
ENSG00000238164	TNFRSF14-AS1	0,10717282	0,01911934
ENSG00000117691	NENF	0,37812183	0,01914976
ENSG00000163125	RPRD2	-0,4371458	0,01918964
ENSG00000148187	MRRF	-0,2796412	0,01920035
ENSG00000188554	NBR1	-0,6174483	0,01924174

ENSG00000131236	CAP1	-0,3393758	0,0192781
ENSG00000197062	ZSCAN26	-0,1564293	0,01928943
ENSG00000241973	PI4KA	-0,5459563	0,01930113
ENSG00000153107	ANAPC1	-0,7189926	0,0193164
ENSG00000173207	CKS1B	0,49531171	0,01931668
ENSG00000163754	GYG1	-0,3599119	0,01933442
ENSG00000142197	DOPEY2	-0,2173792	0,01936795
ENSG00000148396	SEC16A	-0,4658525	0,01954956
ENSG00000172315	TP53RK	-0,3585463	0,01955041
ENSG00000134086	VHL	-0,4941051	0,01956397
ENSG00000204351	SKIV2L	-0,3726273	0,01965123
ENSG00000244405	ETV5	-0,6213944	0,0197739
ENSG00000233061	TTLL7-IT1	0,00757474	0,01979637
ENSG00000048471	SNX29	0,50929207	0,01980979
ENSG00000123096	SSPN	0,22302614	0,01986069
ENSG00000143183	TMCO1	-0,2121015	0,01999129
ENSG00000136699	SMPD4	-0,3950209	0,02002108
ENSG00000154640	BTG3	0,57607854	0,02017698
ENSG00000105376	ICAM5	0,11077506	0,02022386
ENSG00000177951	BET1L	-0,3071913	0,02028525
ENSG00000280234	AC124303.2	0,03807342	0,02030216
ENSG00000081189	MEF2C	-0,9682965	0,02030801
ENSG00000188906	LRRK2	-0,4113862	0,02034193
ENSG00000261444	AC106782.4	-0,0146862	0,02041818
ENSG00000108021	FAM208B	0,50347169	0,02043714
ENSG00000261654	AL360270.2	0,16074834	0,02048712
ENSG00000113272	THG1L	-0,3050236	0,0205951
ENSG00000087365	SF3B2	-0,4087576	0,02059741
ENSG00000103426	CORO7-PAM16	0,07585452	0,02067359
ENSG00000198740	ZNF652	-0,5960832	0,02068916
ENSG00000257341	AL928654.4	1,46396224	0,02073179
ENSG00000249267	LINC00939	0,0150342	0,02106627
ENSG00000108828	VAT1	0,63468566	0,02115871
ENSG00000162302	RPS6KA4	-0,5127723	0,02116004
ENSG00000083896	YTHDC1	0,31918074	0,02118264
ENSG00000079691	CARMIL1	-0,2471877	0,02119887
ENSG00000078401	EDN1	0,14546046	0,02127441
ENSG00000148730	EIF4EBP2	-0,4737142	0,02131828
ENSG00000173805	HAP1	0,02122932	0,02133199
ENSG00000170145	SIK2	-0,1596438	0,02140812
ENSG00000225860	LINC02072	0,01829807	0,02142623
ENSG00000198951	NAGA	-0,3281593	0,0214824
ENSG00000259954	IL21R-AS1	0,03716543	0,0215278

ENSG00000175215	CTDSP2	-0,8567166	0,02160329
ENSG00000178115	GOLGA8Q	0,05454814	0,02163371
ENSG00000197956	S100A6	0,60472334	0,02177335
ENSG00000010626	LRRC23	0,10249652	0,02185782
ENSG00000101417	PXMP4	-0,1412632	0,02193149
ENSG00000279345	Z98885.3	0,02628465	0,02195402
ENSG00000096384	HSP90AB1	0,50967702	0,02200343
ENSG00000014164	ZC3H3	-0,3658888	0,02214372
ENSG00000085511	MAP3K4	-0,375754	0,02216094
ENSG00000275385	CCL18	0,24636897	0,02217315
ENSG00000117419	ERI3	-0,2347393	0,02217802
ENSG00000104894	CD37	-0,3249639	0,02225418
ENSG00000217801	AL390719.1	0,64629391	0,02226273
ENSG00000270800	RPS10-NUDT3	-0,4947971	0,02232852
ENSG00000275807	AC145285.7	0,00882263	0,02238378
ENSG00000273723	AL139089.1	0,04797786	0,02240104
ENSG00000267060	PTGES3L	0,05699705	0,02241222
ENSG00000129197	RPAIN	0,25743486	0,02250714
ENSG00000172831	CES2	-0,4489525	0,02265151
ENSG00000280543	ASAP1-IT2	0,10167724	0,02273289
ENSG00000087152	ATXN7L3	-0,342822	0,02275249
ENSG00000223797	ENTPD3-AS1	0,11161327	0,02277158
ENSG00000117597	DIEXF	-0,5682967	0,0227789
ENSG00000230068	CDC42-IT1	0,0170188	0,02291401
ENSG00000185010	F8	-0,1330761	0,02293833
ENSG00000063761	ADCK1	-0,249083	0,02300384
ENSG00000188352	FOCAD	-0,3642685	0,02302512
ENSG00000053372	MRTO4	-0,1760793	0,02307596
ENSG00000188171	ZNF626	-0,2479216	0,02307864
ENSG00000100764	PSMC1	0,4473019	0,0231034
ENSG00000204764	RANBP17	0,05199256	0,02313767
ENSG00000165813	CCDC186	0,3465419	0,02314351
ENSG00000181392	SYNE4	-0,0235309	0,02316097
ENSG00000198909	MAP3K3	-0,8251761	0,02324038
ENSG00000090104	RGS1	1,47527493	0,02325279
ENSG00000196646	ZNF136	0,47759376	0,02336429
ENSG00000132970	WASF3	-0,053126	0,02336879
ENSG00000163485	ADORA1	0,108796	0,02339276
ENSG00000259932	AC051619.7	-0,0241888	0,02355756
ENSG00000129667	RHBDF2	0,3832444	0,02359258
ENSG00000225764	P3H2-AS1	0,01893775	0,02360108
ENSG00000135404	CD63	0,64446253	0,02368181
ENSG00000183154	AC138356.1	-0,0786875	0,02385055

ENSG00000157106	SMG1	0,94666582	0,02387192
ENSG00000119725	ZNF410	0,34731849	0,02388198
ENSG00000156113	KCNMA1	0,10762664	0,02395395
ENSG00000105398	SULT2A1	0,01026055	0,0239623
ENSG00000143127	ITGA10	-0,05885	0,02396406
ENSG00000272196	HIST2H2AA4	0,67623617	0,02398389
ENSG00000165886	UBTD1	0,14634139	0,02405218
ENSG00000105298	CACTIN	-0,218648	0,02407628
ENSG00000162076	FLYWCH2	0,07631974	0,02408475
ENSG00000232859	LYRM9	-0,1960328	0,02421163
ENSG00000264456	AC138207.4	0,08312141	0,02424756
ENSG00000136527	TRA2B	0,34850899	0,02433769
ENSG00000116824	CD2	-0,4599442	0,02439842
ENSG00000197747	S100A10	0,72568485	0,02447111
ENSG00000111752	PHC1	-0,5268651	0,02457613
ENSG00000187514	PTMA	0,50525408	0,02457646
ENSG00000229715	EEF1DP3	0,31355967	0,02462255
ENSG00000113327	GABRG2	0,11347993	0,02472193
ENSG00000185522	LMNTD2	0,08344916	0,02476069
ENSG00000273628	AL354798.1	-0,1194064	0,02492821
ENSG00000233178	AL161457.2	-0,314747	0,0249855
ENSG00000259607	AC108449.3	0,06850678	0,0250586
ENSG00000254263	AC022973.3	0,04655118	0,02509004
ENSG00000147050	KDM6A	0,63278342	0,02513426
ENSG00000139572	GPR84	0,76403043	0,02513682
ENSG00000135972	MRPS9	-0,280251	0,02516028
ENSG00000219607	PPP1R3G	-0,1758389	0,02518923
ENSG00000101558	VAPA	0,21592548	0,02520645
ENSG00000071626	DAZAP1	-0,2487069	0,025345
ENSG00000259494	MRPL46	-0,0967041	0,02534922
ENSG00000279801	AC111170.3	0,0489005	0,02536743
ENSG00000213793	ZNF888	-0,1369157	0,02542075
ENSG00000133704	IPO8	-0,3044628	0,02548795
ENSG00000269028	MTRNR2L12	0,04556052	0,0254954
ENSG00000136870	ZNF189	0,12130035	0,02551831
ENSG00000188037	CLCN1	-0,0956777	0,02562315
ENSG00000106615	RHEB	0,62413091	0,0256353
ENSG00000160211	G6PD	-0,0966951	0,02575279
ENSG00000101138	CSTF1	-0,7615511	0,02577348
ENSG00000007392	LUC7L	0,41170274	0,02590144
ENSG00000274963	RN7SL600P	0,08626627	0,02595269
ENSG00000105227	PRX	-0,0465501	0,0259674
ENSG00000144283	PKP4	0,27784236	0,02605589

ENSG00000188725	SMIM15	-0,2814367	0,02608867
ENSG00000234678	ELF3-AS1	0,02532288	0,02610245
ENSG00000001460	STPG1	0,2593881	0,0261464
ENSG00000112149	CD83	1,5004025	0,02614686
ENSG00000271601	LIX1L	-0,530712	0,02624629
ENSG00000278107	AC027575.4	0,0186535	0,02630233
ENSG00000137274	BPHL	0,21598252	0,02631795
ENSG00000154727	GABPA	-0,4720605	0,02633187
ENSG00000162769	FLVCR1	0,23998648	0,02639401
ENSG00000272693	AC073107.1	-0,0169183	0,02643079
ENSG00000213145	CRIP1	1,70087501	0,02643198
ENSG00000197182	MIRLET7BHG	0,03908803	0,02643699
ENSG00000162929	KIAA1841	0,35084472	0,0264435
ENSG00000182511	FES	-0,4432107	0,0264953
ENSG00000234147	AL035446.1	-0,1339022	0,02653372
ENSG00000103549	RNF40	-0,5349158	0,02654895
ENSG00000156219	ART3	0,01525335	0,02658315
ENSG00000163104	SMARCAD1	-0,7590264	0,02662491
ENSG00000259519	AC051619.4	0,01079983	0,02665462
ENSG00000165879	FRAT1	-0,6121801	0,0266807
ENSG00000267254	ZNF790-AS1	0,1341214	0,0266823
ENSG00000243716	NPIPB5	0,74629744	0,02669421
ENSG00000255443	CD44-AS1	0,13869959	0,02669714
ENSG00000101882	NKAP	0,40021266	0,02674481
ENSG00000151065	DCP1B	-0,4069001	0,02681509
ENSG00000215375	MYL5	-0,0836465	0,02688196
ENSG00000282961	PRNCR1	0,03088212	0,02690469
ENSG00000203865	ATP1A1-AS1	-0,0739231	0,02691269
ENSG00000254910	AC136475.2	-0,0187292	0,02694433
ENSG00000204172	AGAP9	0,15473821	0,02695655
ENSG00000106346	USP42	-0,1931149	0,02699611
ENSG00000149136	SSRP1	-0,4301055	0,0270548
ENSG00000204252	HLA-DOA	-0,7125969	0,02712538
ENSG00000260807	AC009041.2	0,03466737	0,02713178
ENSG00000132635	PCED1A	-0,1695537	0,0271927
ENSG00000168300	PCMTD1	-0,2827777	0,02723191
ENSG00000274396	HOTAIRM1_4	-0,0203712	0,02724088
ENSG00000150459	SAP18	0,28646097	0,02724423
ENSG00000005436	GCFC2	-0,369393	0,02724591
ENSG00000173110	HSPA6	1,01041229	0,02728129
ENSG00000070770	CSNK2A2	-0,1895171	0,02729869
ENSG00000232021	LEF1-AS1	0,00725556	0,02739369
ENSG00000163006	CCDC138	0,08363701	0,02744695

ENSG00000013810	TACC3	-0,5643717	0,02750499
ENSG00000113119	TMCO6	0,32366686	0,02757856
ENSG00000172671	ZFAND4	-0,253637	0,02759644
ENSG00000161551	ZNF577	0,42512711	0,02765577
ENSG00000140983	RHOT2	0,27147973	0,02765986
ENSG00000264235	AP005329.1	0,02686163	0,02767925
ENSG00000162735	PEX19	-0,3608739	0,02773635
ENSG00000180354	MTURN	-0,1624799	0,02775185
ENSG00000143198	MGST3	0,67108087	0,02785023
ENSG00000061273	HDAC7	-0,3247813	0,02799788
ENSG00000258810	AL133371.2	-0,0479273	0,02805674
ENSG00000273033	LINC02035	0,17393524	0,02809983
ENSG00000075151	EIF4G3	-0,5943991	0,02815973
ENSG00000231607	DLEU2	0,14270046	0,02818049
ENSG00000066336	SPI1	-0,5344231	0,02819702
ENSG00000182185	RAD51B	0,35004096	0,02822561
ENSG00000123143	PKN1	-0,3124683	0,02824916
ENSG00000120992	LYPLA1	-0,3217281	0,02838926
ENSG00000177283	FZD8	-0,0107268	0,02849922
ENSG00000149948	HMGA2	0,0313958	0,02860597
ENSG00000100321	SYNGR1	-0,2164201	0,02866859
ENSG00000227782	AC002553.1	0,04381918	0,02869339
ENSG00000158122	AAED1	0,52514359	0,02869562
ENSG00000271204	AC016831.5	0,65565214	0,02872743
ENSG00000125637	PSD4	-0,6193351	0,02873314
ENSG00000272463	AL357054.4	0,0622696	0,02879917
ENSG00000164329	PAPD4	-0,3774531	0,02886026
ENSG00000227825	SLC9A7P1	-0,4182075	0,02919701
ENSG00000215012	RTL10	-0,2241664	0,02926176
ENSG00000187147	RNF220	-0,5077688	0,02927061
ENSG00000131408	NR1H2	0,22822619	0,02933231
ENSG00000204695	OR14J1	0,09481218	0,02935224
ENSG00000145022	TCTA	-0,4868562	0,0294051
ENSG00000139531	SUOX	-0,4003855	0,02941944
ENSG00000198420	TCAF1	-0,4114671	0,02949498
ENSG00000143379	SETDB1	-0,5148531	0,02954525
ENSG00000225746	MEG8	0,05172958	0,02957079
ENSG00000234432	AC092171.3	0,02055602	0,02959391
ENSG00000170791	CHCHD7	-0,5296072	0,02959826
ENSG00000173171	MTX1	0,34121757	0,02974871
ENSG00000101152	DNAJC5	0,23859375	0,02978286
ENSG00000186166	CCDC84	0,31799874	0,02979962
ENSG00000119383	PTPA	-0,1754153	0,02988074

ENSG00000091490	SEL1L3	-0,3564537	0,0299104
ENSG00000117751	PPP1R8	-0,5344106	0,02996129
ENSG00000260239	LINC02533	0,00629405	0,03012406
ENSG00000185065	AC000068.1	0,0851229	0,03022424
ENSG00000182218	HHIPL1	-0,0305181	0,03023068
ENSG00000272617	COG8	-0,3448198	0,03029692
ENSG00000251562	MALAT1	1,41043426	0,03035711
ENSG00000263647	BPTFP1	0,04604304	0,03036217
ENSG00000159840	ZYX	-0,6185385	0,03040368
ENSG00000050748	MAPK9	-0,3010613	0,03043917
ENSG00000101421	CHMP4B	0,28250166	0,03050749
ENSG00000226380	AC016831.1	0,30832911	0,03056859
ENSG00000236700	LINC01010	0,13521593	0,03063039
ENSG00000225783	MIAT	0,0787777	0,03074329
ENSG00000100722	ZC3H14	-0,5003083	0,03079954
ENSG00000131759	RARA	-0,3750423	0,03090125
ENSG00000055732	MCOLN3	0,46977779	0,03097563
ENSG00000113504	SLC12A7	-0,4045137	0,03105721
ENSG00000165392	WRN	0,27883422	0,03108368
ENSG00000088038	CNOT3	-0,2487255	0,03112692
ENSG00000231304	SGO1-AS1	0,04855721	0,03117495
ENSG00000119638	NEK9	-0,5336079	0,0311828
ENSG00000262188	LINC01978	0,01090782	0,03131105
ENSG00000265566	RN7SL605P	0,05351795	0,03132372
ENSG00000115307	AUP1	0,3361291	0,03133546
ENSG00000068831	RASGRP2	0,14618705	0,03142323
ENSG00000138593	SECISBP2L	0,26390696	0,03151142
ENSG00000179532	DNHD1	0,22711231	0,03152395
ENSG00000096093	EFHC1	0,40553549	0,03153852
ENSG00000188681	TEKT4P2	0,03678797	0,03157701
ENSG00000174004	NRROS	0,26650327	0,0316757
ENSG00000163820	FYCO1	-0,2974036	0,03167998
ENSG00000267216	AC020915.2	-0,2283227	0,03177252
ENSG00000137171	KLC4	-0,358397	0,03185625
ENSG00000172000	ZNF556	0,06388413	0,03190248
ENSG00000119686	FLVCR2	-0,608535	0,03192092
ENSG00000263171	AC026954.3	0,03030213	0,03202837
ENSG00000239218	RPS20P22	0,00564061	0,03210689
ENSG00000141756	FKBP10	0,00564061	0,03210689
ENSG00000260569	AC090398.1	0,00564061	0,03210689
ENSG00000009765	IYD	0,01124683	0,03210689
ENSG00000231731	AC010976.1	0,01681903	0,03210689
ENSG00000133065	SLC41A1	0,11962768	0,03211033

ENSG00000010803	SCMH1	-0,2315506	0,03221613
ENSG00000143493	INTS7	-0,1214162	0,03225086
ENSG00000268858	AL118506.1	-0,0872106	0,03225459
ENSG00000143815	LBR	-0,8518248	0,03227028
ENSG00000251192	ZNF674	0,02043096	0,03236239
ENSG00000136944	LMX1B	0,01360342	0,03242778
ENSG00000112078	KCTD20	-0,612089	0,03247011
ENSG00000163666	HESX1	0,13550321	0,03247081
ENSG00000167842	MIS12	-0,3002642	0,03253677
ENSG00000198825	INPP5F	0,74945251	0,0326998
ENSG00000169752	NRG4	0,07543311	0,03282472
ENSG00000125817	CENPB	-0,0978514	0,0328319
ENSG00000175470	PPP2R2D	0,19048166	0,03283564
ENSG00000272822	AC073610.3	0,13357037	0,03296416
ENSG00000162885	B3GALNT2	0,66507498	0,03296512
ENSG00000226627	SHANK2-AS1	0,01980577	0,03297319
ENSG00000014123	UFL1	-0,540647	0,03297441
ENSG00000256982	AC135782.1	0,02190714	0,03303228
ENSG00000039319	ZFYVE16	0,4061361	0,03308415
ENSG00000280287	AC131212.3	0,07822841	0,03325747
ENSG00000188778	ADRB3	-0,1849684	0,03329344
ENSG00000262831	AC145207.3	0,24906226	0,03330231
ENSG00000257315	ZBED6	-0,4421458	0,03336046
ENSG00000136828	RALGPS1	0,17532027	0,03338902
ENSG00000115594	IL1R1	-0,7628859	0,03347484
ENSG00000166012	TAF1D	0,39302306	0,03350505
ENSG00000139719	VPS33A	-0,2854816	0,03350559
ENSG00000130723	PRRC2B	-0,2134057	0,03353927
ENSG00000262823	AC127521.1	0,01530722	0,03362964
ENSG00000269779	AC010329.2	0,01423183	0,03367837
ENSG00000105697	HAMP	-0,0206308	0,03377468
ENSG00000258730	ITPK1-AS1	0,33100457	0,03378117
ENSG00000145908	ZNF300	0,17583488	0,03379497
ENSG00000204389	HSPA1A	1,46843723	0,03389265
ENSG00000259556	AC090971.3	0,03143242	0,0340112
ENSG00000131653	TRAF7	-0,3950528	0,03409055
ENSG00000243753	HLA-L	0,47949822	0,03409254
ENSG00000104889	RNASEH2A	0,29284385	0,03417883
ENSG00000254873	AP001267.1	-0,2109073	0,03424673
ENSG00000116815	CD58	0,62971166	0,03425146
ENSG00000164574	GALNT10	-0,497144	0,03431259
ENSG00000133997	MED6	0,54111358	0,03433074
ENSG00000165178	NCF1C	0,57502801	0,03440063

ENSG00000258232	AC125611.3	0,70448913	0,03443687
ENSG00000128641	MYO1B	-0,3559279	0,03448759
ENSG00000280798	LINC00294	0,21881984	0,03448966
ENSG00000204920	ZNF155	0,204556	0,03462514
ENSG00000141298	SSH2	-0,5065053	0,03462827
ENSG00000113621	TXNDC15	0,17810042	0,03475687
ENSG00000139618	BRCA2	0,54791511	0,03482219
ENSG00000105778	AVL9	0,15594245	0,03482515
ENSG00000142186	SCYL1	-0,2230233	0,03483673
ENSG00000141002	TCF25	0,30628117	0,03484082
ENSG00000273356	LINC02019	0,19804493	0,03493
ENSG00000119689	DLST	-0,1855197	0,03500328
ENSG00000236053	LINC01067	-0,0123129	0,03500645
ENSG00000247315	ZCCHC3	-0,250211	0,03503332
ENSG00000080572	PIH1D3	0,01521102	0,03505334
ENSG00000089916	GPATCH2L	0,73660349	0,03505649
ENSG00000274292	AC084018.2	0,06644729	0,03506025
ENSG00000150787	PTS	0,636671	0,03510154
ENSG00000104419	NDRG1	0,2769207	0,03511102
ENSG00000093217	XYLB	0,23843897	0,03516104
ENSG00000135506	OS9	-0,3391393	0,03517958
ENSG00000172725	CORO1B	-0,2539986	0,03519985
ENSG00000184792	OSBP2	0,28236977	0,03520836
ENSG00000158636	EMSY	0,45965259	0,03525906
ENSG00000125991	ERGIC3	0,3229905	0,03526315
ENSG00000280242	AL450226.2	0,03296627	0,03526977
ENSG00000174460	ZCCHC12	-0,1664003	0,03534472
ENSG00000140386	SCAPER	0,47164064	0,03539173
ENSG00000198887	SMC5	0,22498271	0,03546517
ENSG00000176845	METRNL	-0,649221	0,03560586
ENSG00000134255	CEPT1	-0,1965189	0,0356354
ENSG00000267283	AC005306.1	-0,0363289	0,03565331
ENSG00000007545	CRAMP1	-0,3017375	0,03566258
ENSG00000149091	DGKZ	-0,2946591	0,03566274
ENSG00000157181	C1orf27	0,42758373	0,03573993
ENSG00000251194	AL133330.1	0,07752621	0,03574292
ENSG00000204152	TIMM23B	0,17556166	0,03578838
ENSG00000253112	AC102945.1	0,01443183	0,03579379
ENSG00000107175	CREB3	0,40220389	0,03583363
ENSG00000008130	NADK	-0,2159966	0,03589199
ENSG00000257913	DDN-AS1	0,15685824	0,03595497
ENSG00000150867	PIP4K2A	-0,8561387	0,0360009
ENSG00000121851	POLR3GL	-0,4154523	0,03604021

ENSG00000263753	LINC00667	-0,3356875	0,03615901
ENSG00000214826	DDX12P	0,05906065	0,03617689
ENSG00000166272	WBP1L	-0,5127897	0,03621324
ENSG00000149428	HYOU1	-0,5843856	0,03638792
ENSG00000254389	RHPN1-AS1	-0,0861971	0,03646494
ENSG00000144559	TAMM41	-0,4265569	0,03647284
ENSG00000037042	TUBG2	0,17660086	0,03647661
ENSG00000145214	DGKQ	-0,2779234	0,0365051
ENSG00000148362	PAXX	0,38401488	0,03655809
ENSG00000105185	PDCD5	0,33574737	0,03668633
ENSG00000119139	TJP2	0,49350264	0,03675828
ENSG00000168936	TMEM129	-0,3146064	0,0368367
ENSG00000261604	AC114947.2	0,28329674	0,03690425
ENSG00000179021	C3orf38	-0,2737031	0,03695119
ENSG00000174231	PRPF8	-0,5009302	0,03697868
ENSG00000224810	AL355482.1	0,04078046	0,03708076
ENSG00000272602	ZNF595	0,30814214	0,03709391
ENSG00000167645	YIF1B	-0,5144808	0,03712262
ENSG00000178562	CD28	0,09265037	0,03715527
ENSG00000078140	UBE2K	0,31772925	0,03717611
ENSG00000267607	AC011511.5	0,08037702	0,03718991
ENSG00000100490	CDKL1	0,06748368	0,03724531
ENSG00000176124	DLEU1	0,30837332	0,03732746
ENSG00000205238	SPDYE2	0,25759127	0,03738544
ENSG00000130768	SMPDL3B	-0,2381442	0,03743136
ENSG00000140694	PARN	-0,2911347	0,03743673
ENSG00000278376	AP004609.3	0,03740821	0,03745041
ENSG00000182175	RGMA	0,02308623	0,03755242
ENSG00000102580	DNAJC3	-0,4205413	0,03767186
ENSG00000259004	LINC02285	-0,1360558	0,03785536
ENSG00000026025	VIM	0,90664333	0,03787299
ENSG00000197969	VPS13A	0,32412397	0,03791027
ENSG00000065665	SEC61A2	0,13310635	0,03794057
ENSG00000158710	TAGLN2	0,54940575	0,03797774
ENSG00000255165	AC134775.1	0,00838112	0,03800493
ENSG00000271780	AL118558.3	0,0407173	0,03802613
ENSG00000266378	AC005224.3	0,14502943	0,03804761
ENSG00000254017	IGHEP2	0,15303988	0,03805058
ENSG00000178381	ZFAND2A	0,64030228	0,03808946
ENSG00000120875	DUSP4	1,23275884	0,03814569
ENSG00000137166	FOXP4	0,20884218	0,03815763
ENSG00000262791	AC130343.1	-0,0861728	0,038396
ENSG00000249699	LINC02261	0,01573339	0,0384816

ENSG00000205084	TMEM231	0,37814405	0,03855421
ENSG00000167716	WDR81	-0,5306554	0,03856787
ENSG00000278949	AC127070.5	0,1522524	0,03866671
ENSG00000260460	AL365181.1	0,01393174	0,03870879
ENSG00000071909	MYO3B	0,01393174	0,03870879
ENSG00000141682	PMAIP1	0,45760304	0,03875585
ENSG00000274516	FAM74A6	0,0068964	0,03877262
ENSG00000261687	AC068724.2	0,03033587	0,03877716
ENSG00000116678	LEPR	-0,4553986	0,03882204
ENSG00000184227	ACOT1	-0,1611661	0,03898928
ENSG00000254721	AP000879.1	-0,0872273	0,03900355
ENSG00000166188	ZNF319	-0,3309743	0,03907202
ENSG00000119048	UBE2B	0,23328122	0,03912088
ENSG00000065882	TBC1D1	-0,4705268	0,03914715
ENSG00000215246	AC116351.1	0,0337762	0,0391896
ENSG00000205250	E2F4	-0,4690416	0,03919406
ENSG00000165637	VDAC2	0,50955633	0,03920384
ENSG00000110013	SIAE	0,12022802	0,03921739
ENSG00000091640	SPAG7	0,2430339	0,03932161
ENSG00000106462	EZH2	0,4856912	0,03936004
ENSG00000100526	CDKN3	0,15891345	0,03941868
ENSG00000088298	EDEM2	0,2065392	0,03942004
ENSG00000160058	BSDC1	0,26678366	0,03945997
ENSG00000162373	BEND5	-0,1066324	0,03949573
ENSG00000006015	REX1BD	0,38169595	0,03951267
ENSG00000159792	PSKH1	-0,3073569	0,03955469
ENSG00000243696	AC006254.1	0,04309716	0,0396344
ENSG00000135766	EGLN1	-0,2358212	0,03977846
ENSG00000276334	AL133243.2	0,13684728	0,03984804
ENSG00000186654	PRR5	0,05958705	0,03987224
ENSG00000122223	CD244	-1,0676703	0,03989
ENSG00000074621	SLC24A1	0,18645361	0,0399726
ENSG00000117394	SLC2A1	0,69912569	0,04009913
ENSG00000075415	SLC25A3	0,37880648	0,04014309
ENSG00000154001	PPP2R5E	-0,4255688	0,04015682
ENSG00000149716	ORAOV1	-0,0356971	0,04016648
ENSG00000260088	AL445483.1	0,02391016	0,04017168
ENSG00000139835	GRTP1	0,01650461	0,04022481
ENSG00000162194	LBHD1	-0,1677108	0,04030163
ENSG00000247077	PGAM5	-0,1622815	0,04033221
ENSG00000104731	KLHDC4	0,29655159	0,04033842
ENSG00000100055	CYTH4	-0,302901	0,0403444
ENSG00000163749	CCDC158	0,02044169	0,04057276

ENSG00000091536	MYO15A	0,1258601	0,0406492
ENSG00000198960	ARMCX6	0,42972234	0,04083298
ENSG00000139278	GLIPR1	-0,6113268	0,04089799
ENSG00000257950	P2RX5-TAX1BP3	0,15279579	0,04107097
ENSG00000134516	DOCK2	-0,5756712	0,04108147
ENSG00000284116	AL772307.1	-0,0715467	0,04120242
ENSG00000162377	COA7	-0,5043187	0,04128064
ENSG00000135747	ZNF670-ZNF695	0,01660264	0,04134229
ENSG00000259075	POC1B-GALNT4	0,37391514	0,04135221
ENSG00000099715	PCDH11Y	0,00933438	0,04136454
ENSG00000128655	PDE11A	0,00693762	0,04136869
ENSG00000147117	ZNF157	0,00693762	0,04136869
ENSG00000252118	RNU6ATAC39P	0,00693762	0,04136869
ENSG00000258123	LINC02444	0,11188568	0,04139579
ENSG00000280395	AL034546.1	0,02491712	0,0414258
ENSG00000117016	RIMS3	0,19927084	0,04142682
ENSG00000156968	MPV17L	0,21211529	0,04143355
ENSG00000230454	U73166.1	0,01788277	0,04146108
ENSG00000174405	LIG4	-0,3081153	0,04153702
ENSG00000136758	YME1L1	-0,2867643	0,04153947
ENSG00000262202	AC007952.4	0,18757419	0,04154038
ENSG00000183943	PRKX	0,30289095	0,04160778
ENSG00000130518	KIAA1683	0,09063299	0,0416243
ENSG00000136463	TACO1	-0,2292126	0,04165099
ENSG00000154174	TOMM70	-0,3131391	0,04165396
ENSG00000000003	TSPAN6	0,04941891	0,04171185
ENSG00000187699	C2orf88	-0,0565328	0,04181154
ENSG00000226696	LENG8-AS1	0,03704612	0,04183411
ENSG00000277150	F8A3	-1,4132544	0,0418537
ENSG00000184500	PROS1	-0,4615967	0,04190789
ENSG00000164292	RHOBTB3	-0,3650819	0,04196181
ENSG00000234290	AC116366.1	0,58343126	0,04198767
ENSG00000255458	AC108471.2	0,05844795	0,04202275
ENSG00000143294	PRCC	-0,2024051	0,04203961
ENSG00000227908	FLJ31104	0,04716043	0,0421665
ENSG00000224093	AL109613.1	0,0560056	0,04220713
ENSG00000140553	UNC45A	0,4488949	0,04252024
ENSG00000231889	TRAF3IP2-AS1	-0,0519889	0,04256038
ENSG00000266340	AC138207.7	0,45654098	0,04258467
ENSG00000154678	PDE1C	0,0136512	0,04260667
ENSG00000275538	RNVU1-19	0,14500965	0,04262258
ENSG00000106404	CLDN15	0,03210539	0,04271245

ENSG00000111011	RSRC2	0,41676235	0,04273909
ENSG00000163660	CCNL1	0,9850241	0,04285871
ENSG00000278600	AC015871.3	0,20354539	0,04287496
ENSG00000139547	RDH16	0,02204754	0,04292037
ENSG00000141252	VPS53	0,29059141	0,04293085
ENSG00000112851	ERBIN	-0,3337546	0,0429406
ENSG00000259185	AC090971.1	-0,075743	0,04296457
ENSG00000183495	EP400	0,20120943	0,0429747
ENSG00000140829	DHX38	0,37976434	0,04306122
ENSG00000142327	RNPEPL1	-0,4722329	0,04313178
ENSG00000159176	CSRP1	0,28255532	0,04323294
ENSG00000283674	AC068587.6	0,5742571	0,04335313
ENSG00000177034	MTX3	-0,286821	0,04344092
ENSG00000163626	COX18	-0,300904	0,04345555
ENSG00000130208	APOC1	0,49042209	0,0435442
ENSG00000258430	AL583722.2	-0,1665116	0,04354522
ENSG00000185909	KLHDC8B	0,16851346	0,04359415
ENSG00000164190	NIPBL	0,31606581	0,04361453
ENSG00000133619	KRBA1	0,03874256	0,04362834
ENSG00000198846	TOX	-0,4196995	0,04366792
ENSG00000099956	SMARCB1	-0,3579276	0,04373639
ENSG00000068120	COASY	-0,1293167	0,04382736
ENSG00000049656	CLPTM1L	-0,425693	0,04385765
ENSG00000237541	HLA-DQA2	-1,1063905	0,04389573
ENSG00000164828	SUN1	0,58925896	0,04395417
ENSG00000196943	NOP9	-0,1572529	0,04408211
ENSG00000166189	HPS6	-0,5735459	0,04410006
ENSG00000103264	FBXO31	0,23560517	0,04411186
ENSG00000005020	SKAP2	-0,5886589	0,04412304
ENSG00000040933	INPP4A	-0,368616	0,04413029
ENSG00000140678	ITGAX	-0,3967741	0,04419513
ENSG00000169258	GPRIN1	0,23708967	0,04423335
ENSG00000276045	ORAI1	0,71993916	0,04429868
ENSG00000167658	EEF2	-0,5812726	0,0443007
ENSG00000185298	CCDC137	0,16021857	0,04437247
ENSG00000145817	YIPF5	0,11929376	0,04445892
ENSG00000247240	UBL7-AS1	0,07097028	0,04450041
ENSG00000230319	AL022476.1	0,02267257	0,04461129
ENSG00000226853	AC010894.2	-0,0281002	0,04464035
ENSG00000051180	RAD51	0,17368432	0,04465251
ENSG00000162819	BROX	-0,758438	0,04466781
ENSG00000259053	AL137230.2	0,05495376	0,04473294
ENSG00000279500	AC108704.2	0,37710423	0,04477508

ENSG00000158109	TPRG1L	-0,3468632	0,04477836
ENSG00000144741	SLC25A26	-0,2881302	0,04482273
ENSG00000167522	ANKRD11	0,3507242	0,04489094
ENSG00000258476	LINC02207	-0,1973962	0,0449731
ENSG00000064270	ATP2C2	0,04617985	0,04505597
ENSG00000259429	UBE2Q2P2	0,0533317	0,0451117
ENSG00000137962	ARHGAP29	0,00982423	0,04512201
ENSG00000259891	AC107375.1	0,04680453	0,04513882
ENSG00000196850	PPTC7	0,66298847	0,04532692
ENSG00000277400	AC145212.1	0,07917271	0,04535931
ENSG00000180900	SCRIB	-0,0664	0,04554994
ENSG00000169016	E2F6	-0,4156398	0,04557263
ENSG00000244462	RBM12	-0,3590573	0,04560338
ENSG00000115355	CCDC88A	0,45856793	0,04561172
ENSG00000135587	SMPD2	0,69685837	0,0457511
ENSG00000165338	HECTD2	0,17531502	0,0457859
ENSG00000162601	MYSM1	0,16723613	0,04592706
ENSG00000161265	U2AF1L4	0,29268263	0,04594353
ENSG00000128059	PPAT	0,13282159	0,04607862
ENSG00000269924	AC024451.4	0,03509527	0,0460859
ENSG00000104689	TNFRSF10A	0,67101152	0,04616124
ENSG00000267383	AC011447.3	0,04788006	0,04617629
ENSG00000269226	TMSB15B	0,02861634	0,04636156
ENSG00000179918	SEPHS2	-0,6701257	0,04637262
ENSG00000231201	AF127577.2	0,03017618	0,04638471
ENSG00000204310	AGPAT1	-0,3982939	0,0463984
ENSG00000105613	MAST1	0,01977739	0,04644287
ENSG00000156232	WHAMM	0,50100775	0,04644956
ENSG00000079785	DDX1	-0,310317	0,04656344
ENSG00000241956	AC109466.1	0,14384044	0,04656574
ENSG00000099991	CABIN1	0,23758469	0,04660476
ENSG00000204165	CXorf65	0,12026877	0,04666037
ENSG00000249409	AC025741.1	0,03433555	0,04672607
ENSG00000110060	PUS3	0,1334694	0,04678319
ENSG00000233251	AC007743.1	0,47843685	0,04685041
ENSG00000075292	ZNF638	-0,3463439	0,04687608
ENSG00000004478	FKBP4	0,44558272	0,04692575
ENSG00000188112	C6orf132	0,08121354	0,04714058
ENSG00000107882	SUFU	-0,3381038	0,04722511
ENSG00000100629	CEP128	0,69107424	0,04729057
ENSG00000263465	SRSF8	-0,4953364	0,04740933
ENSG00000155304	HSPA13	0,52741687	0,04743608
ENSG00000070047	PHRF1	-0,2111569	0,04748054

ENSG00000262848	AC136624.3	-0,0346389	0,04749779
ENSG00000249228	AC068944.1	0,03665809	0,04754646
ENSG00000189241	TSPYL1	0,4643677	0,04763258
ENSG00000162642	C1orf52	0,27977725	0,04769689
ENSG00000115947	ORC4	0,26186724	0,0479753
ENSG00000115446	UNC50	-0,1909166	0,04812984
ENSG00000205744	DENND1C	-0,5418641	0,04816125
ENSG00000138606	SHF	0,17644255	0,04830046
ENSG00000274828	AC068473.5	0,16562541	0,04837071
ENSG00000161270	NPHS1	0,01574308	0,04850572
ENSG00000264548	AC132872.2	0,02511062	0,04857013
ENSG00000184205	TSPYL2	0,30297106	0,04864869
ENSG00000272983	AL117339.4	0,01949356	0,04872418
ENSG00000120215	MLANA	0,02963986	0,04872458
ENSG00000152056	AP1S3	0,36733512	0,04873599
ENSG00000132128	LRRC41	-0,2557275	0,04876346
ENSG00000257337	AC068888.1	0,06430129	0,04878006
ENSG00000224975	INE1	0,04351987	0,04883137
ENSG00000147394	ZNF185	-0,2576117	0,04893106
ENSG00000166925	TSC22D4	-0,1688658	0,04893598
ENSG00000138867	GUCD1	-0,3081769	0,04895159
ENSG00000011638	TMEM159	0,29805422	0,04896073
ENSG00000157657	ZNF618	0,3071985	0,049011
ENSG00000276293	PIP4K2B	-0,3260235	0,04904859
ENSG00000281657	LINC00976	0,18382651	0,04916988
ENSG00000142621	FHAD1	0,3361367	0,04921691
ENSG00000183624	HMCES	0,52475357	0,0492419
ENSG00000223960	AC009948.1	-0,3137144	0,04937427
ENSG00000077458	FAM76B	-0,3530857	0,04946991
ENSG00000124588	NQO2	0,4933799	0,04950892
ENSG00000111913	RIPOR2	-1,1223126	0,04952251
ENSG00000232629	HLA-DQB2	-1,4754471	0,04961561
ENSG00000124333	VAMP7	-0,2334568	0,04963811
ENSG00000181274	FRAT2	-0,7690678	0,04972066
ENSG00000235852	AC005540.1	0,01520696	0,04976808
ENSG00000281028	AC104662.2	0,16162906	0,04977133
ENSG00000213020	ZNF611	0,15678068	0,04978652
ENSG00000167775	CD320	-0,117217	0,04995948
ENSG00000100605	ITPK1	-0,4581858	0,04998196
